# A conserved cell-pole determinant organizes proper polar flagellum formation

**DOI:** 10.7554/eLife.93004

**Published:** 2024-12-05

**Authors:** Erick E Arroyo-Pérez, John C Hook, Alejandra Alvarado, Stephan Wimmi, Timo Glatter, Kai Thormann, Simon Ringgaard

**Affiliations:** 1 Max Planck Institute for Terrestrial Microbiology, Department of Ecophysiology Munich Germany; 2 Department of Biology I, Microbiology, Ludwig-Maximilians-Universität München Munich Germany; 3 Department of Microbiology and Molecular Biology, Justus-Liebig-Universität Giessen Giessen Germany; 4 Interfaculty Institute of Microbiology and Infection Medicine Tübingen, Bacterial Metabolomics, University of Tübingen Tübingen Germany; 5 Institute for Biological Physics, University of Cologne Köln Germany; 6 Core Facility for Mass Spectrometry and Proteomics, Max Planck Institute for Terrestrial Microbiology Marburg Germany; University of California, Berkeley United States; University of Geneva Switzerland

**Keywords:** vibrio parahaemolyticus, shewanella putrefaciens, *Pseudomonas putida*, Other

## Abstract

The coordination of cell cycle progression and flagellar synthesis is a complex process in motile bacteria. In γ-proteobacteria, the localization of the flagellum to the cell pole is mediated by the SRP-type GTPase FlhF. However, the mechanism of action of FlhF, and its relationship with the cell pole landmark protein HubP remain unclear. In this study, we discovered a novel protein called FipA that is required for normal FlhF activity and function in polar flagellar synthesis. We demonstrated that membrane-localized FipA interacts with FlhF and is required for normal flagellar synthesis in *Vibrio parahaemolyticus*, *Pseudomonas putida*, and *Shewanella putrefaciens*, and it does so independently of the polar localization mediated by HubP. FipA exhibits a dynamic localization pattern and is present at the designated pole before flagellar synthesis begins, suggesting its role in licensing flagellar formation. This discovery provides insight into a new pathway for regulating flagellum synthesis and coordinating cellular organization in bacteria that rely on polar flagellation and FlhF-dependent localization.

## Introduction

Many cellular processes depend on a specific spatiotemporal organization of its components. The DNA replication machinery, cell division proteins, and motility structures are some examples of elements that need to be positioned at a particular site during the cell cycle in a coordinated manner to ensure adequate cell division or proper function of the structures. To carry out this task, many cellular components of bacteria have a polar organization, that is they are asymmetrically positioned inside the cell, which is particularly apparent in monopolarly flagellated bacteria. In the marine bacteria *Vibrio*, which constitutively express a single polar flagellum (McCarter, 1995; Kim and McCarter, 2000), it is crucial to coordinate the localization and timing of flagellar components, in order to guarantee that newly born cells only produce a flagellum in timing with cell division. This process is coordinated with the chromosome replication and the chemotaxis clusters through a supramolecular hub that is tethered at the old cell pole (Takekawa et al., 2016; Yamaichi et al., 2012). This organization depends on ATPases of the ParA/MinD family, which regulate the migration of the hub components to the new cell pole as the cell cycle progresses. In this way, the new cell has a copy of the chromosome and a set of chemotaxis clusters positioned next to what will be the site for the new flagellum. Central to this process is the protein HubP, which recruits to the pole the three ATPases responsible for the localization of these organelles: ParA1 for the chromosome (Fogel and Waldor, 2006), ParC for the chemotaxis clusters (Ringgaard et al., 2011) and FlhG for the flagellum (Correa et al., 2005; Arroyo-Pérez and Ringgaard, 2021). Homologs of HubP occur in several species, for example *Pseudomonas* (where it is called FimV), *Shewanella,* or *Legionella*, where they similarly act as organizers (hubs) of the cell pole (Wehbi et al., 2011; Rossmann et al., 2015; Coil and Anné, 2010).

The bacterial flagellum is a highly intricate and complex subcellular structure. It is composed of around 25 different proteins, which have to be assembled in different stoichiometries in a spatiotemporally coordinated manner (Macnab, 2003; Chevance and Hughes, 2008). Although there may be significant differences among species, flagella in general are composed of a motor attached to the cytoplasmic membrane, a rod connecting it to the extracellular space, a hook and a filament. The basal body is composed of a cytoplasmic C-ring or switch complex (Francis et al., 1994), which receives the signal from the chemotaxis proteins via binding to phosphorylated CheY. The C-ring is connected to the MS-ring, which is embedded in the cytoplasmic membrane (Homma et al., 1987; Ueno et al., 1992). Attached to it, on the cytoplasmic side, is the export apparatus, which allows secretion of rod, hook, and filament components (Minamino, 2014). Flagella can also be sheathed, if an extension of the outer membrane covers the filament, as is the case in many *Vibrio* species (Chen et al., 2017).

The positioning of the flagella and control of flagellar numbers per cell are mediated in many bacteria by the interplay between two flagellar regulators, FlhF and FlhG. Studies in a wide variety of proteobacteria indicate that FlhG is a negative regulator that restricts the number of flagella that are synthesized. In organisms such as strains of *Vibrio*, *Shewanella putrefaciens* and *Pseudomonas aeruginosa*, the absence of MinD-like ATPase FlhG results in hyper-flagellated cells (Kusumoto et al., 2006; Arroyo-Pérez and Ringgaard, 2021; Schuhmacher et al., 2015a; Blagotinsek et al., 2020; Campos-García et al., 2000; Murray and Kazmierczak, 2006). In contrast, in polarly flagellated species such as *Campylobacter jejuni, Vibrio cholerae, Vibrio parahaemolyticus*, *P. aeruginosa, S. putrefaciens* and *Shewanella oneidensis*, the SRP-type GTPase FlhF is a positive regulator of flagellum synthesis and necessary for proper localization. A deletion of *flhF* results in reduced number and mis-localization of flagella (Hendrixson and DiRita, 2003; Correa et al., 2005; Arroyo-Pérez and Ringgaard, 2021; Pandza et al., 2000; Rossmann et al., 2015; Gao et al., 2015; Navarrete et al., 2019; Zhang et al., 2020).

The current model predicts that GTP-bound dimeric FlhF (Bange et al., 2007; Kondo et al., 2018) localizes to the cell pole to where it recruits the initial flagellar building blocks (Green et al., 2009). A long-standing question is how FlhF recognizes and localizes to the designated cell pole. In *P. aeruginosa* and *V. alginolyticus,* it was demonstrated that FlhF localized polarly upon ectopic production in the absence of the flagellar master regulator FlrA (Green et al., 2009; Kondo et al., 2017). It was therefore speculated that FlhF assumes its polar localization in the absence of other flagellar proteins or even without any further additional protein factors. The underlying mechanism, however, remains enigmatic, given that neither in its monomeric nor its dimeric form, FlhF possesses any regions that would indicate a membrane association. In a recent study, it was shown that in the polarly flagellated gammaproteobacterium *S. putrefaciens* FlhF binds the C-ring protein FliG via a specific region at the very N-terminus of FlhF. The FlhF-FliG complex is then recruited to the designated cell pole by HubP, where FlhF-bound FliG captures the transmembrane protein FliF and promotes formation of the MS-ring. This forms the scaffold from where further flagellar synthesis can occur (Dornes et al., 2024). However, in the absence of HubP, the majority of cells still forms normal polar flagella, indicating that HubP is not the only polarity factor in this process (Rossmann et al., 2015).

Here, we provide evidence demonstrating that FlhF does not localize independently. Instead, we show that FipA, a small integral membrane protein with a domain of unknown function, facilitates the recruitment of FlhF to the membrane at the cell pole and, at least in some species, it acts in concert with the polar landmark protein HubP/FimV. Using *V. parahaemolyticus* and two additional polarly flagellated γ-proteobacteria, the monopolarly flagellated *S. putrefaciens* and the lophotrichous *Pseudomonas putida*, we show that FipA universally mediates recruitment of FlhF to the designated cell pole. The spatiotemporal localization behavior of FipA as well as its relationship to FlhF, support its role as a licensing protein that enables flagellum synthesis.

## Results

### Identification of an FlhF protein interaction partner: FipA

**Figure 1.**
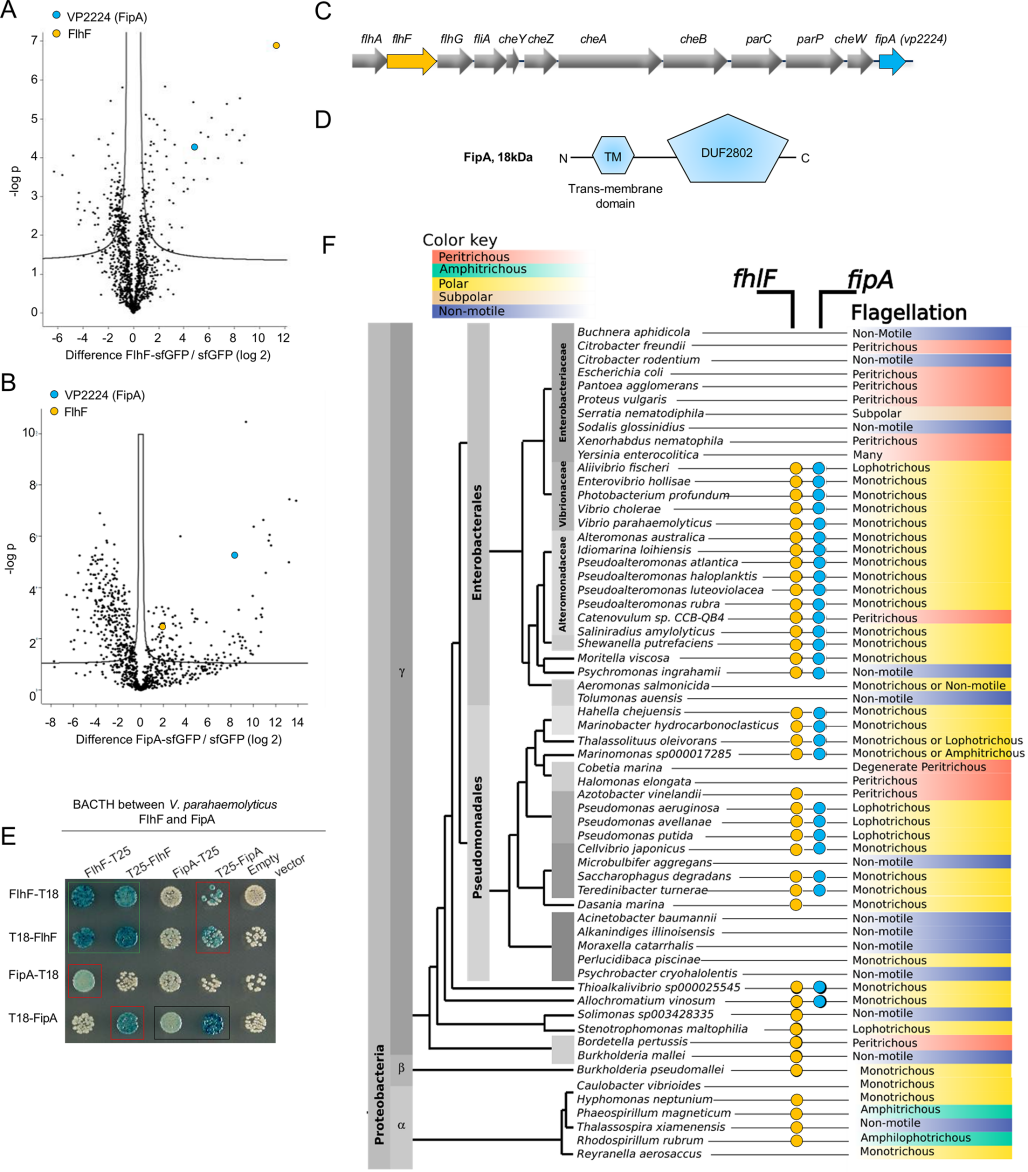
FipA constitutes a new family of FlhF interaction partners. Volcanoplots representing Log-ratios versus significance values of proteins enriched in (**A**) FlhF-sfGFP or (**B**) FipA-sfGFP purifications using shotgun proteomics and liquid chromatography-mass spectrometry; sfGFP was used as control. The full list of pulled-down proteins can be found in the Supplementary file 1a. (**C**) Organization of the flagellar/chemotaxis gene region encoding FlhF and FipA in *V. parahaemolyticus*. (**D**) Domain organization of FipA (TM, transmembrane region; DUF, domain of unknown function). (**E**) Bacterial two-hybrid confirming the interaction between FipA and FlhF from *V. parahaemolyticus*. The indicated proteins (FipA, FlhF) were fused N- or C-terminally to the T18- or T25-fragment of the *Bordetella pertussis* adenylate cyclase. In vivo interaction of the fusion proteins in *Escherichia coli* is indicated by blue color. The corresponding assay in *P. putida* and *S. putrefaciens* is displayed in Figure 1—figure supplement 2. (**F**) Dendrogram of γ-proteobacteria, indicating the presence of FlhF or FipA homologues and the corresponding flagellation pattern. An extended version and sources are available in Supplementary file 1b.

In order to identify potential factors required for FlhF function and its recruitment to the cell pole, we performed affinity purification of a superfolder green fluorescent protein (sfGFP)-tagged FlhF (FlhF-sfGFP) ectopically expressed in wild-type *V. parahaemolyticus* cells followed by shotgun proteomics using liquid chromatography-tandem mass spectrometry analysis (LC-MS/MS). Among the proteins that were significantly enriched in FlhF-sfGFP purifications, eight were structural components of the flagellum (Figure 1A; Supplementary file 1a), but also a non-flagellar protein, VP2224, was significantly co-purified with FlhF (Figure 1A; Supplementary file 1a). The homologue of VP2224 in *V. cholerae*, there named FlrD, was previously shown to exert the same activating role on flagellar gene regulation as FlhF (Moisi et al., 2009), suggesting a function related to FlhF. Notably, FlhF was also significantly co-purified in the reciprocal co-IP-MS/MS experiment using VP2224-sfGFP as bait (Figure 1B), suggesting a direct or indirect interaction of VP2224 and FlhF. To further investigate the co-purification data, bacterial two-hybrid (BACTH) assays in the heterologous host *E. coli* were carried out and suggested a direct interaction between FlhF and VP2224. FlhF and VP2224 were also found to self-interact (Figure 1E). Additionally, these data indicated that FlhF and VP2224 form an interaction complex in both the native and a heterologous host organism. Thus, we identified VP2224 as a novel interaction partner of FlhF and named it FipA for Flh*F i*nteraction *p*artner *A*.

**Figure 1—figure supplement 1.**
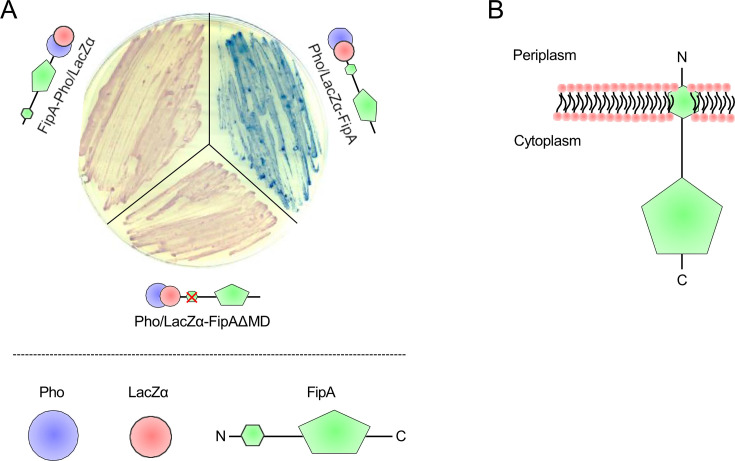
Membrane topology of FipA. (**A**) Topology analysis and (**B**) the corresponding model. A Pho/LacZα cassette was translationally fused to the N- or the C-terminus of FipA and the fusion is expressed in a suitable *E. coli* strain. Pho is only active in the periplasm, while LacZα can functionally complement β-galactosidase activity within the cytoplasm. The enzyme activities can then be tested using suitable color reactions. Expression of Pho-Lac-FipA resulted in blue colonies whereas expression of FipA-Pho-Lac resulted in red colonies, hence suggesting that the FipA N-terminus is positioned in the periplasm and the C-terminus in the cytoplasm (**B**). Deletion of the predicted membrane spanning domain in Pho-Lac-FipA (aa 6–28, Pho-Lac-FipAΔMD) resulted in red *E. coli* colonies (**A**). This suggests that in the absence of the membrane spanning domain the N-terminus of FipA resides in the cytoplasm, and thus further supports that FipA is a transmembrane protein anchored in the inner membrane via the 6–28 aa N-terminal membrane spanning domain and is oriented with the 1–5 aa N-terminal in the periplasm and the 29–163 aa C-terminal in the cytoplasm (**B**).

**Figure 1—figure supplement 2.**
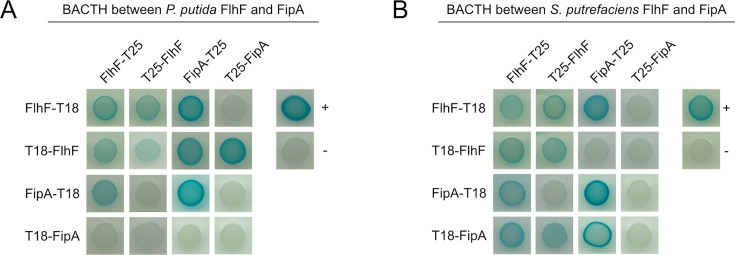
FlhF interacts with FipA in a bacterial two-hybrid analysis (BACTH) in *P. putida* and *S. putrefaciens*. FlhF and FipA were produced as N-terminal or as C-terminal fusions to the T18 and T25 fragments of the *B. pertussis* adenylate cyclase in the given combinations within a single strain. In vivo protein-protein interactions result in active adenylate cyclase activity and high cAMP levels, indicated by blue coloring of the colonies on X-Gal-containing agar.

### FipA constitutes a new family of FlhF interaction partners

The gene encoding FipA is located immediately downstream of the flagellar operon that encodes FlhA, FlhF, FlhG, FliA and the chemotaxis proteins (Figure 1C). In *V. parahaemolyticus*, FipA consists of 163 amino acids with a molecular mass of 18.4 kDa. In silico analysis and membrane topology mapping predicted that FipA consists of a short periplasmic N-terminal part, consisting of amino acids 1–5, followed by a transmembrane region (6-28), a cytoplasmic part harboring a coiled region (amino acids 31–58) and a domain of unknown function, DUF2802, positioned in the C-terminal half of the protein (from amino acids 68–135; Figure 1D; Figure 1—figure supplement 1).

InterPro database analyzes found that FipA homologues (i.e. membrane proteins consisting of a single cytoplasmic DUF2802 repeat) are widespread among γ-proteobacteria. Exceptions are the *Enterobacteriaceae*, which do not possess any copies of either *fipA* nor of *flhF* and *flhG*. Actually, FipA is only present in genomes that also encode FlhF and FlhG (Figure 1F), which prompted the question of whether FipA is involved in regulating the flagellation pattern in concert with the FlhF-FlhG system. By including in our analysis the flagellation pattern reported in the literature, we found that the species that encode FipA are all polar flagellates, either monotrichous or lophotrichous (Figure 1F). FipA homologues are absent from bacteria that use the FlhF-FlhG system to produce different flagellation patterns, like the peritrichous *Bacillus*, the amphitrichous ε-Proteobacteria or Spirochetes (Supplementary file 1b). In the α-Proteobacteria, where FlhF homologues are only present in a few species, FipA is absent as well (Figure 1F). Based on these analyzes, we hypothesized that FipA represents a new family of FlhF interaction partners important for the γ-proteobacteria. To test this hypothesis, we decided to analyze FipA-FlhF interaction in addition in the distantly related and lophotrichously flagellated *P. putida* and the monotrichous *S. putrefaciens*. BACTH analysis showed that the FipA orthologue from both *P. putida* (*Pp*FipA, PP_4331) and *S. putrefaciens* (*Sp*FipA, SputCN32_2550) interact with FlhF of their respective species and that they self-interact (Figure 1—figure supplement 2) – a result similar to that of FipA from *V. parahaemolyticus* (*Vp*FipA). This supported our hypothesis and indicated that FipA has a general function as an FlhF interaction partner, thus constituting a new class of FlhF interaction partners.

### FipA is required for proper swimming motility and flagellum formation

**Figure 2.**
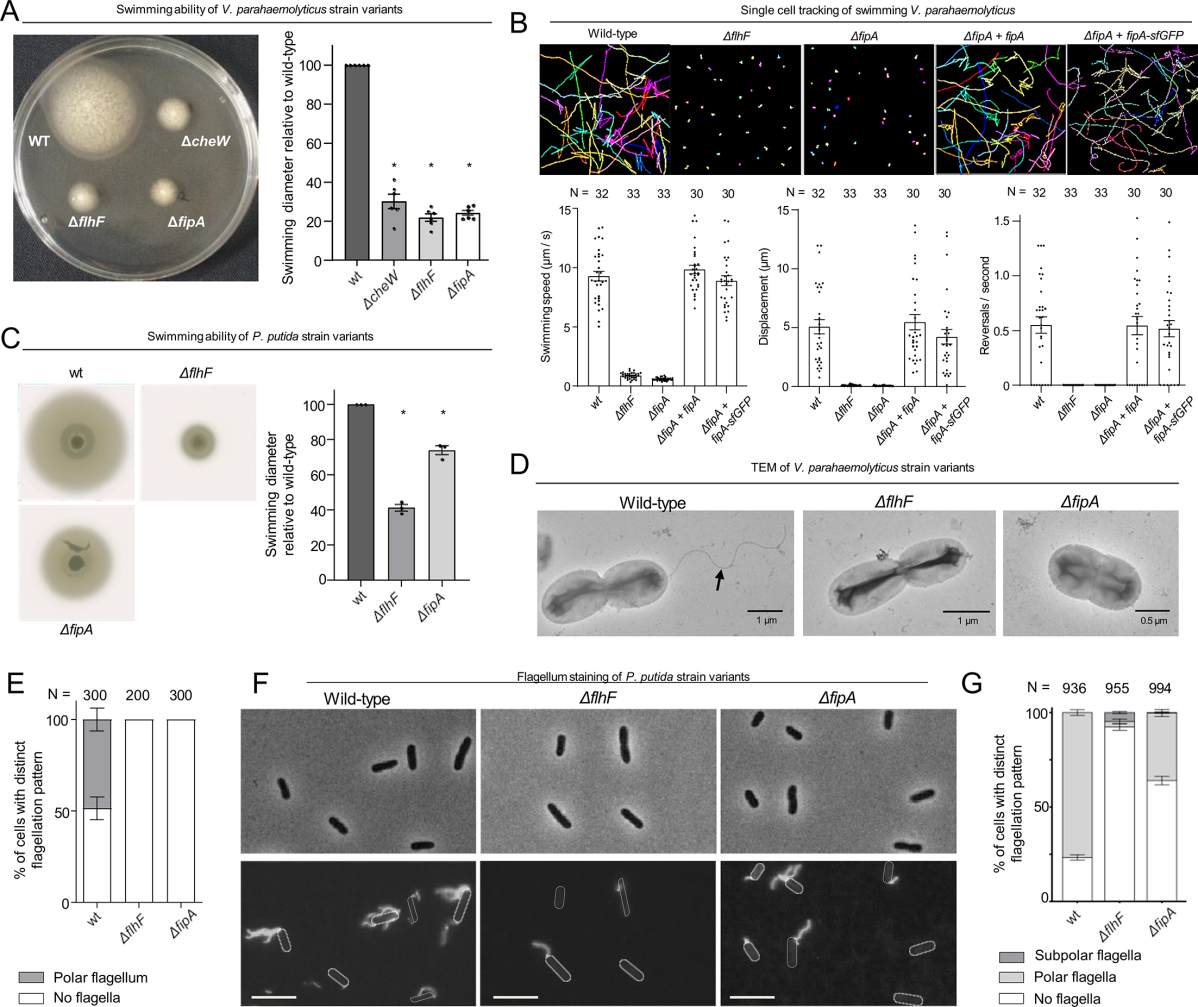
FipA is required for correct flagellum formation. (**A, C**) Representative soft-agar swimming assay of *V. parahaemolyticus* (**A**) or *P. putida* (**C**) strains (left panels) and the corresponding quantification (right panels). For the latter, the halo diameter measurements were normalized to the halo of the wild type on each plate. Data presented are from six (**A**) or three (**C**) independent replicates, asterisks represent a p-value < 0.05 (according to ANOVA + Tukey tests). (**B**) Single-cell tracking of *V. parahaemolyticus*. Shown are representative swimming trajectories and quantification of swimming speed, total displacement and reversal rate. N indicates number of cells tracked among three biological replicates (ANOVA + Tukey test). (**D**) Representative electron micrographs of the indicated *V. parahaemolyticus* strains stained with uranyl acetate. (**E**) Quantification of flagellation pattern in the populations of the indicated *V. parahaemolyticus* strains. (**F**) Flagellum stain of indicated *P. putida* strains with Alexa Fluor 488-C5-maleimide and (**G**) quantification of the corresponding flagellation in the population. N indicates the number of cells counted among three biological replicates. For *S. putrefaciens*, see Figure 2—figure supplement 1.

To explore the role of FipA with respect to flagellation, we generated mutant strains with individual deletions of the *fipA* and *flhF* genes. Strikingly, absence of FipA in *V. parahaemolyticus* completely abolished swimming motility in soft-agar medium to the same degree as cells lacking FlhF (Figure 2A). Furthermore, single-cell tracking of planktonic *V. parahaemolyticus* cells confirmed that cells lacking FipA were completely non-motile and behaved identical to cells lacking FlhF (Figure 2B). Importantly, ectopic expression of FipA in the *ΔfipA* strain restored the strain’s swimming ability to wild-type levels (Figure 2B), further supporting that it is the deletion of *fipA* that results in the phenotype and not polar effects resulting from the *fipA* deletion. The C-terminal FipA-sfGFP fusion used throughout this paper also restored the phenotype (Figure 2B), indicating that the fusion protein is fully functional.

**Figure 2—figure supplement 1.**
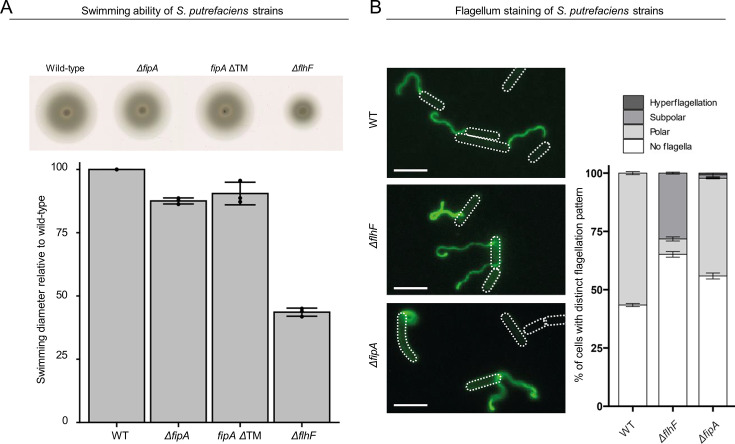
Deletions of or in FipA and FlhF affect *S. putrefaciens* flagellation. (**A**) Upper panel: Spreading of the indicated strains in soft agar. *fipA* ΔTM indicates that the transmembrane region of FipA was deleted. The images shown were compiled from a single plate. Lower panel: the corresponding quantification given as diameter; three independent experiments were conducted. The error bar marks the standard deviation. (**B**) Left: Shown are fluorescent micrographs where the flagellar filament(s) of the indicated strains were fluorescently labeled by maleimide dye. The position of the cell bodies was outlined in the figure. The scale bare equals 5 µm. Right: The corresponding quantification of flagellation. The number of cells (**n**) for each experiment was >300 from three indepenedent experiments. The error bar between the different populations shows the standard deviation.

The swimming phenotypes in the absence of FipA could result from defects in either flagellum assembly or in the flagellar motor performance. To differentiate between these possibilities, we examined *V. parahaemolyticus* by transmission electron microscopy (TEM; Figure 2D and E). Planktonic cells lacking either FlhF or FipA, showed a complete absence of flagella on the bacterial surface, while a single polar flagellum was observed in ~50% of wild-type cells (Figure 2D and E).

Based on this set of experiments, we concluded that FipA and FlhF are essential for normal formation of polar flagella.

### FipA and HubP ensure proper localization of FlhF to the cell pole

As our previous results showed an interaction between FipA and FlhF, we analyzed if the intracellular localization of FlhF was influenced by FipA. In this regard, we used functional translational fusions of FlhF to fluorescent proteins expressed from its native site on the chromosome in *V. parahaemolyticus* (Figure 3—figure supplements 1 and 2; Arroyo-Pérez and Ringgaard, 2021).

**Figure 3.**
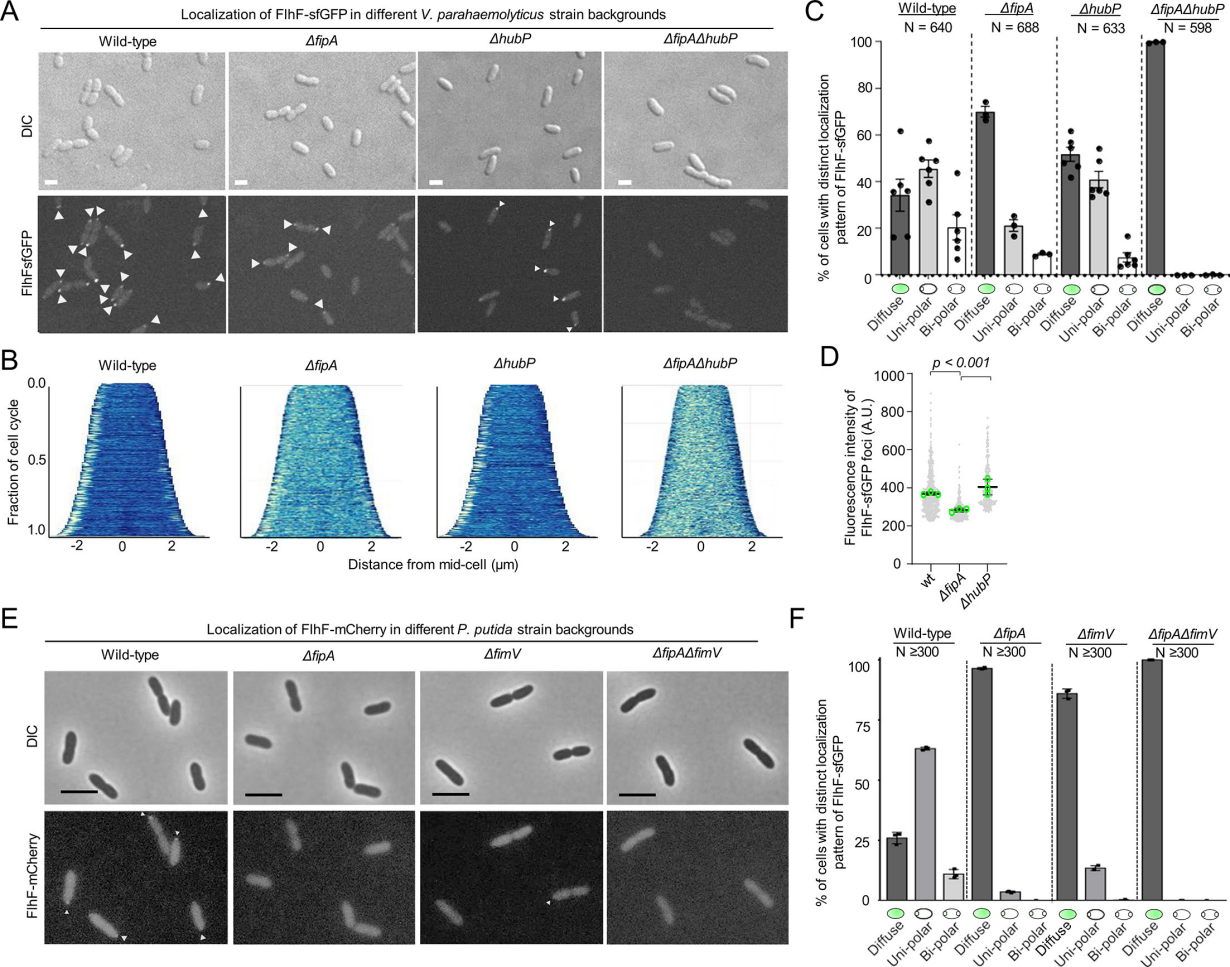
Localization of FlhF depends on FipA and HubP. (**A**) Representative micrographs of the indicated strains of *V. parahaemoloyticus* expressing FlhF-sfGFP from its native promoter. Upper panel shows the DIC image, the lower panel the corresponding fluorescence image. Fluorescent foci are highlighted by white arrows. Scale bar = 2 μm. For an enlargment see Figure 3—figure supplement 3. (**B**) Demographs displaying FlhF-sfGFP fluorescence intensity along the cell length within the experiments shown in (**A**). (**C**) Quantification of localization patterns and (**D**) foci fluorescence intensity of the fluorescence microscopy experiment presented in (**A**). The data was combined from the given number (**N**) of cells combined from three biological replicates. (**E**) Representative micrographs of the indicated *P. putida* strains expressing FlhF-sfGFP from its native promoter. The upper panels show the DIC and the lower panels the corresponding fluorescence images. Fluorescent foci are marked by small white arrows. The scale bar equals 5 µm. The low intensity of the foci did not allow a quantitative analysis of foci intensities or the generation of demographs. For an enlargement of the micrographs see Figure 3—figure supplement 4. (**F**) Quantification of FlhF-sfGFP localization patterns in the corresponding strains of *P. putida* from the experiments shown in (**E**). Corresponding data on the localization of FlhF in *S. putrefaciens* is displayed in Figure 3—figure supplement 5.

We observed that FlhF localized either diffusely in the cytoplasm or to the cell pole as previously reported (Arroyo-Pérez and Ringgaard, 2021). However, a significant delocalization of FlhF from the cell pole occurred in the absence of FipA (Figure 3A–C; Figure 3—figure supplement 2). Particularly, FlhF was diffusely localized in ~37% of cells or localized to the cell pole in a uni- and bi-polar manner in ~45% and~19%, respectively, compared to wild-type cells (Figure 3B). Absence of FipA significantly reduced localization of FlhF to the cell pole with a concomitant increase in diffusely localized FlhF (70%; Figure 3A–C; Figure 3—figure supplement 2). Furthermore, the foci of FlhF at the cell pole in the absence of FipA were significantly dimmer compared to wild-type FlhF foci (Figure 3D), indicating that the amount of FlhF localized to the cell pole is lower in the absence of FipA. Importantly, even though FlhF was still able to localize to the cell pole in a certain number of cells lacking FipA, no flagellum was formed in this background (Figure 2D–E), thus indicating that FipA not only is required for proper polar recruitment of FlhF, but also for the stimulation of flagellum formation.

**Figure 3—figure supplement 1.**
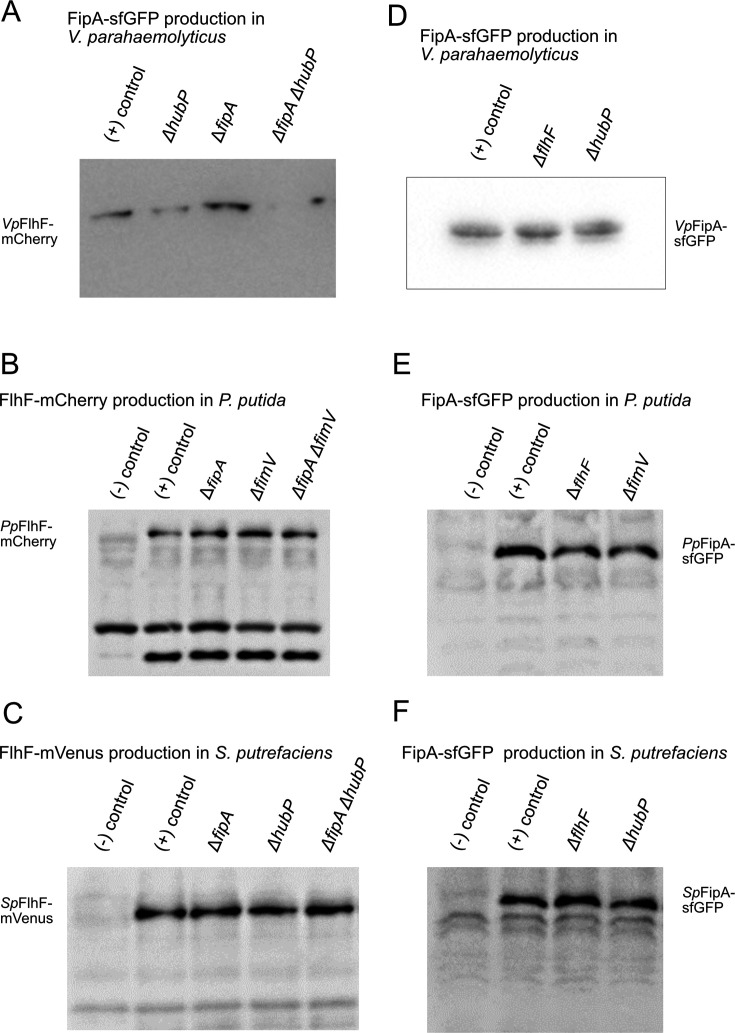
Western analysis on the protein levels of FlhF and FipA fusions to fluorescent proteins. Crude protein extracts from the corresponding (mutant) strains of *V. parahaemolyticus* (**A, D**), *P. putida* (**B, E**) and *S. putrefaciens* (**C, F**) were separated by SDS-PAGE, transferred to membranes and visualized by western blotting using antibodies against GFP (**A, C–F**) and mCherry (**B**). The same OD units from the culture were loaded for each sample. The corresponding strain background is indicated above the lanes. Figure 3—figure supplement 1—source data 1.Scans of the original western blots for Figure 3—figure supplement 1. Figure 3—figure supplement 1—source data 2.Scans of the original western blots for Figure 3—figure supplement 1 with labels.

**Figure 3—figure supplement 2.**
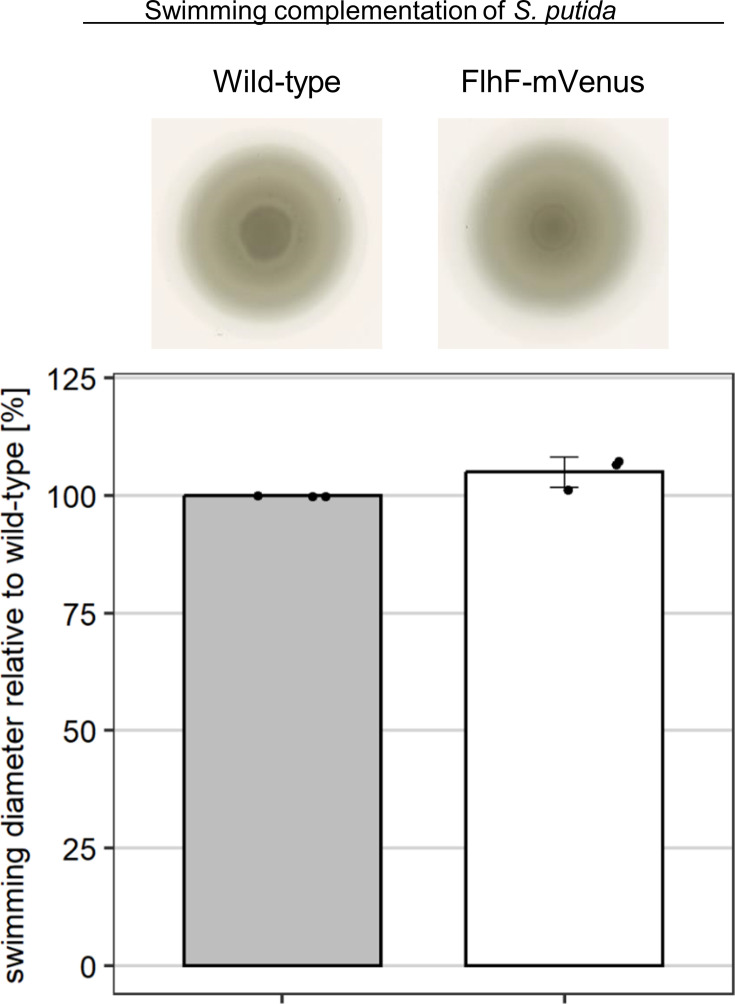
N-terminal tagging of *Pp*FlhF with mCherry does not negatively affect spreading motility in soft agar. The upper panel shows the spreading in soft agar, the lower panel shows the corresponding quantification. The images shown are from the same plate, three independent experiments were conducted. The corresponding standard error bar is shown.

**Figure 3—figure supplement 3.**
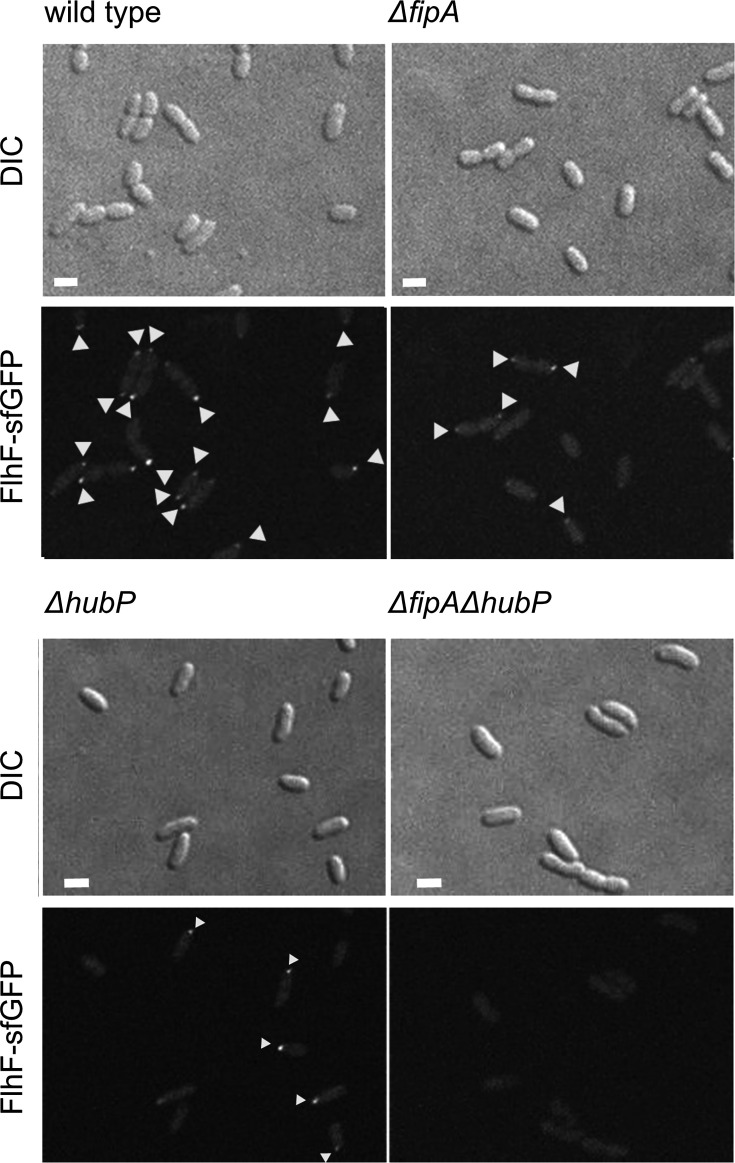
Localization of FlhF depends on FipA and HubP. Shown are enlargments of Figure 3A, representative micrographs of the indicated strains of *V. parahaemoloyticus* expressing FlhF-sfGFP from its native promoter. The upper panel shows the DIC image, the lower panel the corresponding fluorescence image. Fluorescent foci are highlighted by white arrows. Scale bar = 2 μm.

**Figure 3—figure supplement 4.**
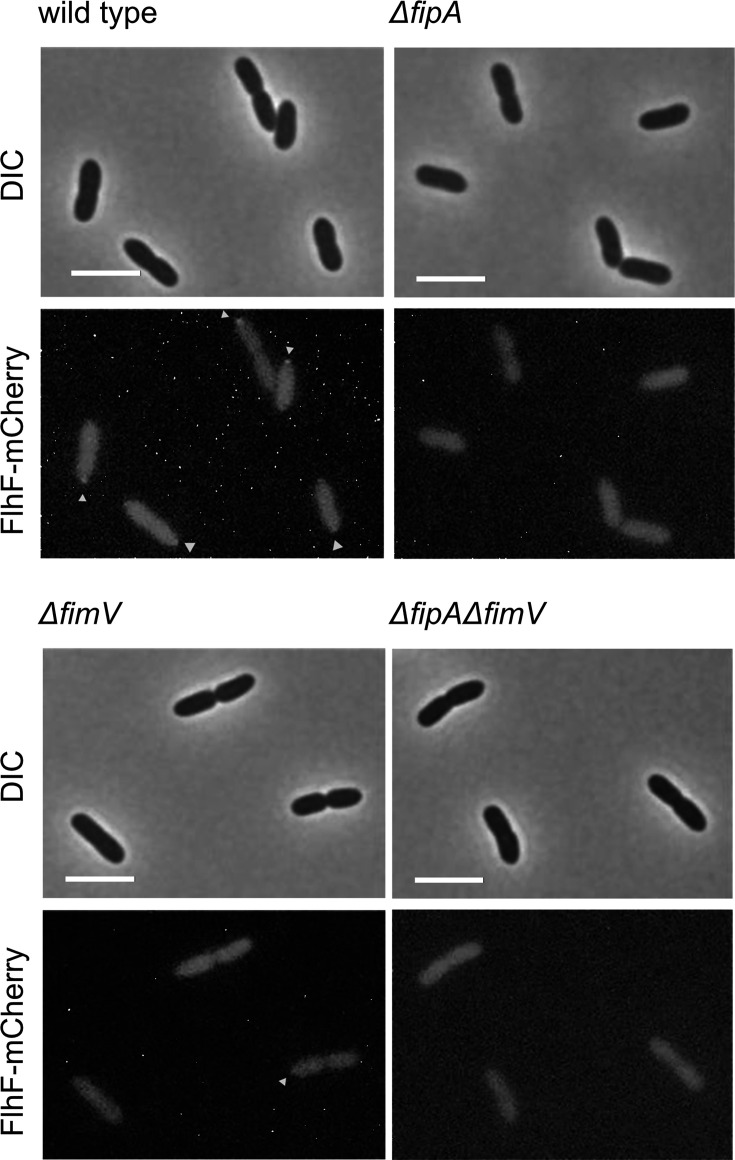
Localization of FlhF depends on FipA and HubP. Shown are enlargments of Figure 3E, representative micrographs of the indicated strains of *P. putida* expressing FlhF-sfGFP from its native promoter. The upper panel shows the DIC image, the lower panel the corresponding fluorescence image. Fluorescent foci are highlighted by white arrows. Scale bar = 5 μm.

**Figure 3—figure supplement 5.**
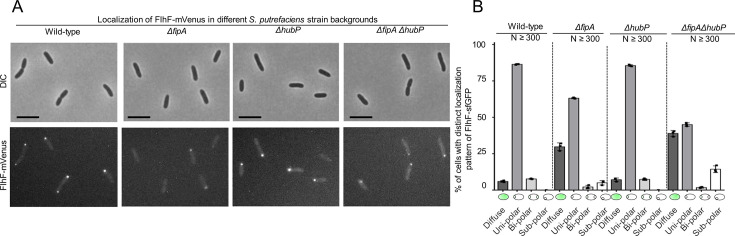
*Sp*FipA and *Sp*HubP concertedly affect polar *Sp*FlhF localization. **A**) Fluorescence microscopy on the indicated (mutant) strains each bearing an *flhF-mvenus* hybrid gene replacing native *flhF* on the chromosome. The upper panel shows DIC micrographs, the lower panel the corresponding fluorescent images. The scale bar equals 5 µm. (**B**) Quantification of the FlhF-mVenus localization patterns, using the data from >300 cells (n) from three independent experiments, the corresponding standard error is shown as error bar.

Given the role of HubP in cell pole organization and its reported interaction with FlhF (Yamaichi et al., 2012), we also analyzed the localization of FlhF in the absence of HubP. As for FipA mutants, recruitment of FlhF to the cell pole was reduced in the absence of HubP. Strikingly, in the double deletion strain *ΔfipA ΔhubP*, FlhF did not localize as foci at the cell pole at all but was instead localized diffusely in the cytoplasm in 100% of cells (Figure 3A–C; Figure 3—figure supplement 3). Immunoblot analysis showed that the difference in localization of FlhF-sfGFP was not due to differences in expression levels or protein stability (Figure 3—figure supplement 1).

Altogether, these data suggest that both FipA and HubP work together to promote normal polar localization of FlhF.

### The transmembrane and conserved cytoplasmic domains of FipA is required for its function in regulating flagellum formation and proper FlhF localization

**Figure 4.**
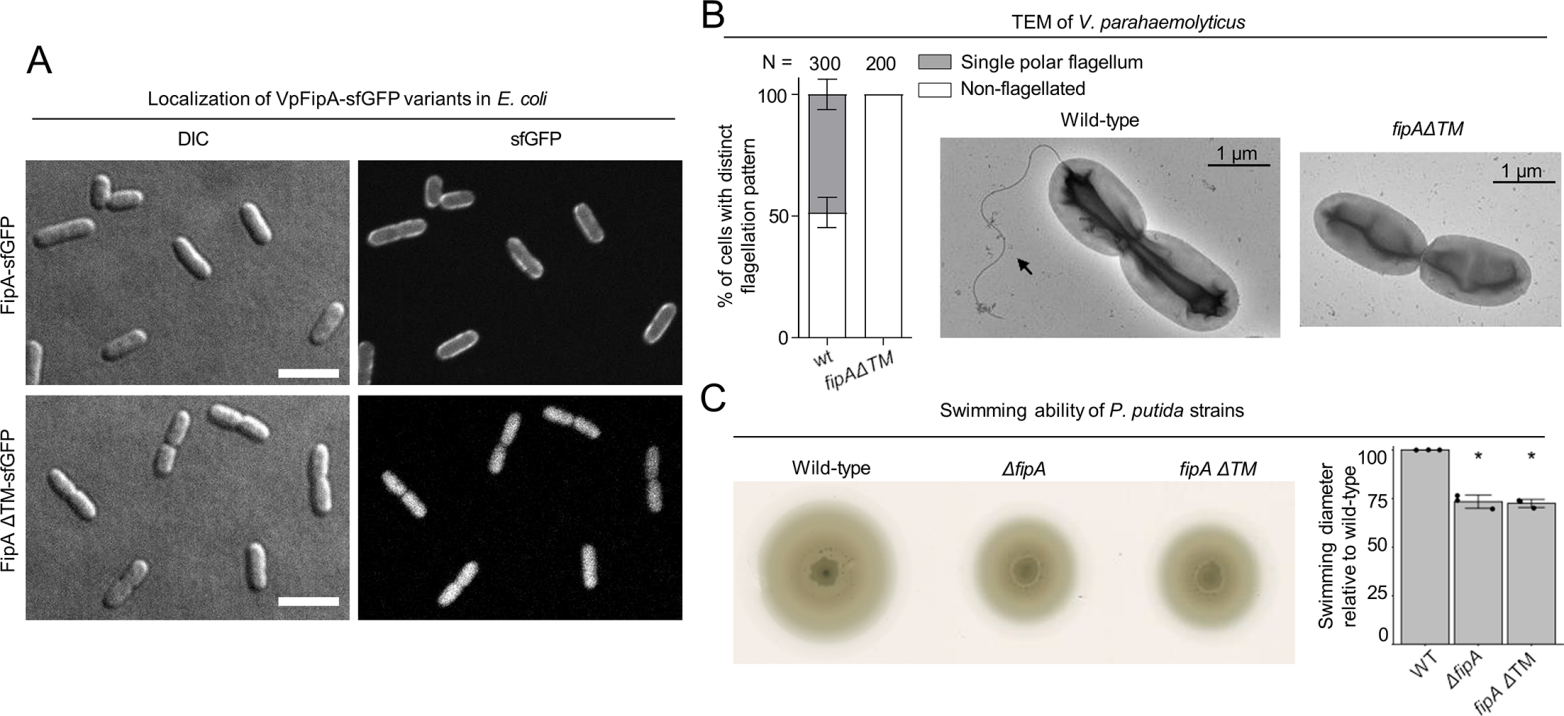
Activity of FipA depends on FlhF and on its transmembrane domain. (**A**) Micrographs of *E. coli* cells expressing FipA-sfGFP from *V. parahaemolyticus*, and a truncated version lacking the transmembrane domain (ΔTM). The left panels display the DIC and the right panels the corresponding fluorescence images. The scale bar equals 5 μm. (**B**) Electron micrographs of *V. parahaemolyticus* wild-type and mutant cells lacking the transmembrane domain of *fipA*, respectively. The corresponding quantification of the flagellation pattern is shown to the left of the micrographs. Note that the data for the wild-type cells is the same as in Figure 2D and E. (**C**) Spreading behavior of the indicated *P. putida* strains (left) with the corresponding quantification (right). Loss of the FipA TM region phenocopies a complete *fipA* deletion.

In order to identify the regions of FipA mediating its role in flagellum regulation, we next analyzed the N-terminal transmembrane domain for FipA membrane anchoring and its function in mediating proper flagellum production. When expressed in *E. coli*, a C-terminally GFP-tagged version of FipA (*Vp*FipA-sfGFP) showed a clear membrane localization, while a FipA variant deleted for the predicted transmembrane (TM) domain between residues 7–27 (FipAΔTM), was diffusely localized in the cytoplasm (Figure 4A). These observations show that FipA is indeed a membrane protein anchored by the predicted transmembrane domain. To then study the function of FipA membrane localization, the native *fipA* locus was replaced by a gene encoding a FipA variant lacking the N-terminal TM domain, and the resulting strain (*fipA ΔTM*) was analyzed for flagellum production. The *V. parahaemolyticus* strain carrying this mutation did not produce any flagella at all (Figure 4B), as did the Δ*fipA* strain (Figure 2D).

**Figure 5.**
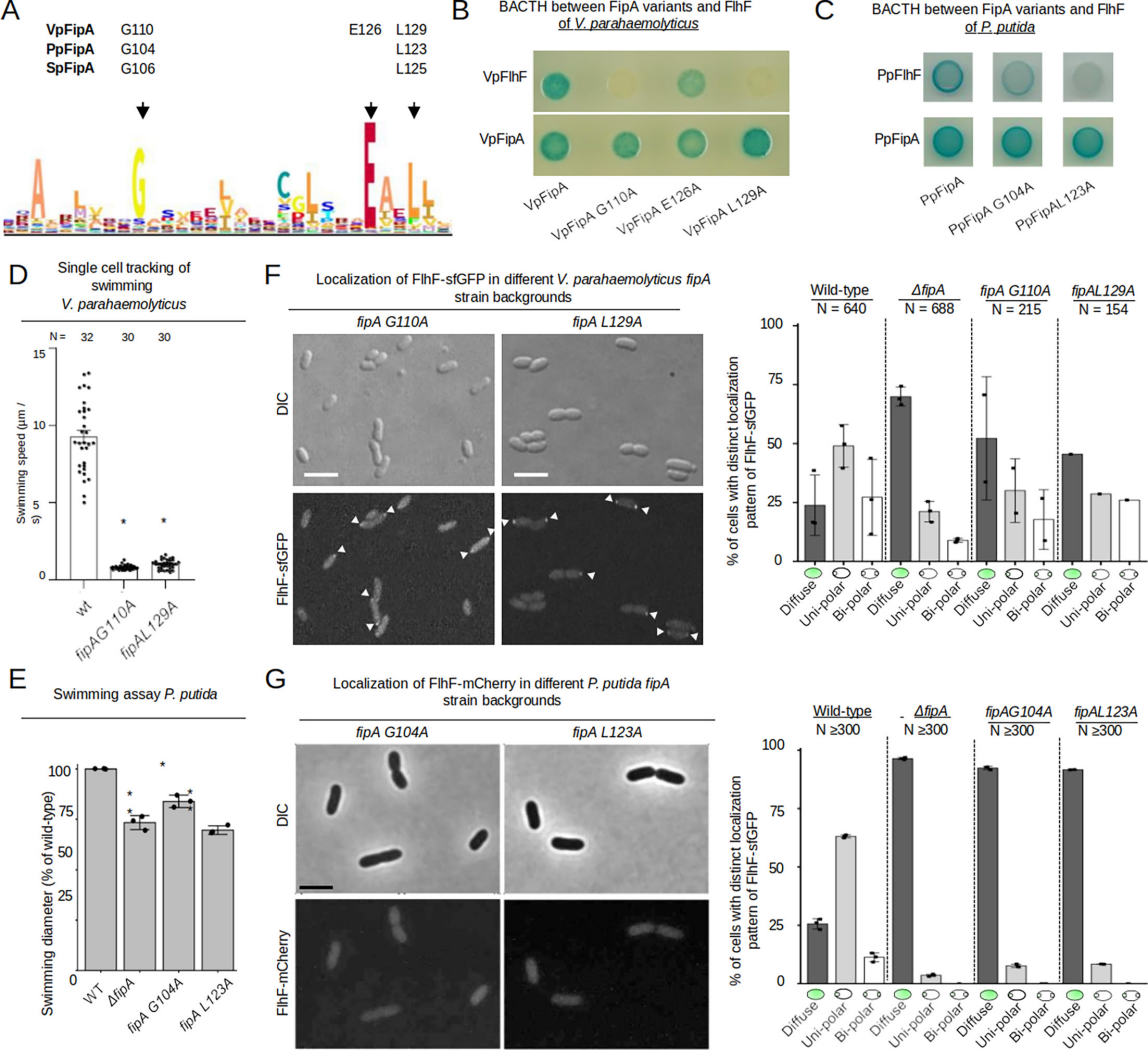
Conserved residues in the domain of unknown function of FipA are essential for interaction with FlhF. (**A**) Weight-based consensus sequence of the conserved region of DUF2802 as obtained from 481 species. The residues targeted in the FipA orthologs of *V. parahaemolyticus*, *P. putida* and *S. putrefaciens* are indicated along with their appropriate residue position. (**B, C**) Bacterial two-hybrid assay of FipA variants of *V. parahaemolyticus* (**B**) or *P. putida* (**C**) with an alanine substitution in the conserved residues indicated in (**A**). The constructs were tested for self-interaction and interaction with FlhF. In vivo interaction of the fusions in *E. coli* is indicated by blue coloration of the colonies. (**D**) Quantification from single-cell tracking of swimming *V. parahaemolyticus* cells expressing FipA bearing the indicated substitution in the DUF2802 domain (see Figure 2B for wild-type behavior). Asterisks indicate a p-value <0.05 (ANOVA + Tukey test) (**F**) Localization of *Vp*FlhF in the absence of FipA or in cells with substitutions in the DUF2802 domain. Left: Micrographs showing the localization of FipA-sfGFP in the indicated strains; the upper panels display the DIC and the lower panels the corresponding fluorescence images (for an enlargement see Figure 5—figure supplement 1). The scale bar equals 5 µm. Right: the corresponding quantification of the FlhF-sfGFP patterns in the indicated strains. (**E**) Soft-agar spreading assays of *P. putida* wild-type and indicated mutant strains, asterisks display a p-value of 0.05 (*) or 0.01 (**) (ANOVA). (**G**) Localization of *P. putida* FlhF in strains bearing substitutions in the DUF2802 interaction site. Left: micrographs displaying the localization of FlhF-mCherry in the indicated strains. Upper panels show the DIC and lower panels the corresponding fluorescence images. The scale bar equals 5 µm (for an enlargement see Figure 5—figure supplement 1). Right: Corresponding quantification of the FlhF localization pattern in the indicated strains. Data for *S. putrefaciens* is displayed in Figure 5—figure supplement 2.

After validating an essential role for membrane anchoring of FipA, we analyzed in more detail the cytoplasmic DUF2802 domain. By aligning various FipA homologs, we identified a motif of conserved amino acids, and three of them (G110, E126 and L129 of *Vp*FipA) were 100% conserved among FipA homologues (Figure 5A). Therefore, we chose these amino acids for mutagenesis. Alanine substitutions of residues G110 and L129 abolished the interaction between *Vp*FipA and *Vp*FlhF in BACTH analysis, while the E126A substitution did not affect the interaction (Figure 5B). All these variants were, however, still able to self-interact with wild-type *Vp*FipA (Figure 5B). Altogether, these results suggest that conserved residues in the DUF2802 domain support the interaction between FipA and FlhF, and that the self-interaction is mediated by a different region.

**Figure 5—figure supplement 1.**
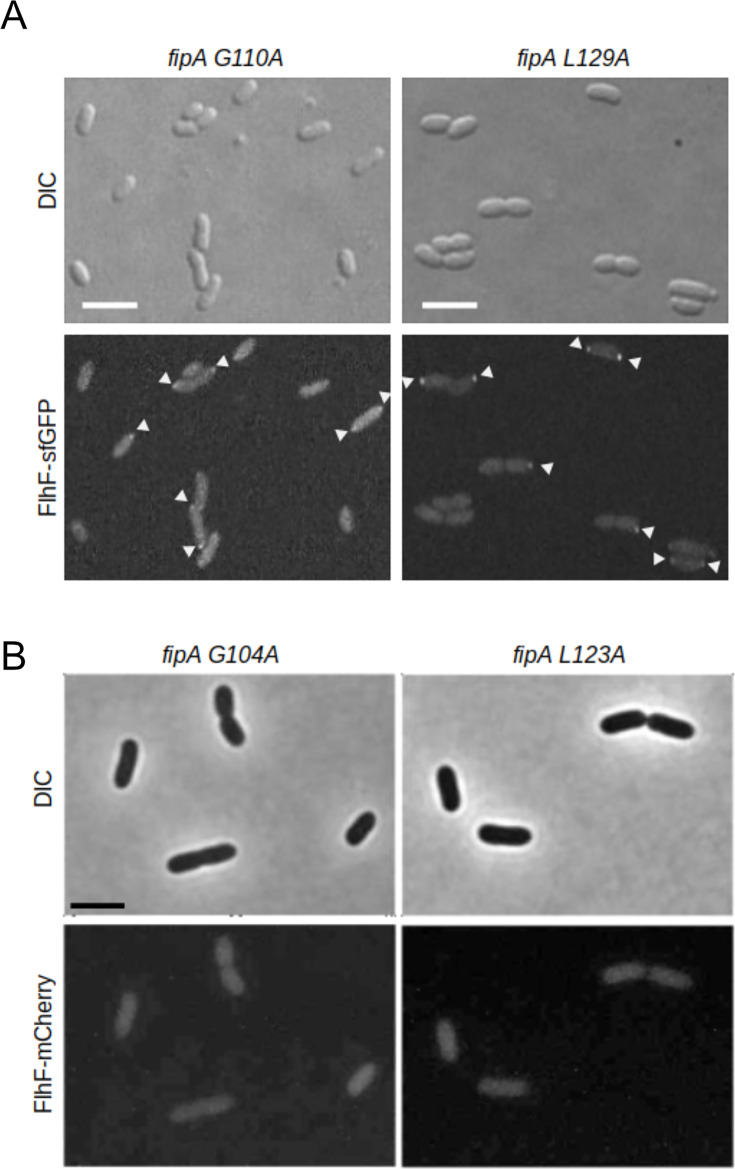
Conserved residues in the domain of unknown function of FipA are essential for interaction with FlhF in *P.* *putida*. (**A**) Localization of *Vp*FlhF in cells with substitutions in the DUF2802 domain. Micrographs are showing the localization of FipA-sfGFP in the indicated strains; the upper panels display the DIC and the lower panels the corresponding fluorescence images. The scale bar equals 5 µm. (**B**) The same analysis for *P. putida* FlhF-mCherry. The images are enlargements of Figure 5F (**A**) and Figure 5G (**B**).

**Figure 5—figure supplement 2.**
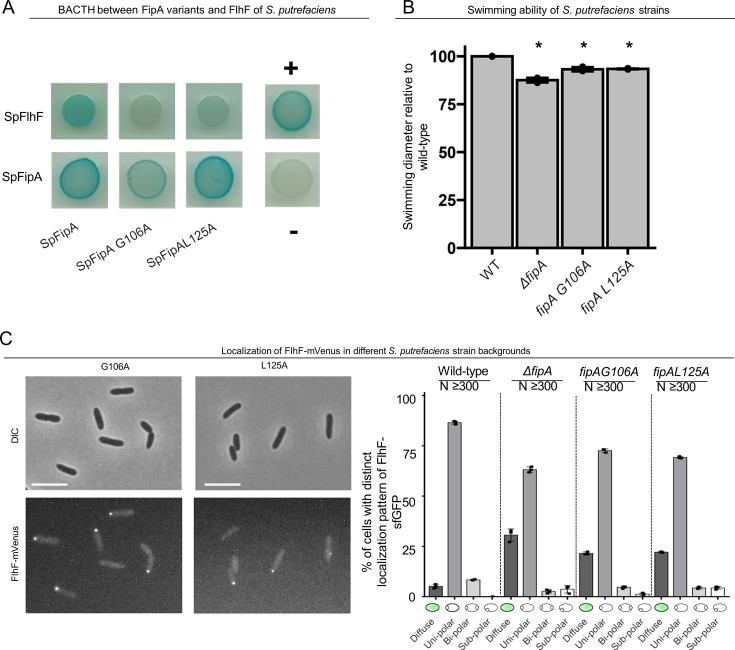
Targeted residue substitution in FipA affects FlhF positioning and function in *S.* *putrefaciens*. (**A**) BACTH analysis (see, e.g. Figure 2) suggests that G106A and L125A in *Sp*FipA does not disrupt but maybe weakens (G106A) the interaction between *Sp*FipA and *Sp*FlhF. (**B**) Spreading of the indicated *S. putrefaciens* strains in soft agar reveals a small but sigifnificant (asterisk = p < 0.05) and based on three independent experiments. (**C**) Left: micrographs showing DIC (upper panels) and fluorescent imaging on mVenus-tagged FlhF cells in mutant backgrounds bearing the given substutions in *Sp*FipA with the corresponding quantification (n>300 cells, right). The in vivo analysis suggests that the residue substitutions in *Sp*FipA give rise to phenotypes that are similar to that of a *fipA* deletion.

### Interaction between FipA and FlhF is required for FipA function on regulating flagellum formation and FlhF localization

We proceeded to evaluate the role of these residues in vivo. After introducing the mutations in the *fipA* gene in its native loci, the effects on motility were almost indistinguishable from a Δ*fipA* mutation. In *V. parahaemolyticus,* this resulted in completely non-motile cells in planktonic cultures (Figure 5D).

The localization of FlhF was also affected by the residue substitutions in *fipA*. Cells of *V. parahaemolyticus* expressing FlhF-sfGFP natively had reduced polar localization when FipA was substituted with either G110A or L129A (Figure 5F; Figure 5—figure supplement 1), with almost 50% of cells presenting only diffuse FlhF-sfGFP signal. Thus, it seems that the DUF2802 domain of FipA is responsible for the effect of FipA on motility, and that its effects occur primarily through the interaction with FlhF. Furthermore, the interruption of FipA-FlhF impedes the recruitment of FlhF to the cell pole.

### Cell cycle-dependent polar localization of FipA

**Figure 6.**
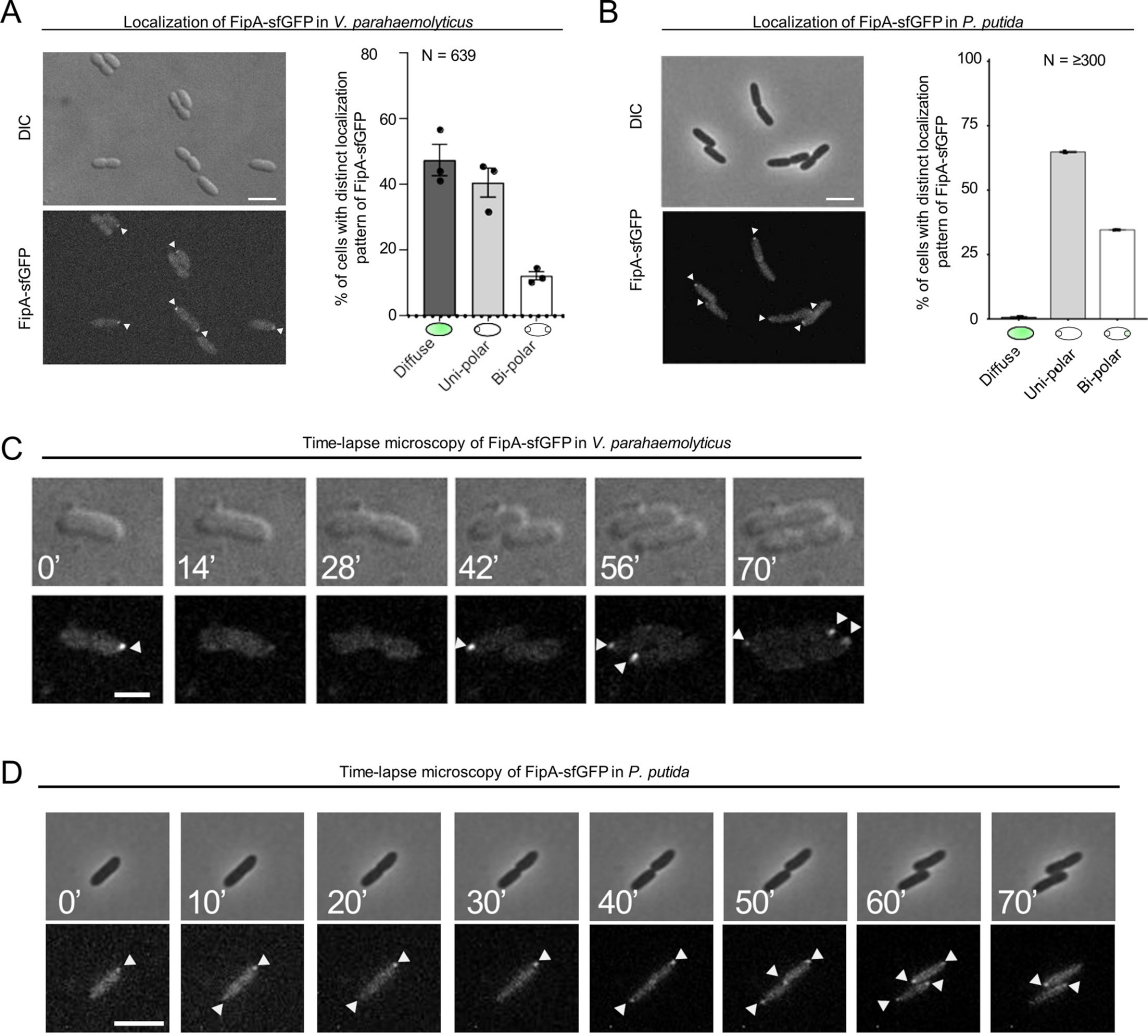
The localization pattern of FipA. (**A, B**) Localization pattern of fluorescently labeled FipA in *V. parahaemolyticus* and *P. putida*. (**A**) Representative micrographs of *V. parahaemolyticus* expressing FipA-sfGFP from its native promoter. Scale bar = 2 μm. The upper panel shows the DIC and the lower panel the corresponding fluorescence channel. To the right the localization was quantified accordingly. (**B**) The same analysis for *P. putida*. (**C, D**) Time lapse analysis of FipA-sfGFP localization over a cell cycle in *V. parahaemolyticus* (**C**) and *P. putida* (**D**). The numbers in the upper DIC micrographs show the minutes after start of the experiment. The scale bars equal 1 µm (**C**) and 5 µm (**D**).

Given FipA’s function in regulating correct recruitment of FlhF to the cell pole, we next analyzed the intracellular localization of FipA. To this end, a hybrid gene bearing a fusion of *fipA* to *sfgfp* was used to replace native *fipA*, resulting in stable and functional production of FipA C-terminally tagged with sfGFP (Figure 6—figure supplement 1). In wild-type strains, FipA localization remained diffuse in half of the population (Figure 6A; Figure 6—figure supplement 1). In the other half of the population, FipA formed distinct foci at the cell pole, either uni- or bi-polarly (Figure 6A; Figure 6—figure supplement 2). No delocalized foci were ever observed. The FipA localization behavior was explored further by following the protein through the cell cycle. We observed that FipA foci disappear frequently (Figure 6C**; 14’**). A new focus appears at the opposite pole, sometimes before cell division (Figure 6C**; 56’**), sometimes after (Figure 6C**; 42’**). On rare cases, the first focus persists until after the second focus appears, resulting in bipolar foci (Figure 6C**; 70’**). These results are consistent with a protein that is recruited to the cell pole right before the start of the flagellum assembly after cell division, albeit the timing may vary between different species.

**Figure 6—figure supplement 1.**
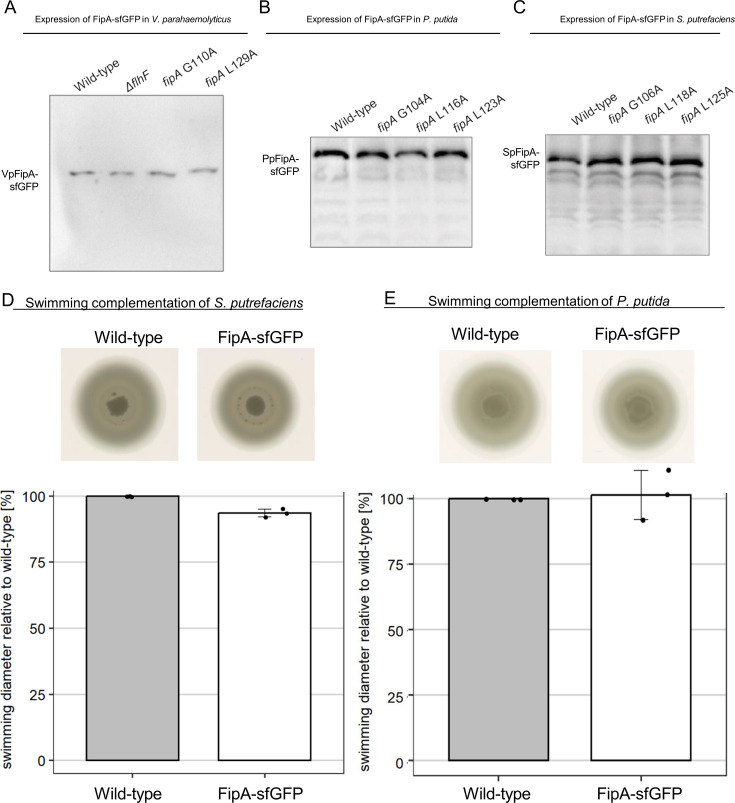
Production and function of FipA derivatives. The three panels show western blotting analysis of PAGE protein separations from *V. parahaemolyticus* (**A**), *P. putida* (**B**) and *S. putrefaciens* (**C**) bearing FipA-sfGFP with substitutions in conserved residues as indicated, using antibodies raised against sfGFP. *V. parahaemolyticus* also includes a mutant deleted in *flhF*. The loaded samples are normalized by OD units. (**D**) The C-terminal fusion of sfGFP has only minor effects on FipA function in *S. putrefaciens* and *P. putida*. The upper panels show the spreading in soft agar (taken from the same soft agar plate), the lower the corresponding quantification. The error bars are the standard deviation from three independent experiments. Figure 6—figure supplement 1—source data 1.Scans of the original western blots for Figure 6—figure supplement 1. Figure 6—figure supplement 1—source data 2.Scans of the original western blots for Figure 6—figure supplement 1 with labels.

**Figure 6—figure supplement 2.**
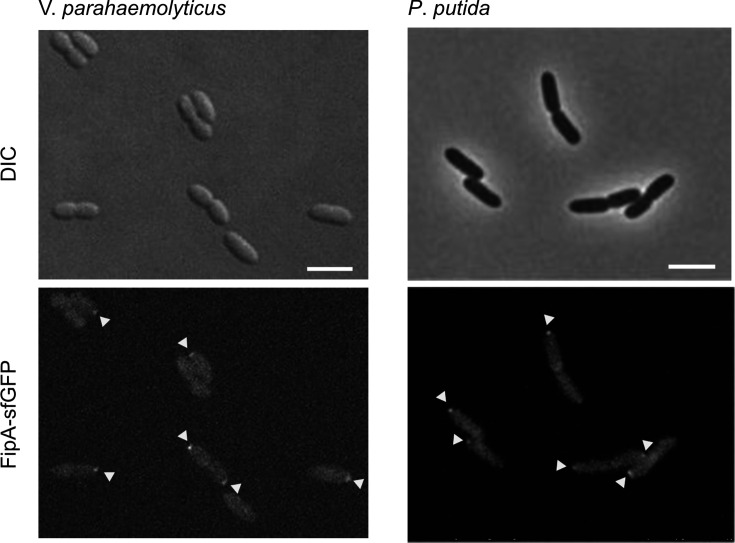
The localization pattern of FipA. Localization pattern of fluorescently labeled FipA in *V. parahaemolyticus* and *P. putida*. Left: Representative micrographs of *V. parahaemolyticus* expressing FipA-sfGFP from its native promoter. Scale bar = 2 μm. The upper panel shows the DIC and the lower panel the corresponding fluorescence channel. Right: The same analysis for *P. putida*. The images are enlargements of the micrographs in Figure 6A and B.

### Polar localization of FipA depends on the FipA-FlhF interaction

**Figure 7.**
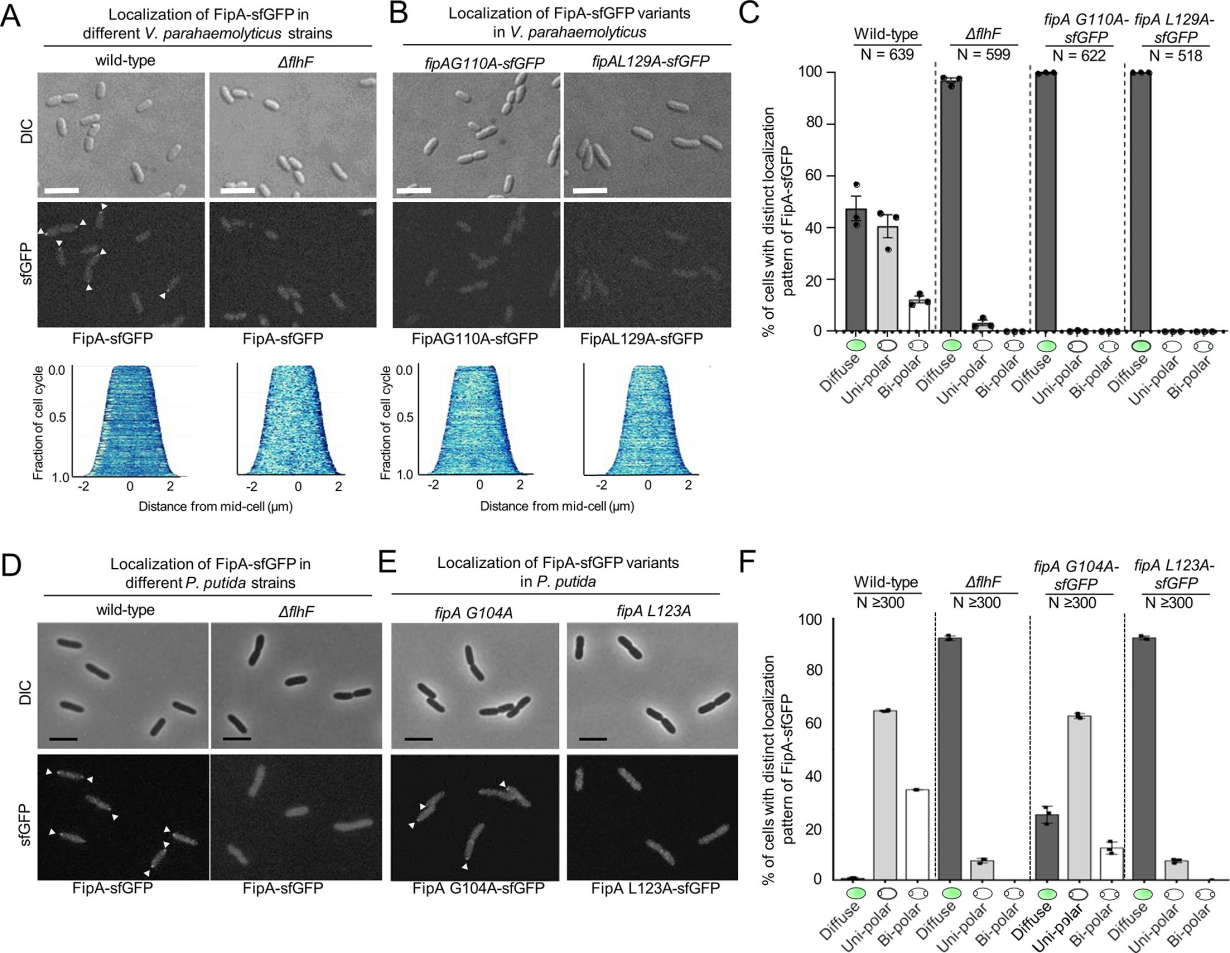
Normal localization of FipA depends on interaction with FlhF. (**A, B**) Localization pattern of *V. parahaemolyticus* FipA-sfGFP in the indicated wild-type and mutant strains. The upper panels display the DIC micrographs, the middle panel the corresponding fluorescence imaging (scale bar equals 5 µm), and the lower panel the corresponding demograph showing the fluorescence of FipA-sfGFP along the cell length. (**C**) Quantification of the cell localization pattern from the experiment shown in (**A, B**) as combined from three biological replicates. (**D, E, F**) The same analysis for the corresponding *P. putida* strains as indicated. The scale bar equals 5 µm. The data for *S. putrefaciens* is displayed in Figure 7—figure supplement 3.

Finally, we determined whether FlhF plays a role in FipA localization. To this end, FlhF was deleted in the strains that are expressing FipA-sfGFP from its native promoter. Almost no FipA foci were detected in this background for both *V. parahaemolyticus* (Figure 7A–C; Figure 7—figure supplement 1) even though the protein levels of FipA were comparable to that in the wild type (Figure 6—figure supplement 1). Furthermore, the FipA variants that do not interact with FlhF (Figure 5B) were also labeled in the native *fipA* locus with sfGFP. Both *Vp*FipA G110A and *Vp*FipA L129A were incapable of forming polar foci (Figure 7B and C; Figure 7—figure supplement 1).

**Figure 7—figure supplement 1.**
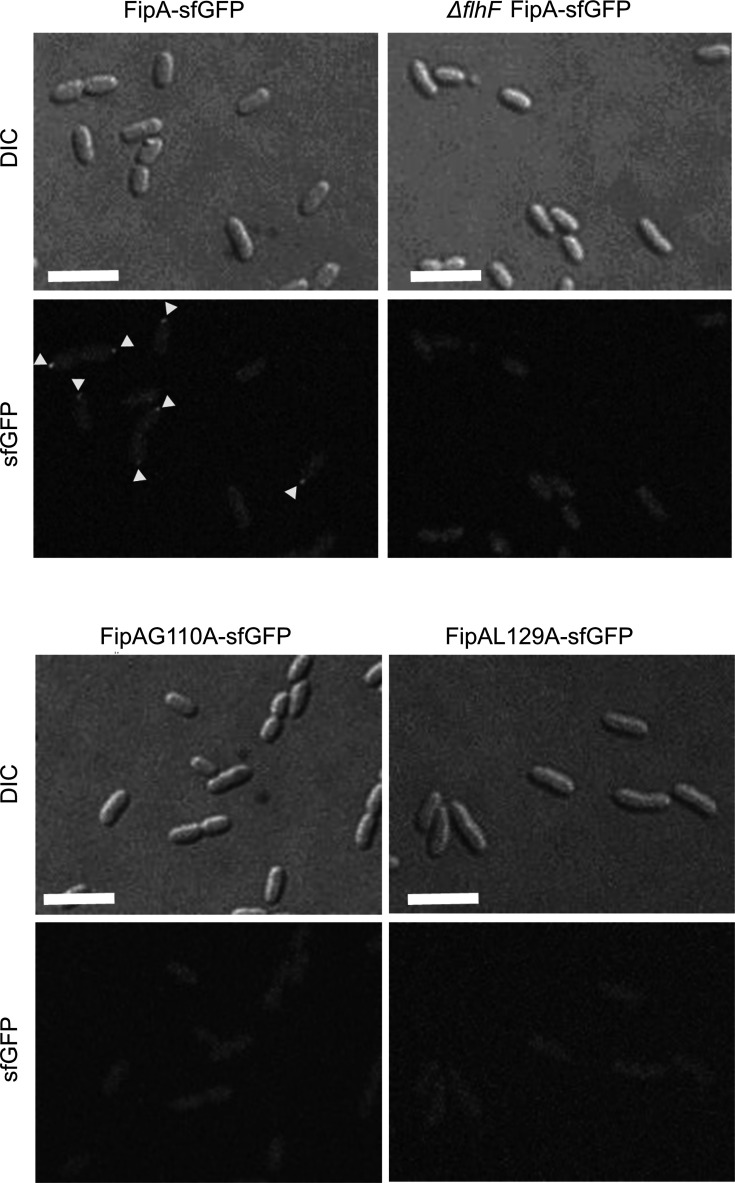
Normal localization of FipA depends on interaction with FlhF. Localization pattern of *V. parahaemolitycus* FipA-sfGFP in the indicated wild-type and mutant strains. The upper panels display the DIC micrographs, the lower panel the corresponding fluorescence imaging (scale bar equals 5 µm). The images are enlargements of the micrographs in Figure 7A (upper) and 7B (lower).

**Figure 7—figure supplement 2.**
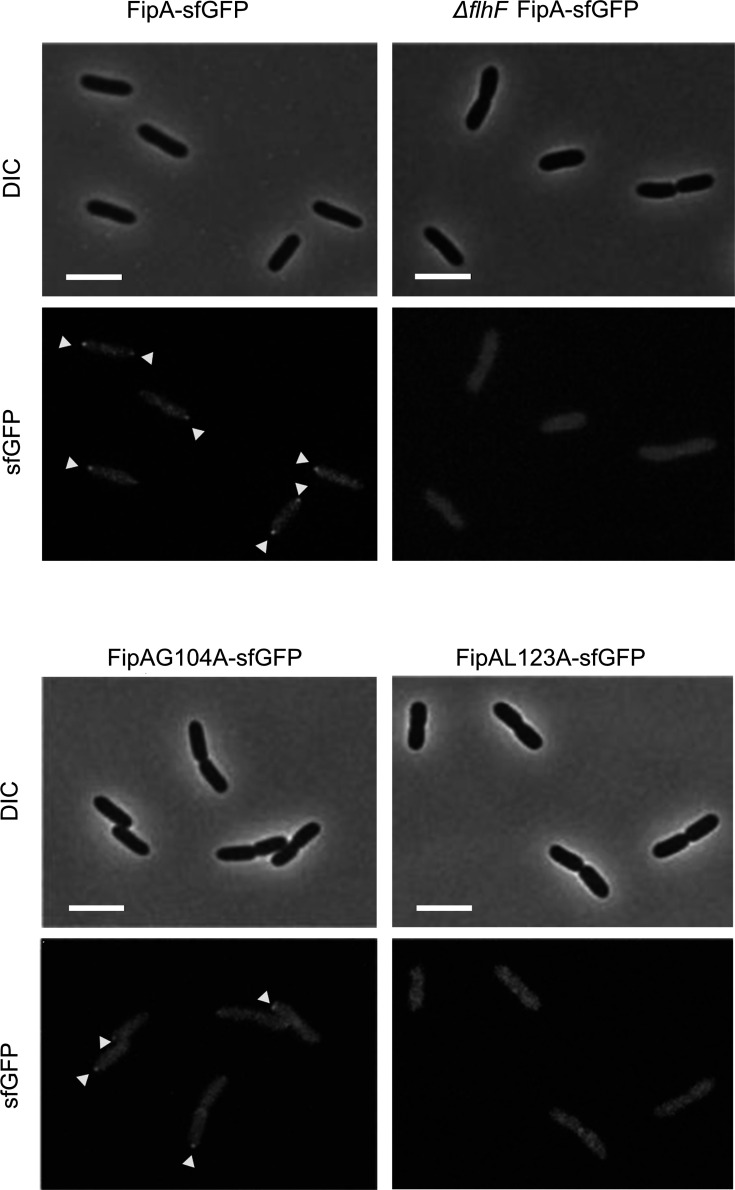
Normal localization of FipA depends on interaction with FlhF. Localization pattern of *P. putida* FipA-sfGFP in the indicated wild-type and mutant strains. The upper panels display the DIC micrographs, the lower panel the corresponding fluorescence imaging (scale bar equals 5 µm). The images are enlargements of the micrographs in Figure 7D (upper) and 7E (lower).

**Figure 7—figure supplement 3.**
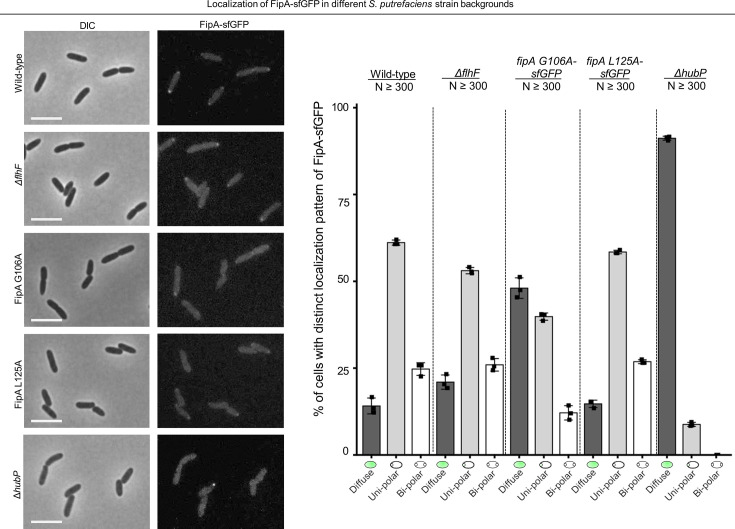
Localization of *Sp*FipA in different strain backgrounds. Left: Micrographs showing DIC (left) and fluorescence (right) microscopy on cells with FipA-sfGFP in different background strains as indicated. The scale bar equals 5 µm. The corresponding quantification (N>300, three independent experiments) is shown to the right. FipA loses its polar localization in a mutant lacking HubP and in a mutant with the L125A substitution in FipA.

Altogether, these results suggest that, in *V. parahaemolyticus*, a direct interaction with FlhF, mediated by the residues in the DUF2802, is responsible for recruiting FipA to the pole.

### Functional conservation of FipA in *S. putrefaciens* and *P. putida*

Our FipA homology analyses (Figure 1F) indicated that the protein is widely conserved in species also possessing FlhF and FlhG. Accordingly, as in *V. parahaemolyticus*, FipA interacted with FlhF in the polarly flagellated gammaproteobacteria *S. putrefaciens*, which is monopolarly flagellated and *P. putida*, which is lophotrichously flagellated (Figure 1—figure supplement 2). We therefore asked to what extent the role of FipA is conserved for flagellation in these two species. To avoid interference with the secondary non-polar flagellar system of *S. putrefaciens*, we used a strain that is unable to form the secondary flagella (*ΔflaAB_2_*). Determination of the flagellation state was carried out by fluorescence labeling of the flagellar filament(s) in both species.

### Spreading

As observed for *V. parahaemolyticus*, loss of FlhF and FipA negatively affected flagella-mediated spreading in soft agar and flagellation of *P. putida* and *S. putrefaciens*. However, for both species, the phenotype was not as severe as in *V. parahaemolyticus*: We observed about 40% and 50% in soft-agar spreading of *ΔflhF* mutants and about 25% and 90% spreading of *ΔfipA* mutants in *P. putida* and *S. putrefaciens*, respectively (Figure 2A and C; Figure 2—figure supplement 1). Accordingly, the mutations of *flhF* or *fipA* also negatively affected flagella number and localization in both species, with the exception of a *fipA* deletion in *S. putrefaciens*, where a dislocalization could not be observed (Figure 2F and G; Figure 2—figure supplement 1). Altogether, these results indicate that FipA is necessary for normal flagellation and swimming motility in all three model species, albeit at a different extent reaching from almost complete loss of swimming in *V. parahaemolyticus* to only a small effect in *S. putrefaciens*.

#### Localization of FlhF

We then determined the localization of FlhF in mutants of FipA and HubP or FimV (the HubP homolog in *P. putida*) by using translational fusions of FlhF to fluorescent proteins expressed from the native site on the chromosome in *P. putida* and *S. putrefaciens* (Figure 3—figure supplements 1 and 2; Rossmann et al., 2015). As observed for *V. parahaemolyticus*, FipA and FimV were required for normal FlhF-mCherry localization in *P. putida* (Figure 3E and F; Figure 3—figure supplement 3), and FlhF was completely delocalized in the double mutant *ΔfipA ΔfimV*. In contrast, FlhF localization was not affected in a *ΔhubP* background in *S. putrefaciens* (Figure 3—figure supplement 4; Rossmann et al., 2015). However, in a *S. putrefaciens* mutant lacking *fipA*, monopolar localization of FlhF-mVenus decreased significantly (Figure 3—figure supplement 4). When both *hubP* and *fipA* were deleted in this species, only weak polar localization remained in a minority of cells. Frequently, polar fluorescence was completely lost and FlhF-mVenus was distributed in the cytoplasm. The differences in localization were not due to differences in abundance of FlhF (Figure 3—figure supplement 1).

The results showed that the function of FipA for flagellation and FlhF localization is generally conserved within the three different species. However, the polar landmark HubP appears to be involved in localizing FlhF, in particular in *S. putrefaciens*, where both HubP and FipA need to be deleted to delocalize FlhF from the cell pole.

#### FipA localization pattern

As the next step, we determined the conservation of FipA domains’ function and localization. We found that, as observed in *V. parahaemolyticus*, loss of the FipA transmembrane region (*fipA ΔTM*) phenocopied a *ΔfipA* mutant in *P. putida* and *S. putrefaciens* (Figure 4C, Figure 2—figure supplement 1). Furthermore, substitution of the conserved residues within the FipA DUF domain (G104A and L123A in *P. putida* FipA; G106A and L118A in *S. putrefaciens* FipA) decreased or abolished the interaction with FlhF but not FipA self-interaction (Figure 5C and G; Figure 5—figure supplement 2).

In *P. putida*, similar to *V. parahaemolyticus*, FipA formed distinct foci at the cell pole, either uni- or bi-polarly (Figure 6A and B). A remarkable difference between *P. putida* and *V. parahaemolyticus* is that in *P. putida,* FipA occurred as foci in virtually all cells (Figure 6B), whereas in *V. parahaemolyticus*, FipA remained diffuse in half of the population (Figure 6A). The ratio of bi-polar to uni-polar foci was also greater in *P. putida* (~1:2) than in *V. parahaemolyticus* (~1:5). Furthermore, in *P. putida*, the FipA foci were more stable, and foci at both poles often persisted until cell division. In fact, appearance of the second focus at the new pole frequently occurred right after cell division (Figure 6D**; 50’**), explaining the greater bi-polar to uni-polar ratio of FipA foci in this organism.

Finally, in *V. parahaemolyticus*, the localization but not the stability of FipA is dependent on the interaction with FlhF (Figure 7; Figure 6—figure supplement 1). This was similarly observed for *P. putida*, as the FipA L123A variant, which is unable to interact with FlhF (Figure 1—figure supplement 2), was also mostly distributed in the cytoplasm, whereas the variant FipA G104A did exhibit some polar localization, although reduced (Figure 7E and F; Figure 7—figure supplement 2). This is the variant that did show some interaction with *Pp*FlhF (Figure 5C). These results suggest that, in *V. parahaemolyticus* and *P. putida*, a direct interaction with FlhF, mediated by the residues in the DUF2802, is responsible for recruiting FipA to the pole.

Surprisingly, in *S. putrefaciens* mutants deleted in *flhF*, FipA displayed only a slight difference in polar localization (Figure 7—figure supplement 3). However, a *Sp*FipA G106A substitution reduced the protein’s localization (to ~50% cells with foci). It is likely that in *S. putrefaciens*, HubP is (co-)mediating the localization of FipA, as in the absence of HubP, FipA-sfGFP localization at the cell pole drastically decreases (Figure 7—figure supplement 3). As observed for *V. parahaemolyticus* and *P. putida*, the stability of FipA was not dependent on the presence of FlhF or FipA in *S. putrefaciens* (Figure 3—figure supplement 1).

## Discussion

In bacteria, numerous processes are localized to specific cellular compartments. This is particularly evident in polarly flagellated bacteria, where the intricate flagellar multicomplex needs to be synthesized in a spatiotemporally regulated fashion. Flagellar synthesis is initiated by the assembly of the first flagellar building blocks within the cytoplasmic membrane, which in polar flagellates is targeted to the designated cell pole. In a wide range of bacterial species, the SRP-type GTPase FlhF and its antagonistic counterpart, the MinD-like ATPase FlhG, regulate flagellar positioning and number (Kazmierczak and Hendrixson, 2013; Schuhmacher et al., 2015b). In particular, FlhF has been shown to function as a positive regulator and a major localization factor for the initial flagellar building blocks of polar flagella. Upon production, GTP-bound dimeric FlhF localizes to the cell pole, but the mechanism by which the protein assumes its designated position at the cell pole remained elusive. One candidate protein, the polar landmark HubP (FimV in *Pseudomonas* sp.) had been identified earlier and was shown to interact with FlhF in *V. parahaemolyticus* (Yamaichi et al., 2012; Dornes et al., 2024). Accordingly, polar localization of FlhF is decreased in mutants lacking *hubP* (this study; Rossmann et al., 2015), however, in a number of cells FlhF localized normally and the cells showed normal flagellation. This was indicative that HubP does play a role in functionally recruiting FlhF, but that another factor was still missing. In this study, we identified the protein FipA as a second polarity factor for FlhF.

FipA consists of an N-terminal transmembrane domain and a cytoplasmic region with a conserved domain of unknown function (DUF2802). The corresponding gene is located immediately downstream of the motility operon that includes *flhF* and *flhG*. Notably, FipA is highly conserved among bacteria that use the FlhF/FlhG system to position the flagella, strongly suggesting that FipA has evolved together with FlhF and FlhG to regulate the flagellation pattern. In this study, we therefore investigated three distantly related γ-proteobacteria including one lophotrichous species, *V. parahaemolyticus*, *P. putida* and *S. putrefaciens*, in more detail. We found that FipA is conserved as a regulatory factor of FlhF and that its functions rely on similar features in all three species.

FipA is essential for normal flagellum synthesis, however, the severity of a *fipA* deletion differed among the three species studied. In *V. parahaemolyticus*, a *fipA* deletion phenocopies the loss of *flhF*, and cells no longer synthesize flagella. Notably, in the absence of FipA in *V. parahaemolyticus,* FlhF is still localized at the pole in about a third of the cells. Despite this, these cells were still unable to form flagella, strongly suggesting that occurrence of FlhF at the pole alone is not sufficient to trigger flagellum synthesis in the absence of FipA.

This strict requirement for FlhF has been reported in the closely related *V. alginolyticus* (Kusumoto et al., 2009). On the other hand, in *V. cholerae,* the requirement for FlhF on flagellation is less strict (Green et al., 2009), and in *P. putida* and *S. putrefaciens* even less so: mutants deleted in *flhF* or *fipA* of both species can generally still produce flagella and swim (this work; Rossmann et al., 2015). In *P. putida,* a Δ*fipA* mutant has a phenotype reminiscent of a Δ*flhF* mutant as both drastically decrease the number of flagella, which may also be delocalized in *flhF* mutants (Pandza et al., 2000). In *S. putrefaciens*, deletion of *fipA* decreases the number of flagella to a similar extent as a *flhF* mutation, however, while in the latter case the flagella are frequently delocalized, the flagella remain at a polar postion, when *fipA* is missing. Taken together, these results strongly suggest that FipA does not act as a general polarity factor for the full flagellar machinery, but rather it stimulates the activity of FlhF to initiate polar flagellation.

### FipA and HubP affect polarity of FlhF through different mechanisms

In all three species, FipA directly interacts with FlhF as demonstrated by reciprocal co-immuno precipitations and by bacterial two-hybrid assays. These experiments suggest that FipA is able to dimerize (or oligomerize) and that the FlhF-FipA interaction is dependent on conserved residues in the DUF2802 domain. Furthermore, these residues are not required for the ability of FipA to interact with itself, suggesting that the two processes occur independently and at different protein interfaces. Microscopic and physiological assays showed that direct interaction between FlhF and FipA is required for FlhF targeting and function. In addition, a FipA mutant lacking its N-terminal transmembrane domain was non-functional, indicating that interaction of FlhF and FipA has to take place at the membrane.

In the absence of FipA, polar localization of FlhF was significantly decreased in all three species and almost completely diminished when *hubP* or *fimV* was deleted together with *fipA*. Removing HubP/FimV alone had a similar effect as removing FipA on the localization of FlhF, although to a lesser extent, as more cells still had polar FlhF in the Δ*hubP/fimV* mutant than in the Δ*fipA* mutant. Furthermore, the unipolar to bipolar ratio of FlhF foci increased in the Δ*hubP* mutant compared to the wild-type or the Δ*fipA* background. While the deletion of *fipA* increases the number of cells with diffuse localization, it does not affect the time frame in the cell cycle at which FlhF shifts from unipolar to bipolar in *V. parahaemolyticus*. In contrast, deleting *hubP* delays the time frame in which FlhF assumes a bipolar position. This indicates that HubP and FipA represent two different pathways that stimulate polar localization of FlhF, and that both are required to bring sufficient (active) FlhF molecules to trigger MS-ring formation and start flagellum assembly.

#### Localization of FipA

A notable feature of FipA is its dynamic localization pattern within the cell over the cell cycle in all three species studied, the nature of which is unclear so far. Foci of FipA may appear mono- or bipolarly and frequently, but not necessarily, disappear once the flagellar apparatus, or the flagellar bundle in the case of *P. putida*, is established. It remains to be shown whether the FipA protein is actively moving from one pole to another or if newly formed FipA is recruited to the opposite pole while being degraded at the old position. Notably, in *V. parahaemolyticus* and *P. putida* FipA does not localize polarly in the absence of interaction with FlhF, suggesting that both proteins are recruited as a complex.

In contrast, in *S. putrefaciens,* FipA still localizes to the cell pole also in the absence of FlhF as long as the polar landmark HubP is present. This is indicating a more important role for HubP in this species. Accordingly, it has been shown recently that in *S. putrefaciens* HubP directly interacts with HubP via its NG domain to recruit the initial flagellar building blocks (Dornes et al., 2024). However, also in *S. putrefaciens* active FlhF localizes to the cell pole and flagella are formed in the absence of HubP. The nature of the polar marker that recruits or guides FipA or a FipA/FlhF to the designated pole is still elusive. The discovery of FipA provides a new point where spatiotemporal organization is coordinated. However, it remains to be explored if the complex is anchored through yet another protein, such as the TonB_m_-PocA-PocB complex in *P. putida* (Ainsaar et al., 2019) or through an intrinsic feature of the cell envelope or cytoplasm during cell division.

#### How does FipA upgrade the current model of polar flagellar synthesis?

Based on the findings in different species, our current model of FlhF/FlhG-mediated polar flagellar synthesis predicts, that upon production, GTP-bound dimeric FlhF is localizing or being recruited to the cell pole to where it recruits the first flagellar components to initiate the assembly. The monomeric form of the ATPase FlhG, the FlhF antagonist, is binding to the flagellar C-ring building block FliM and is transferred to the nascent flagellar structure, where it is released and dimerizes upon ATP binding (Schuhmacher et al., 2015a; Blagotinsek et al., 2020). The ATP-bound FlhG dimer can associate with the membrane and interact with the GTP-bound FlhF dimer, thereby stimulating its GTPase activity. This leads to monomerization of FlhF and loss of polar localization (reviewed in Kojima et al., 2020; Schuhmacher et al., 2015b). Dimeric FlhG does also interact with the master regulator of flagella synthesis, FlrA (or FleQ in *Pseudomonas*) and prevents the synthesis of further early flagellar building blocks (Dasgupta and Ramphal, 2001; Blagotinsek et al., 2020; Banerjee et al., 2021). By this, FlhG links flagella synthesis with transcription regulation and effectively restricts to number of polar flagella that are formed. Based on this model, FipA may recruit FlhF to the membrane and stimulate or stabilize GTP binding and dimerization of FlhF to promote polar localization and initiation of flagella synthesis. Thus, the role of FipA is to shift the equilibrium to the active state of FlhF in order to start assembly of the MS ring. Accordingly, current studies address the structural basis of the FipA-FlhF interaction, the potential effect of FipA on GTP-binding and dimerization of FlhF, and the role of the polar marker HubP in this process.

In addition, *fipA* expression is independent of the rest of the flagellar genes in other species of *Vibrio* (Moisi et al., 2009; Petersen et al., 2021) and *S. putrefaciens* (Schwan et al., 2022). Thus, *fipA* transcription could provide another regulatory point to lead to the synthesis of a flagellum. Thus, the discovery of FipA opens several novel open questions in the field of flagellum regulation.

## Materials and methods

### Growth conditions and media

In all experiments, *V. parahaemolyticus* and *E. coli* were grown in LB medium or on LB agar plates at 37 °C containing antibiotics in the following concentrations: 50 μg/mL kanamycin, 100 μg/mL ampicillin and 20 μg/mL chloramphenicol for *E. coli* and 5 μg/mL for *V. parahaemolyticus. S. putrefaciens* CN-32 and *P. putida* KT 2440 were grown in LB medium or LB agar plates at 30 °C. When required, the media were supplemented with 50 µg/mL kanamycin, 300 µM 2,6-diaminopimelic acid and/or 10% (w/v) sucrose.

### Strains and plasmids

The strains and plasmids used in this study are listed in Supplementary file 1c and d, respectively. Primers used are listed in Supplementary file 1e. *E. coli* strain SM10λ*pir* was used to transfer DNA into *V. parahaemolyticus* by conjugation (Miller and Mekalanos, 1988). For DNA transfer into *S. putrefaciens* and *P. putida*, *E. coli* WM3064 was used. *E. coli* strains DH5αλ*pir* and SM10λ*pir* were used for cloning. Construction of *V. parahaemolyticus* deletion mutants was performed with standard allele exchange techniques using derivatives of plasmid pDM4 (Donnenberg and Kaper, 1991). Chromosomal deletions and integrations in *S. putrefaciens* and *P. putida* were carried out by sequential crossover as previously described (Rossmann et al., 2015) using derivatives of plasmid pNPTS138-R6K (Lassak et al., 2010).

### Construction of plasmids

*Plasmid pSW022*: The regions flanking *vp2224* (*fipA*) were cloned with primers VP2224-del-a/ b and VP2224-del-c/ d, using *V. parahaemolyticus* RIMD 2210633 chromosomal DNA as template. The resulting products were fused in a third PCR using primers VP2224-del-a/ VP2224-del-d. The end product was digested with XbaI and ligated in the equivalent site of vector pDM4, resulting in plasmid pSW022. The mutation in *V. parahaemolyticus* was confirmed with a PCR using primers VP2224-del-a/VP2224-check. *Plasmid pPM123:* The regions flanking aa 7–27 of *vp2224* (*fipA*) were cloned with primers VP2224-del-a/ del AA7-27 vp2224-b and del AA7-27 vp2224-c/ VP2224-del-d, using *V. parahaemolyticus* RIMD 2210633 chromosomal DNA as template. The resulting products were fused in a third PCR using primers VP2224-del-a/ VP2224-del-d. The end product was digested with XbaI and ligated in the equivalent site of vector pDM4, resulting in plasmid pPM123. The mutation in *V. parahaemolyticus* was confirmed with a PCR using primers VP2224-del-a/VP2224-check. *Plasmid pPM178:* The gene *vp2224* (*fipA*) was amplified from *V. parahaemolyticus* RIMD 2210633 chromosomal DNA with primers C-term sfGFP-vp2224-a/-b; the downstream region with primers C-term sfGFP-vp2224-e/f; the gene encoding sfGFP with C-term sfGFP-vp2224-c/d from plasmid pJH036. The three products were fused together in another PCR using primers C-term sfGFP-vp2224-a/f. The obtained product, encoding FipA fused in frame to sfGFP via a 5-residue linker, was digested with SpeI and SphI and ligated in vector pDM4 digested with XbaI and SphI, resulting in plasmid pPM178. The mutation in *V. parahaemolyticus* was confirmed with a PCR using primers C-term sfGFP-vp2224-f/VP2224-check. *Plasmid pPM179 & pPM180:* The region upstream of *vp2224* (*fipA*) were amplified as for pSW022. The downstream region was amplified with downstream vp2224-cw/VP2224-del-d from *V. parahaemolyticus* RIMD 2210633. The gene *vp2224* itself was amplified with primers vp2224 cw restore deletion/vp2224 ccw restore deletion, from plasmids pEP005 & pEP006, carrying the mutations G110A and L129A. The products were fused in a third PCR using primers VP2224-del-a/ VP2224-del-d. The end product was digested with XbaI and ligated in the equivalent site of vector pDM4, resulting in plasmids pPM179 & pPM180. The the re-insertion in *V. parahaemolyticus* SW01 was confirmed with a PCR using primers VP2224-del-a/VP2224-check. *Plasmid pPM187 & pPM191:* The insertion of sfGFP at the C-terminus of FipA was cloned with the same strategy for pPM178, but using pPM179 & pPM180 as templates for the *vp2224* sequence, resulting in plasmids pPM191 & pPM187, respectively. *Plasmid pPM146:* The gene *vp2224* (*fipA*) was amplified from *V. parahaemolyticus* RIMD 2210633 chromosomal DNA with primers vp2224-cw-pBAD/vp2224-ccw-pBAD. The product was digested with enzymes XbaI and SphI, and inserted in the corresponding site in plasmid pBAD33. *Plasmid pPM159:* The gene *vp2224* (*fipA*) was amplified from *V. parahaemolyticus* RIMD 2210633 chromosomal DNA with primers C-term sfGFP-vp2224-a/-b, and the gene encoding sfGFP with C-term sfGFP-vp2224-c/sfGFP-1-ccw from plasmid pJH036. The resulting products were fused in another reaction with primers C-term sfGFP-vp2224-a/sfGFP-1-ccw, and this final product was digested with XbaI and cloned in pBAD33. *Plasmid pPM194:* Same as pPM159, but *vp2224 ΔTM* was amplified from pPM123 using primers vp2224 AA1-6/28-end w/o Stop/sfGFP-1-ccw. *Plasmids pSW74 & pSW119:* The region of the gene *vp2224* (*fipA*) encoding the cytoplasmic part (residues 28–163) was amplified with primers tr2224 put18C cw/ pUT18C/pKT25-vp2224-ccw from *V. parahaemolyticus* chromosomal DNA. The product was digested with KpnI and XbaI and ligated in the corresponding site in pKT25, to generate pSW74, and in pUT18C, to generate pSW119. *Plasmid pPM118 & pPM119:* The cytoplasmic part of the gene *vp2224* (*fipA*) was amplified with primers pUT18/pKNT25-tr-vp2224-cw & pUT18/pKNT25-vp2224ccw from *V. parahaemolyticus* chromosomal DNA. The product was digested with KpnI and XbaI and ligated in the corresponding site in pUT18, to generate pPM118, and in pKNT25, to generate pPM119. *Plasmid pPM124 & pPM128:* The gene *vp2234* (*flhF*) was amplified with primers pUT18/pKNT25- vp2234-cw & pUT18/pKNT25-vp2234 -ccw from *V. parahaemolyticus* chromosomal DNA. The product was digested with KpnI and XbaI and ligated in the corresponding site in pUT18, to generate pPM124, and in pKNT25, to generate pPM128. *Plasmid pPM132 & pPM136:* The gene *vp2234* (*flhF*) was amplified with primers pUT18C/pKT25- vp2234-cw & pUT18C/pKT25-vp2234 -ccw from *V. parahaemolyticus* chromosomal DNA. The product was digested with KpnI and XbaI and ligated in the corresponding site in pUT18C, to generate pPM132, and in pKT25, to generate pPM136. *Plasmids pPM160, pPM161 & pPM162:* Site directed mutagenesis was performed on plasmid pSW74 by the QuickChange method (Zheng et al., 2004), using primers vp2224-Gly-110Ala-cw/ccw, vp2224-Glu 126Ala-cw/ccw or vp2224-Leu 129Ala-cw/ccw. After digesting the template with DpnI, the products were transformed into *E. coli* and the mutations were confirmed by sequencing. *Plasmid pPM106:* The gene vp2224 (fipA) was amplified with primers pUT18C/pKT25-vp2224-cw & pUT18C/pKT25-vp2224ccw from V. parahaemolyticus chromosomal DNA. The product was digested with KpnI and XbaI and ligated in the corresponding site in pKTop, to generate pPM106. *Plasmid pPM109 & pPM112:* The *phoA-lacZα* fragment of pKTop was amplified with primers vp2224 C-term PhoA-LacZ cw & end -LacZ w/o STOP ccw. The full length *vp2224(fipA*) gene was amplified from plasmid pPM146 using primers LacZ to vp2224 w/o ATG & end vp2224 ccw. The *vp2224* ΔTM allele was amplified from plasmid pPM123 using primers vp2224 AA1-6/28-end w/o Stop & end vp2224 ccw. The PCR products were fused in a second PCR using primers vp2224 C-term PhoA-LacZ cw & end vp2224 ccw. The fusion product was digested with XbaI and HindIII and cloned in the corresponding site of plasmid pBAD33.

*Plasmids for genetic manipulation of S. putrefaciens and P. putida:* The desired DNA fragments were generated by PCR using appropriate primer pairs (see Supplementary file 1e) that in addition create overhangs suitable for subsequent Gibson assembly into EcoV-digested vector pNTPS138-R6K (Gibson et al., 2009; Lassak et al., 2010). If necessary, primer overhangs were also used to generate base substitutions in the gene fragment to be cloned. *Plasmids for Bacterial Two Hybrid (BACTH) analysis* were similarly generated by amplification of the desired DNA-fragments, which were then introduced into the suitable vectors by Gibson assembly.

### Soft-agar swimming assays

*V. parahaemolyticus* soft-agar swimming assays were essentially performed as described in Ringgaard et al., 2014 with the following modifications. Late exponential cultures of the required strains were used to prick LB plates with 0.3% agar, and incubated for 30 hr at 30 ° C. For *S. putrefaciens* and *P. putida* soft-agar swimming assays, 2 µl of an exponentially growing culture of the appropriate strains were spotted onto 0.25% LB agar plates and incubated at 30 °C (*S. putrefaciens*) or room temperature (*P. putida*) for about 18 hrs. Strains to be directly compared were always spotted onto the same plate. For the swimming assays in soft agar, strains of *V. parahaemolyticus* and *S. putrefaciens*, which are lacking lateral flagellin gene(s) (*ΔlafA; ΔflaAB_2_*) were used.

### Bacterial-two-hybrid experiments, BACTH

BTH101 cells were made competent with calcium chloride. 15 μL aliquots were spotted in a 96 well plate, to which 2.5 μL of the corresponding pUT18(C) and pK(N)T25 derivative plasmids were added. After 30 min on ice, a heat shock at 42 ° C was applied for 30 s. The transformed cells were allowed to recover for 1 hr, after which the were grown in selective LB broth for another 3 hr. The resulting cultures were spotted on plates containing kanamycin, ampicillin, IPTG (0.25 mM) and X-Gal (80 μg/mL). The plates were photographed after 48–72 hr at 30 ° C.

### Video tracking of swimming cells

Video tracking of swimming cells was performed essentially as described previously (Ringgaard et al., 2014). Swimming cells were recorded using the streaming acquisition function in the Metamorph software and the swimming paths of individual cells were tracked using the MTrackJ plug-in for ImageJ. The swimming speed, displacement, and number of reversals of individual cells were then measured and the average plotted with error bars indicating the standard deviation. Video tracking was performed using a Zeiss Axio Imager M1 fluorescence microscope. Images were collected with a Cascade:1 K CCD camera (Photometrics), using a Zeiss αPlan-Fluar 40 x/1.45 Oil phase contrast objective.

### Transmission-electron-microscopy (TEM) analysis

Cell cultures grown to an OD600=0.5–0.6 were spotted on a plasma-discharged carbon-coated copper grid (Plano, Cat#S162-3) and rinsed with 0.002% uranyl acetate. Afterwards they were rinsed with water and blotted dry with Whatman filter paper. TEM images were obtained with a JEOL JEM-1400 Plus 120 KV transmission electron microscope at 80 kV.

### Flagellum labeling

To fluorescently label flagellar filaments, maleimide-ligates dyes (Alexa Fluor 488 C5 maleimide fluorescent dye; Thermo Fisher Scientific) were coupled to surface-exposed cysteine residues, which were introduced into the flagellins (FlaA and FlaB) of the polar flagellar system in *S. putrefaciens* and *P. putida* as described (Kühn et al., 2017; Hintsche et al., 2017). Images were recorded as described in the microscopy section.

### Fluorescence microscopy

Florescence microscopy on *V. parahaemolyticus* was carried out essentially as previously described (Muraleedharan et al., 2018), using a Nikon eclipse Ti inverted Andor spinning-disc confocal microscope equipped with a 100 x lens, an Andor Zyla sCMOS cooled camera, and an Andor FRAPPA system. Fluorescence microscopy on *S. putrefaciens* and *P. putida* was carried out as previously described (Kühn et al., 2017) using a custom microscope set-up (Visitron Systems, Puchheim, Germany) based on a Leica DMI 6000 B inverse microscope (Leica) equipped with a pco.edge sCMOS camera (PCO), a SPECTRA light engine (lumencor), and an HCPL APO 63×/1.4–0.6 objective (Leica) using a custom filter set (T495lpxr, ET525/50 m; Chroma Technology) using the VisiView software (Visitron Systems, Puchheim, Germany). Microscopy images were analyzed using ImageJ imaging software (http://rsbweb.nih.gov/ij) and Metamorph Offline (version 7.7.5.0, Molecular Devices). DIC (*Vibrio*) and phase contrast (*Shewanella*, *Pseudomonas*) were used according to the preferred settings for the corresponding species in the labs. Demographs were generated as described by Cameron et al., 2014 modified in Heering and Ringgaard, 2016 and Heering et al., 2017.

### Mapping interaction partners using co-immunoaffinity purification and mass spectrometry (co-IP-MS)

For sample preparation of the IP-MS experiments we were using a modified version of the protocol presented by Turriziani et al., 2014. Cells were centrifuged and cell pellets were washed with cold PBS. Cell pellets were resuspended in lysis buffer (50 mM HEPES (pH 7.5), 150 mM NaCl, 0.5 % NP50, 5 mM EDTA, compete mini protease inhibitors (Complete Mini (Roche)). Cell lysis was performed by repetitive sonication. After removing cell debris by centrifugation 10 µl GFP-trap Sepharose (Chromotek) slurry was added to the lysate and incubation was carried out for 1.5 hr on a rotating shaker at 4 °C. Then the beads were pelleted, the supernatant removed and the beads washed 4 x with 100 mM NH_4_CO_3_ to remove remaining protease inhibitors and detergents. 200 µl elution buffer (1 M urea, 100 mM NH_4_CO_3_, 1 µg Trypsin (Promega)) was added to the beads and incubated at 1000 rpm on a thermomixer at 27 °C for 45 min. Beads were centrifuged and supernatant was collected. In order to increase peptide recovery 2 washing steps with 100 µL elution buffer 2 (1 M urea, 100 mM NH_4_CO_3_, 5 mM Tris(2-caboxyethyl)phosphine)) was performed and the individual bead supernatants were collected into one tube. Tryptic digestion was carried out overnight at 30 °C. After digest alkylation was performed with 10 mM iodoacetamide at 25 °C (in the dark). Then the samples were acidified (1% trifluoroacetic acid [TFA]) and C18 Microspin column (Harvard Apparatus) was carried out according to the manufacturer´s instruction. The samples were dried and recovered in 0.1% TFA and applied to liquid chromatography-mass spectrometry (LC-MS) analysis.

LC-MS analysis of digested lysates was performed on a Thermo QExactive Plus mass spectrometer (Thermo Scientific), which was connected to an electrospray ionsource (Thermo Scientific). Peptide separation was carried out using an Ultimate 3000 RSLCnano with Proflow upgrade (Thermo Scientific) equipped with a RP-HPLC column (75 μm x 42 cm) packed in-house with C18 resin (2.4 μm; Dr. Maisch) on an in-house designed column heater. The following separating gradient was used: 98% solvent A (0.15% formic acid) and 2% solvent B (99.85% acetonitrile, 0.15% formic acid) to 35% solvent B over 90 at a flow rate of 300 nl/min. The data acquisition mode was set to obtain one high resolution MS scan at a resolution of 70,000 full width at half maximum (at m/z 200) followed by MS/MS scans of the 10 most intense ions. To increase the efficiency of MS/MS attempts, the charged state screening modus was enabled to exclude unassigned and singly charged ions. The dynamic exclusion duration was set to 30 s. The ion accumulation time was set to 50ms (MS) and 50ms at 17,500 resolution (MS/MS). The automatic gain control (AGC) was set to 3x10^6^ for MS survey scan and 1x10^5^ for MS/MS scans.

MS raw data was then analyzed with MaxQuant (Version 1.6.3.4; https://www.nature.com/articles/nbt.1511) using a *V.parahaemolyticus* RIMD 2210633 uniprot database (https://www.uniprot.org/). MaxQuant was executed in standard settings with activated ‘match between runs’ option. The search criteria were set as follows: full tryptic specificity was required (cleavage after lysine or arginine residues); two missed cleavages were allowed; carbamidomethylation (C) was set as fixed modification; oxidation (M) and deamidation (N,Q) as variable modification. For further data analysis the MaxQuant LFQ values were loaded into Perseus (https://www.nature.com/articles/nmeth.3901) and a Student’s T-test was performed on LFQ values with false discovery rate 0.01 and S0: 0.1 as significance cut-off. The mass spectrometry proteomics data have been deposited to the ProteomeXchange Consortium via the PRIDE (PubMed ID: 34723319) partner repository with the dataset identifier PXD045379 (http://www.ebi.ac.uk/pride).

### Membrane topology mapping of FipA

We experimentally determined the membrane orientation of FipA in the membrane using the dual pho-lac reporter system (Karimova et al., 2009), which consists of a translational fusion of the *E. coli* alkaline phosphatase fragment PhoA22-472 and the α-peptide of *E. coli* β-galactosidase, LacZ4-60. A periplasmic localization of the reporter leads to high alkaline phosphatase activity and low β-galactosidase activity, whereas a cytosolic location of the reporter results in high β-galactosidase activity and low alkaline phosphatase activity. Pho-Lac-FipA and FipA-Pho-Lac fusion proteins were ectopically expressed in *E. coli* DH5α grown on a dual-indicator LB medium containing a blue indicator for phosphatase activity (X-Phos) and red indicator for β-galactosidase activity (Red-Gal) (see Figure 1—figure supplement 1).

### Bioinformatic analysis

Homologues of FipA were searched using BLAST against the KEGG database, with the sequence of the *V. parahaemolyticus* protein. All homologues containing a DUF2802 were included. The search was later expanded to species known to encode a FlhF homologue (defined as the highest scoring result from a BLAST search using FlhF of *V. parahaemolyticus* as a query, that was also encoded upstream of an FlhG homologue). The flagellation phenotype was later corroborated in the description registered at the List of Prokaryotic names with Standing in Nomenclature (Parte et al., 2020).

## Data Availability

All data generated or analysed during this study are included in the manuscript and supporting files.

## Additional files

Supplementary file 1. Supplementary tables. (**a**) Enriched proteins in Co-IP FlhF-sfGFP vs. sfGFP. The genes with the largest enrichment in FlhF-sfGFP vs. sfGFP are shown. The corresponding protein designation is indicated if available. (**b**) Flagellation pattern and presence of FipA and FlhF in bacteria. (**c**) Bacterial strains used in this study. (**d**) Plasmids used in this study. (**e**) Oligonucleotides used in this study.

MDAR checklist

Source data 1. Contains the data used for generation of the indicated figure panels.

## Appendix 1

**Appendix 1—key resources table:**

Reagent type (species) or resource	Designation	Source or reference	Identifiers	Additional information
Gene (Vibrio parahaemolyticus)	*fipA*	KEGG	VP2224	
Gene (Pseudomonas putida)	*fipA*	KEGG	PP_4331	
Gene (Shewanella putrefaciens)	*fipA*	KEGG	Sputcn32_2550	
Gene (Vibrio parahaemolyticus)	*flhF*	KEGG	VP2234	
Gene (Pseudomonas putida)	*flhF*	KEGG	PP_4343	
Gene (Shewanella putrefaciens)	*flhF*	KEGG	Sputcn32_2561	
Strain (*Escherichia coli*)	SM10λpir	Simon et al., 1983		
Strain (*Escherichia coli*)	DH5pir	Miller and Mekalanos, 1988		
Strain (*Escherichia coli*)	BTH101	Euromedex		
Strain (*Escherichia coli*)	WM3064	Metcalf, University of Illinois, Urbana‐Champaign		
Strain (Vibrio parahaemolyticus)	RIMD 2210633	Makino et al., 2003		Wild type
Strain (Vibrio parahaemolyticus)	*Δvp2234 (ΔflhF), Δvpa1548 (ΔlafA*)	Arroyo-Pérez and Ringgaard, 2021	EP12	
Strain (Vibrio parahaemolyticus)	*Δvp2225 (ΔcheW*)	Ringgaard et al., 2014	SR58	
Strain (Vibrio parahaemolyticus)	*Δvpa1548 (ΔlafA*)	Arroyo-Pérez and Ringgaard, 2021	JH2	
Strain (Vibrio parahaemolyticus)	*Δvp2224 (ΔfipA), Δvpa1548 (ΔlafA*)	this study	EP15	Materials and Methods
Strain (Vibrio parahaemolyticus)	*Δvp2234 (ΔflhF*)	Arroyo-Pérez and Ringgaard, 2021	PM60	
Strain (Vibrio parahaemolyticus)	*Δvp2224 (ΔfipA*)	this study	SW01	Materials and Methods
Strain (Vibrio parahaemolyticus)	*Δvp2191 (ΔhubP*)	Arroyo-Pérez and Ringgaard, 2021	JH4	
Strain (Vibrio parahaemolyticus)	*Δvp2234::vp2234-sfgfp (ΔflhF::flhF-sfgfp*)	Arroyo-Pérez and Ringgaard, 2021	PM69	
Strain (Vibrio parahaemolyticus)	*Δvp2234::vp2234-sfgfp (ΔflhF::flhF-sfgfp), Δvp2224 (fipA*)	this study	PM77	Materials and Methods
Strain (Vibrio parahaemolyticus)	*Δvp2234::vp2234-sfgfp (ΔflhF::flhF-sfgfp), Δvp2191 (hubP*)	Arroyo-Pérez and Ringgaard, 2021	EP11	
Strain (Vibrio parahaemolyticus)	*Δvp2234::vp2234-sfgfp (ΔflhF::flhF-sfgfp), Δvp2224 (fipA), Δvp2191 (hubP*)	this study	EP09	Materials and Methods
Strain (Vibrio parahaemolyticus)	*vp2224 (fipA) L129A*	this study	PM65	Materials and Methods
Strain (Vibrio parahaemolyticus)	*vp2224 (fipA) G110A*	this study	PM66	Materials and Methods
Strain (Vibrio parahaemolyticus)	*Δvp2234::vp2234-sfgfp (ΔflhF::flhF-sfgfp), vp2224 (fipA) L129A*	this study	EP16	Materials and Methods
Strain (Vibrio parahaemolyticus)	*Δvp2234::vp2234-sfgfp (ΔflhF::flhF-sfgfp), vp2224 (fipA) G110A*	this study	EP17	Materials and Methods
Strain (Vibrio parahaemolyticus)	*vp2224 L129A, Δvpa1548(lafA*)	this study	EP13	Materials and Methods
Strain (Vibrio parahaemolyticus)	*vp2224 G110A, Δvpa1548(lafA*)	this study	EP14	Materials and Methods
Strain (Vibrio parahaemolyticus)	*Δvp2224::vp2224-sfgfp (ΔfipA::fipA-sfgfp*)	this study	PM64	Materials and Methods
Strain (Vibrio parahaemolyticus)	*Δvp2224::vp2224-sfgfp (ΔfipA::fipA-sfgfp), Δvp2234 (flhF*)	this study	PM68	Materials and Methods
Strain (Vibrio parahaemolyticus)	Δvp2224::vp2224 L129A-sfgfp (ΔfipA L129A::fipA-sfgfp)	this study	PM71	Materials and Methods
Strain (Vibrio parahaemolyticus)	Δvp2224::vp2224 G110A-sfgfp (ΔfipA G110A::fipA-sfgfp)	this study	PM72	Materials and Methods
Strain (Pseudomonas putida)	KT2440	Nelson et al., 2002		Wild type
Strain (Pseudomonas putida)	FliC S267C	Hintsche et al., 2017		
Strain (Pseudomonas putida)	FliC S267C ΔflhF	this study		Materials and Methods
Strain (Pseudomonas putida)	ΔfipA	this study		Materials and Methods
Strain (Pseudomonas putida)	FliC S267C ΔfipA	this study		Materials and Methods
Strain (Pseudomonas putida)	FipA -DILEL-sfGFP	this study		Materials and Methods
Strain (Pseudomonas putida)	FliC S267C ΔflhF FipA-DILEL-sfGFP	this study		Materials and Methods
Strain (Pseudomonas putida)	FlhF-GS-mCherry	this study		Materials and Methods
Strain (Pseudomonas putida)	FliC S267C ΔfimV FipA -DILEL-sfGFP	this study		Materials and Methods
Strain (Pseudomonas putida)	ΔfipA FlhF-GS-mCherry	this study		Materials and Methods
Strain (Pseudomonas putida)	FliC S267C FipA G104A	this study		Materials and Methods
Strain (Pseudomonas putida)	FipA L123A	this study		Materials and Methods
Strain (Pseudomonas putida)	FliC S267C FipA L123A	this study		Materials and Methods
Strain (Pseudomonas putida)	FliC S267C FipA-DILEL-sfGFP	this study		Materials and Methods
Strain (Pseudomonas putida)	FliC S267C FipA L116A	this study		Materials and Methods
Strain (Pseudomonas putida)	FliC S267C FipA G104A-DILEL-sfGFP	this study		Materials and Methods
Strain (Pseudomonas putida)	FliC S267C FipA L123A-DILEL-sfGFP	this study		Materials and Methods
Strain (Pseudomonas putida)	FliC S267C FipA L116A-DILEL-sfGFP	this study		Materials and Methods
Strain (Pseudomonas putida)	FlhF-GS-mCherry ΔfimV	this study		Materials and Methods
Strain (Pseudomonas putida)	FliC S267C ΔfipA FipA KI	this study		Materials and Methods
Strain (Pseudomonas putida)	FlhF-GS-mCherry ΔfimV ΔfipA	this study		Materials and Methods
Strain (Pseudomonas putida)	FlhF-GS-mCherry FipA L123A	this study		Materials and Methods
Strain (Pseudomonas putida)	FlhF-GS-mCherry FipA L116A	this study		Materials and Methods
Strain (Pseudomonas putida)	FlhF-GS-mCherry FipA G104A	this study		Materials and Methods
Strain (Pseudomonas putida)	FliC S267C FlhF D362A-GS-mCherry	this study		Materials and Methods
Strain (Pseudomonas putida)	FliC S267C FlhF-GS-mCherry FipA ΔTMD	this study		Materials and Methods
Strain (Pseudomonas putida)	FliC S267C FlhF-GS-mCherry FipA ΔTMD	this study		Materials and Methods
Strain (Pseudomonas putida)	FliC S267C FipA ΔTMD-DILEL-sfGFP	this study		Materials and Methods
Strain (Shewanella putrefaciens)	CN-32	Fredrickson et al., 1998		Wild type
Strain (Shewanella putrefaciens)	ΔflhF	Rossmann et al., 2015		
Strain (Shewanella putrefaciens)	CheA-mCherry	this study		Materials and Methods
Strain (Shewanella putrefaciens)	flgE1 T183C	Rossmann et al., 2019		
Strain (Shewanella putrefaciens)	flaB1 T166C flaA1 T174C ΔflagL	Kühn et al., 2017		
Strain (Shewanella putrefaciens)	ΔfipA	this study		Materials and Methods
Strain (Shewanella putrefaciens)	CheA-mCherry ΔfipA	this study		Materials and Methods
Strain (Shewanella putrefaciens)	flaB1 T166C flaA1 T174C ΔflagL ΔfipA	this study		Materials and Methods
Strain (Shewanella putrefaciens)	FlhF-GS-mVenus	this study		Materials and Methods
Strain (Shewanella putrefaciens)	FipA-DILEL-sfGFP	this study		Materials and Methods
Strain (Shewanella putrefaciens)	FlhF-GS-mVenus ΔfipA	this study		Materials and Methods
Strain (Shewanella putrefaciens)	FlhF-GS-mVenus ΔflhG	this study		Materials and Methods
Strain (Shewanella putrefaciens)	FipA-DILEL-sfGFP ΔflhF	this study		Materials and Methods
Strain (Shewanella putrefaciens)	FlhF-GS-mVenus ΔhubP	this study		Materials and Methods
Strain (Shewanella putrefaciens)	FlhF-GS-mVenus ΔfipA ΔhubP	this study		Materials and Methods
Strain (Shewanella putrefaciens)	flgE1 T183C ΔflagL	Hook et al., 2020		
Strain (Shewanella putrefaciens)	FipA-DILEL-sfGFP ΔhubP	this study		Materials and Methods
Strain (Shewanella putrefaciens)	HubP-mCherry FlhF-GS-Venus	this study		Materials and Methods
Strain (Shewanella putrefaciens)	FlhF-GS-mVenus ΔhubP ΔSputcn32_3157	this study		Materials and Methods
Strain (Shewanella putrefaciens)	flaB1 T166C flaA1 T174C ΔflagL FipA-DILEL-sfGFP	this study		Materials and Methods
Strain (Shewanella putrefaciens)	flaB1 T166C flaA1 T174C ΔflagL ΔflhF	this study		Materials and Methods
Strain (Shewanella putrefaciens)	ΔfipA fipA (Sputcn32_2550) KI	this study		Materials and Methods
Strain (Shewanella putrefaciens)	FipA L118A	this study		Materials and Methods
Strain (Shewanella putrefaciens)	FipA G106A	this study		Materials and Methods
Strain (Shewanella putrefaciens)	FipA L118-DILEL-sfGFP	this study		Materials and Methods
Strain (Shewanella putrefaciens)	FipA G106A-DILEL-sfGFP	this study		Materials and Methods
Strain (Shewanella putrefaciens)	flgE1 T183C ΔflagL FliM1-GS-sfGFP	Hook et al., 2020		
Strain (Shewanella putrefaciens)	FipA L125A	this study		Materials and Methods
Strain (Shewanella putrefaciens)	flaB1 T166C flaA1 T174C ΔflagL ΔfipA ΔhubP	this study		Materials and Methods
Strain (Shewanella putrefaciens)	FipA L125A-DILEL-sfGFP	this study		Materials and Methods
Strain (Shewanella putrefaciens)	FlhF-mCherry FipA-DILEL-sfGFP	this study		Materials and Methods
Strain (Shewanella putrefaciens)	FipA L118A FlhF-GS-mVenus	this study		Materials and Methods
Strain (Shewanella putrefaciens)	FipA G106A FlhF-GS-mVenus	this study		Materials and Methods
Strain (Shewanella putrefaciens)	FipA L125A FlhF-GS-mVenus	this study		Materials and Methods
Strain (Shewanella putrefaciens)	FipA ΔTMD	this study		Materials and Methods
Strain (Shewanella putrefaciens)	FlhF-GS-mVenus FipA ΔTMD	this study		Materials and Methods
Strain (Shewanella putrefaciens)	FipA ΔTMD-DILEL-sfGFP	this study		Materials and Methods
Antibody	JL-8 anti-GFP (mouse monoclonal)	Takarabio	632380	WB (1:10,000)
Antibody	Amersham ECL mouse IgG (sheep, polyclonal)	General Electric	NA931	WB (1:5,000)
Antibody	Anti-GFP (mouse)	Roche	11814460001	WB (1:5,000)
Antibody	Anti-mCherry (rabbit)	Biovision	5993–100	WB (1:10,000)
Antibody	Anti-mouse	Sigma	A3562	WB (1:5,000)
Antibody	Anti-rabbit	Sigma	A8025	WB (1:20,000)
Recombinant DNA reagent	pDM4	Milton et al., 1996		Suicide vector for gene deletions in Vibrio sp.
Recombinant DNA reagent	pJH036	Iyer et al., 2020		pBAD33 derivative for sfGFP C-terminal fusion
Recombinant DNA reagent	pNPTS138-R6KT	Lassak et al., 2010		Suicide vector for gene deletions in P. putida and S. Putrefaciens
Recombinant DNA reagent	pKT25	Karimova et al., 1998		For bacterial two hybrid assay
Recombinant DNA reagent	pKNT25	Karimova et al., 1998		For bacterial two hybrid assay
Recombinant DNA reagent	pUT18	Karimova et al., 1998		For bacterial two hybrid assay
Recombinant DNA reagent	pUT18C	Karimova et al., 1998		For bacterial two hybrid assay
Recombinant DNA reagent	pJH003	Heering and Ringgaard, 2016		For deletion of vpa1548(lafA)
Recombinant DNA reagent	pSW022	This work		For deletion of vp2224(fipA)
Recombinant DNA reagent	pPM188fip	Arroyo-Pérez and Ringgaard, 2021		For insertion of vp2234- sfgfp (flhF-sfgfp), replacing native flhF
Recombinant DNA reagent	pPM178	this work		For insertion of vp2224- sfgfp (fipA-sfgfp), replacing native fipA
Recombinant DNA reagent	pPM179	this work		For insertion of vp2224 (fipA) G110A point mutation in the chromosome in the native locus
Recombinant DNA reagent	pPM180	this work		For insertion of vp2224 (fipA) L129A point mutation in the chromosome in the native locus
Recombinant DNA reagent	pPM191	this work		For insertion of vp2224 (fipA) G110A fused to sfGFP in the chromosome in the native locus
Recombinant DNA reagent	pPM187	this work		For insertion of vp2224 (fipA) L129A fused to sfGFP in the chromosome in the native locus
Recombinant DNA reagent	pPM039	Arroyo-Pérez and Ringgaard, 2021		For deletion of vp2191 (hubP)
Recombinant DNA reagent	pPM194	this work		For overexpression of VP2224(FipA) Δ7–27 -sfGFP
Recombinant DNA reagent	pPM146	this work		For overexpression of VP2224(FipA)
Recombinant DNA reagent	pPM159	this work		For overexpression of VP2224(FipA)-sfGFP
Recombinant DNA reagent	pNPTS138-R6KT flhF KO (PP_4343)	this study		For deletion of the flhF gene (PP_4343) in P. putida KT2440; Kanr
Recombinant DNA reagent	pNPTS138-R6KT FlhF K235A (PP_4343)	this study		For in frame complementation of flhF (PP_4343) with FlhF K235A mutant in P. putida KT2440; Kanr
Recombinant DNA reagent	pNPTS138-R6KT FlhF-GS-mCherry (PP_4343)	this study		For in frame complementation of flhF (PP_4343) with FlhF-GS-mCherry in P. putida KT2440; Kanr
Recombinant DNA reagent	pNPTS138-R6KT FlhF K235A-GS-mCherry (PP_4343)	this study		For in frame complementation of flhF (PP_4343) with FlhF K235A-GS-mCherry mutant in P. putida KT2440; Kanr
Recombinant DNA reagent	pNPTS138-R6KT FlhF D301A-GS-mCherry (PP_4343)	this study		For in frame complementation of flhF (PP_4343) with FlhF D301A-GS-mCherry mutant in P. putida KT2440; Kanr
Recombinant DNA reagent	pNPTS138-R6KT FlhF D362A-GS-mCherry (PP_4343)	this study		For in frame complementation of flhF (PP_4343) with FlhF D362A-GS-mCherry mutant in P. putida KT2440; Kanr
Recombinant DNA reagent	pNPTS138-R6KT fipA KO (PP_4331)	this study		For deletion of the fipA gene (PP_4331) in P. putida KT2440; Kanr
Recombinant DNA reagent	pNPTS138-R6KT fipA KI (PP_4331)	this study		For in frame complementation of fipA (PP_4331) with wild type fipA in P. putida KT2440; Kanr
Recombinant DNA reagent	pNPTS138-R6KT FipA ΔTMD (AS5-22) (PP_4331)	this study		For in frame complementation of fipA (PP_4331) with FipA ΔTMD mutant in P. putida KT2440; Kanr
Recombinant DNA reagent	pNPTS138-R6KT FipA G104A (PP_4331)	this study		For in frame complementation of fipA (PP_4331) with FipA G104A mutant in P. putida KT2440; Kanr
Recombinant DNA reagent	pNPTS138-R6KT FipA L116A (PP_4331)	this study		For in frame complementation of fipA (PP_4331) with FipA L116A mutant in P. putida KT2440; Kanr
Recombinant DNA reagent	pNPTS138-R6KT FipA L123A (PP_4331)	this study		For in frame complementation of fipA (PP_4331) with FipA L123A mutant in P. putida KT2440; Kanr
Recombinant DNA reagent	pNPTS138-R6KT FipA-DILEL-sfGFP (PP_4331)	this study		For in frame complementation of fipA (PP_4331) with FipA-DILEL-sfGFP in P. putida KT2440; Kanr
Recombinant DNA reagent	pNPTS138-R6KT FipA ΔTMD-DILEL-sfGFP (AS5-22) (PP_4331)	this study		For in frame complementation of fipA (PP_4331) with FipA ΔTMD-DILEL-sfGFP mutant in P. putida KT2440; Kanr
Recombinant DNA reagent	pNPTS138-R6KT FipA G104A-DILEL-sfGFP (PP_4331)	this study		For in frame complementation of fipA (PP_4331) with FipA G104A-DILEL-sfGFP mutant in P. putida KT2440; Kanr
Recombinant DNA reagent	pNPTS138-R6KT FipA L116A-DILEL-sfGFP (PP_4331)	this study		For in frame complementation of fipA (PP_4331) with FipA L116A-DILEL-sfGFP mutant in P. putida KT2440; Kanr
Recombinant DNA reagent	pNPTS138-R6KT FipA L123A-DILEL-sfGFP (PP_4331)	this study		For in frame complementation of fipA (PP_4331) with FipA L123A-DILEL-sfGFP mutant in P. putida KT2440; Kanr
Recombinant DNA reagent	pNPTS138-R6KT polar flagellar cluster KO (Sputcn32_2548–2608)	this study		plasmid for deletion of the polar flagellar gene cluster (Sputcn32_2548–2608) in S. putrefaciens CN-32; Kanr
Recombinant DNA reagent	pNPTS138-R6KT lateral flagellar cluster KO (Sputcn32_3444–3485)	Lassak et al., 2010		plasmid for deletion of the lateral flagellar gene cluster (Sputcn32_3444–3485) in S. putrefaciens CN-32; Kanr
Recombinant DNA reagent	pNPTS138-R6KT flagL KO (Sputcn32_3455, Sputcn32_3456)	Rossmann et al., 2015		plasmid for deletion of the lateral flagellin genes (Sputcn32_3455, Sputcn32_3456) in S. putrefaciens CN-32; Kanr
Recombinant DNA reagent	pNPTS138-R6KT hubP KO (Sputcn32_2442)	Rossmann et al., 2015		plasmid for deletion of the hubP gene (Sputcn32_2442) in S. putrefaciens CN-32; Kanr
Recombinant DNA reagent	pNPTS138-R6KT flhF KO (Sputcn32_2561)	Rossmann et al., 2015		plasmid for deletion of the flhF gene (Sputcn32_2561) in S. putrefaciens CN-32; Kanr
Recombinant DNA reagent	pNPTS138-R6KT flhG KO (Sputcn32_2560)	Schuhmacher et al., 2015a		plasmid for deletion of the flhG gene (Sputcn32_2560) in S. putrefaciens CN-32; Kanr
Recombinant DNA reagent	pNPTS138-R6KT FlhF-GS-Venus (Sputcn32_2561)	this study		plasmid for in frame complementation of flhF (Sputcn32_2561) with FlhF-GS-mVenus in S. putrefaciens CN-32; Kanr
Recombinant DNA reagent	pNPTS138-R6KT fipA KO (Sputcn32_2550)	this study		plasmid for deletion of the fipA gene (Sputcn32_2550) in S. putrefaciens CN-32; Kanr
Recombinant DNA reagent	pNPTS138-R6KT fipA KI (Sputcn32_2550)	this study		plasmid for in frame complementation of fipA (Sputcn32_2550) with wild type fipA in S. putrefaciens CN-32; Kanr
Recombinant DNA reagent	pNPTS138-R6KT FipA ΔTMD (AS5-23) (Sputcn32_2550)	this study		plasmid for in frame complementation of fipA (Sputcn32_2550) with FipA ΔTMD mutant in S. putrefaciens CN-32; Kanr
Recombinant DNA reagent	pNPTS138-R6KT FipA G106A (Sputcn32_2550)	this study		plasmid for in frame complementation of fipA (Sputcn32_2550) with FipA G106A mutant in S. putrefaciens CN-32; Kanr
Recombinant DNA reagent	pNPTS138-R6KT FipA L118A (Sputcn32_2550)	this study		plasmid for in frame complementation of fipA (Sputcn32_2550) with FipA L118A mutant in *S. putrefaciens* CN-32; Kanr
Recombinant DNA reagent	pNPTS138-R6KT FipA L125A (Sputcn32_2550)	this study		plasmid for in frame complementation of fipA (Sputcn32_2550) with FipA L125 mutant in *S. putrefaciens* CN-32; Kanr
Recombinant DNA reagent	pNPTS138-R6KT FipA-DILEL-sfGFP (Sputcn32_2550)	this study		plasmid for in frame complementation of fipA (Sputcn32_2550) with FipA-DILEL-sfGFP in *S. putrefaciens* CN-32; Kanr
Recombinant DNA reagent	pNPTS138-R6KT FipA ΔTMD-DILEL-sfGFP (AS5-23) (Sputcn32_2550)	this study		plasmid for in frame complementation of fipA (Sputcn32_2550) with FipA ΔTMD-DILEL-sfGFP mutant in *S. putrefaciens* CN-32; Kanr
Recombinant DNA reagent	pNPTS138-R6KT FipA G106A-DILEL-sfGFP (Sputcn32_2550)	this study		plasmid for in frame complementation of fipA (Sputcn32_2550) with FipA G106A-DILEL-sfGFP mutant in *S. putrefaciens* CN-32; Kanr
Recombinant DNA reagent	pNPTS138-R6KT FipA L116A-DILEL-sfGFP (Sputcn32_2550)	this study		plasmid for in frame complementation of fipA (Sputcn32_2550) with FipA L116A-DILEL-sfGFP mutant in *S. putrefaciens* CN-32; Kanr
Recombinant DNA reagent	pNPTS138-R6KT FipA L125A-DILEL-sfGFP (Sputcn32_2550)	this study		plasmid for in frame complementation of fipA (Sputcn32_2550) with FipA L125A mutant in *S. putrefaciens* CN-32; Kanr
Recombinant DNA reagent	pNPTS138-R6KT FliM1-GS-sfGFP (Sputcn32_2569)	Hook et al., 2020		plasmid for in frame complementation of fliM1 (Sputcn32_2569) with FliM1-GS-sfGFP in *S. putrefaciens* CN-32; Kanr
Recombinant DNA reagent	pSW74	this study		T25-vp2224(fipA)Δ1–27
Recombinant DNA reagent	pSW119	this study		T18-vp2224(fipA)Δ1–27
Recombinant DNA reagent	pPM118	this study		vp2224(fipA)Δ1–27 T18
Recombinant DNA reagent	pPM119	this study		vp2224(fipA)Δ1–27 T25
Recombinant DNA reagent	pPM124	this study		vp2234(flhF)-T18
Recombinant DNA reagent	pPM128	this study		vp2234(flhF)-T25
Recombinant DNA reagent	pPM132	this study		T18-vp2234(flhF)
Recombinant DNA reagent	pPM136	this study		T25-vp2234(flhF)
Recombinant DNA reagent	pPM160	this study		T18-vp2224(fipA) Δ1–27 G110A
Recombinant DNA reagent	pPM161	this study		T18-vp2224(fipA) Δ1–27 E126A
Recombinant DNA reagent	pPM162	this study		T18-vp2224(fipA) Δ1–27 L129A
Recombinant DNA reagent	pKT25 FlhF (PP_4343)	this study		plasmid for BACTH assay carrying T25-FlhF (PP_4343); Kanr
Recombinant DNA reagent	pKNT25 FlhF (PP_4343)	this study		plasmid for BACTH assay carrying FlhF-T25 (PP_4343); Kanr
Recombinant DNA reagent	pUT18 FlhF (PP_4343)	this study		plasmid for BACTH assay carrying FlhF-T18 (PP_4343); Ampr
Recombinant DNA reagent	pUT18C FlhF (PP_4343)	this study		plasmid for BACTH assay carrying T18-FlhF (PP_4343); Ampr
Recombinant DNA reagent	pKT25 FlhF K235A (PP_4343)	this study		plasmid for BACTH assay carrying T25-FlhF K235A (PP_4343); Kanr
Recombinant DNA reagent	pKNT25 FlhF K235A (PP_4343)	this study		plasmid for BACTH assay carrying FlhF K235A -T25 (PP_4343); Kanr
Recombinant DNA reagent	pUT18 FlhF K235A (PP_4343)	this study		plasmid for BACTH assay carrying FlhF K235A -T18 (PP_4343); Ampr
Recombinant DNA reagent	pUT18C FlhF K235A (PP_4343)	this study		plasmid for BACTH assay carrying T18-FlhF K235A (PP_4343); Ampr
Recombinant DNA reagent	pKT25 FipA (PP_4331)	this study		plasmid for BACTH assay carrying T25-FipA (PP_4331); Kanr
Recombinant DNA reagent	pKNT25 FipA (PP_4331)	this study		plasmid for BACTH assay carrying FipA-T25 (PP_4331); Kanr
Recombinant DNA reagent	pUT18 FipA (PP_4331)	this study		plasmid for BACTH assay carrying FipA-T18 (PP_4331); Ampr
Recombinant DNA reagent	pUT18C FipA (PP_4331)	this study		plasmid for BACTH assay carrying T18-FipA (PP_4331); Ampr
Recombinant DNA reagent	pKT25 FipA G104A (PP_4331)	this study		plasmid for BACTH assay carrying T25-FipA G104A (PP_4331); Kanr
Recombinant DNA reagent	pKNT25 FipA G104A (PP_4331)	this study		plasmid for BACTH assay carrying FipA G104A -T25 (PP_4331); Kanr
Recombinant DNA reagent	pUT18 FipA G104A (PP_4331)	this study		plasmid for BACTH assay carrying FipA G104A -T18 (PP_4331); Ampr
Recombinant DNA reagent	pUT18C FipA G104A (PP_4331)	this study		plasmid for BACTH assay carrying T18-FipA G104A (PP_4331); Ampr
Recombinant DNA reagent	pKT25 FipA L116A (PP_4331)	this study		plasmid for BACTH assay carrying T25-FipA L116A (PP_4331); Kanr
Recombinant DNA reagent	pKNT25 FipA L116A (PP_4331)	this study		plasmid for BACTH assay carrying FipA L116A -T25 (PP_4331); Kanr
Recombinant DNA reagent	pUT18 FipA L116A (PP_4331)	this study		plasmid for BACTH assay carrying FipA L116A -T18 (PP_4331); Ampr
Recombinant DNA reagent	pUT18C FipA L116A (PP_4331)	this study		plasmid for BACTH assay carrying T18-FipA L116A (PP_4331); Ampr
Recombinant DNA reagent	pKT25 FipA L125A (PP_4331)	this study		plasmid for BACTH assay carrying T25-FipA L123A (PP_4331); Kanr
Recombinant DNA reagent	pKNT25 FipA L125A (PP_4331)	this study		plasmid for BACTH assay carrying FipA L123A -T25 (PP_4331); Kanr
Recombinant DNA reagent	pUT18 FipA L125A (PP_4331)	this study		plasmid for BACTH assay carrying FipA L123A -T18 (PP_4331); Ampr
Recombinant DNA reagent	pUT18C FipA L125A (PP_4331)	this study		plasmid for BACTH assay carrying T18-FipA L123A (PP_4331); Ampr
Recombinant DNA reagent	pKT25 FlhF (Sputcn32_2561)	this study		plasmid for BACTH assay carrying T25-FlhF (Sputcn32_2561); Kanr
Recombinant DNA reagent	pKNT25 FlhF (Sputcn32_2561)	this study		plasmid for BACTH assay carrying FlhF-T25 (Sputcn32_2561); Kanr
Recombinant DNA reagent	pUT18 FlhF (Sputcn32_2561)	this study		plasmid for BACTH assay carrying FlhF-T18 (Sputcn32_2561); Ampr
Recombinant DNA reagent	pUT18C FlhF (Sputcn32_2561)	this study		plasmid for BACTH assay carrying T18-FlhF (Sputcn32_2561); Ampr
Recombinant DNA reagent	pKT25 FipA (Sputcn32_2550)	this study		plasmid for BACTH assay carrying T25-FipA (Sputcn32_2550); Kanr
Recombinant DNA reagent	pKNT25 FipA (Sputcn32_2550)	this study		plasmid for BACTH assay carrying FipA-T25 (Sputcn32_2550); Kanr
Recombinant DNA reagent	pUT18 FipA (Sputcn32_2550)	this study		plasmid for BACTH assay carrying FipA-T18 (Sputcn32_2550); Ampr
Recombinant DNA reagent	pUT18C FipA (Sputcn32_2550)	this study		plasmid for BACTH assay carrying T18-FipA (Sputcn32_2550); Ampr
Recombinant DNA reagent	pKT25 FipA G106A (Sputcn32_2550)	this study		plasmid for BACTH assay carrying T25-FipA G106A (Sputcn32_2550); Kanr
Recombinant DNA reagent	pKNT25 FipA G106A (Sputcn32_2550)	this study		plasmid for BACTH assay carrying FipA G106A -T25 (Sputcn32_2550); Kanr
Recombinant DNA reagent	pUT18 FipA G106A (Sputcn32_2550)	this study		plasmid for BACTH assay carrying FipA G106A -T18 (Sputcn32_2550); Ampr
Recombinant DNA reagent	pUT18C FipA G106A (Sputcn32_2550)	this study		plasmid for BACTH assay carrying T18-FipA G106A (Sputcn32_2550); Ampr
Recombinant DNA reagent	pKT25 FipA L116A (Sputcn32_2550)	this study		plasmid for BACTH assay carrying T25-FipA L116A (Sputcn32_2550); Kanr
Recombinant DNA reagent	pKNT25 FipA L116A (Sputcn32_2550)	this study		plasmid for BACTH assay carrying FipA L116A -T25 (Sputcn32_2550); Kanr
Recombinant DNA reagent	pUT18 FipA L116A (Sputcn32_2550)	this study		plasmid for BACTH assay carrying FipA L116A -T18 (Sputcn32_2550); Ampr
Recombinant DNA reagent	pUT18C FipA L116A (Sputcn32_2550)	this study		plasmid for BACTH assay carrying T18-FipA L116A (Sputcn32_2550); Ampr
Recombinant DNA reagent	pKT25 FipA L125A (Sputcn32_2550)	this study		plasmid for BACTH assay carrying T25-FipA L125A (Sputcn32_2550); Kanr
Recombinant DNA reagent	pKNT25 FipA L125A (Sputcn32_2550)	this study		plasmid for BACTH assay carrying FipA L125A -T25 (Sputcn32_2550); Kanr
Recombinant DNA reagent	pUT18 FipA L125A (Sputcn32_2550)	this study		plasmid for BACTH assay carrying FipA L125A -T18 (Sputcn32_2550); Ampr
Recombinant DNA reagent	pUT18C FipA L125A (Sputcn32_2550)	this study		plasmid for BACTH assay carrying T18-FipA L125A (Sputcn32_2550); Ampr
Sequence-based reagent	VP2224-del-a	this study	PCR primers	CCCCC tctaga ACGTTGTCATGCTTGGTGAAAGCA
Sequence-based reagent	VP2224-del-b	this study	PCR primers	AGTCTCTTCAGCCATCGTCATTC
Sequence-based reagent	VP2224-del-c	this study	PCR primers	gaatgacgatggctgaagagact cgacgataaagagaataaaaagaagc
Sequence-based reagent	VP2224-del-d	this study	PCR primers	CCCCC tctaga ACGCGACGCTGCTGACCCGCAGAA
Sequence-based reagent	VP2224-check	this study	PCR primers	acaaactccgtggggatgaatac
Sequence-based reagent	vp2224 AA1-6/28-end w/o Stop	this study	PCR primers	ccccc ctcaga atg gctgaagagacttttctgcgc
Sequence-based reagent	pUT18C/pKT25-vp2234-cw	this study	PCR primers	ccccc tctaga G aaaataaagcgattttttgccaaagac
Sequence-based reagent	pUT18C/pKT25-vp2234-ccw	this study	PCR primers	ccccc ggtacc ctagagtccttcgttgtcactg
Sequence-based reagent	vpa1548-del-d	this study	PCR primers	Ccccc ctcgag TTATGTGTTCCGCCTTCCTCTC
Sequence-based reagent	vpa1548-del-chk	this study	PCR primers	aagtagccacatcccaaacgc
Sequence-based reagent	VP2191-del-d	this study	PCR primers	ccccc tctaga GACAATGCGCTGCACGGAAT
Sequence-based reagent	VP2191-del-chk	this study	PCR primers	gatggaaaacggctacacca
Sequence-based reagent	del vp2234(FlhF)-d	this study	PCR primers	CCCCC tctaga GAATACATGCTACGAGCTCAAGG
Sequence-based reagent	del vp2234(FlhF)-chk	this study	PCR primers	GTTTACGGCATGATTGATGGCG
Sequence-based reagent	vp2224-Gly110Ala-cw	this study	PCR primers	gagcaaccaaaatggtgcagttaGCGgctgatatcaacgagctaatcg
Sequence-based reagent	vp2224-Gly110Ala-ccw	this study	PCR primers	CGATTAGCTCGTTGATATCAGCcgcTAACTGCACCATTTTGGTTGCTC
Sequence-based reagent	vp2224-Glu126Ala-cw	this study	PCR primers	agagtgtgaactgccaaaagcaGCAgcagagttgatgctctctttgc
Sequence-based reagent	vp2224-Glu126Ala-ccw	this study	PCR primers	GCAAAGAGAGCATCAACTCTGctgCTGCTTTTGGCAGTTCACACTCT
Sequence-based reagent	vp2224-Leu129Ala-cw	this study	PCR primers	tgaactgccaaaagcagaagcagag GC gatgctctctttgcagaaaaaactg
Sequence-based reagent	vp2224-Leu129Ala-ccw	this study	PCR primers	CAG TTT TTT CTG CAA AGA GAG CAT CGC CTC TGC TTC TGC TTT TGG CAG TTC A
Sequence-based reagent	C-term sfGFP-vp2224-a	this study	PCR primers	CCCCC actagt ATGGCTGAAGAGACTTTTTTATCTGTAC
Sequence-based reagent	C-term sfGFP-vp2224-b	this study	PCR primers	gagctcgaggatgtc TCGTCGACGCCCACGTGG
Sequence-based reagent	C-term sfGFP-vp2224-c	this study	PCR primers	gacatcctcgagctc atgagcaaaggagaagaacttttcac
Sequence-based reagent	C-term sfGFP-vp2224-d	this study	PCR primers	tta tttgtagagctcatccatgcc
Sequence-based reagent	C-term sfGFP-vp2224-e	this study	PCR primers	ggcatggatgagctctacaaa taa AGAGAATAAAAAGAAGCTTCGG
Sequence-based reagent	C-term sfGFP-vp2224-f	this study	PCR primers	ccccc gcatgc TTTGTTTGTCGATTGCTGTTAGTGG
Sequence-based reagent	del AA7-27 vp2224-b	this study	PCR primers	AAAAGTCTCTTCAGCCATCGTCATTC
Sequence-based reagent	del AA7-27 vp2224-c	this study	PCR primers	GAATGACGATGGCTGAAGAGACTTTT CTGCGCATTCGTGCTAGTTTGC
Sequence-based reagent	vp2224-cw-pBAD	this study	PCR primers	CCCCC tctaga atggctgaagagacttttttatctg
Sequence-based reagent	vp2224-ccw-pBAD	this study	PCR primers	CCCCC gcatgc ttatcgtcgacgcccacg
Sequence-based reagent	vp2224 cw restore deletion	this study	PCR primers	ACCTATAATTGGCTGAATGACG ATGGCTGAAGAGACTTTTTTATCTGTAC
Sequence-based reagent	downstream vp2224 cw	this study	PCR primers	AGAGAATAAAAAGAAGCTTCGGC
Sequence-based reagent	pUT18/pKNT25- vp2224-cw	this study	PCR primers	ccccc TCTAGA atggctgaagagacttttttatctgtac
Sequence-based reagent	pUT18/pKNT25- tr-vp2224-cw	this study	PCR primers	ccccc TCTAGA ATG cgcattcgtgctagtttgc
Sequence-based reagent	pUT18/pKNT25-vp2222 -ccw	this study	PCR primers	ccccc GGTACC CG tcgtcgacgcccacgtg
Sequence-based reagent	pUT18C/pKT25-vp2224-cw	this study	PCR primers	ccccc tctaga G gctgaagagacttttttatctgtac
Sequence-based reagent	pUT18C/pKT25-vp2224-ccw	this study	PCR primers	ccccc ggtacc ttatcgtcgacgcccacgtg
Sequence-based reagent	tr2224 put18C cw	this study	PCR primers	ccccc tctaga G ATG cgcattcgtgctagtttgcaaaa
Sequence-based reagent	sfGFP-1-ccw	this study	PCR primers	ccccc tctaga tttgtagagctcatccatgccatg
Sequence-based reagent	vp2224 C-term PhoA-LacZ cw	this study	PCR primers	CCCCC tctaga g atggcccggacaccagaaatg
Sequence-based reagent	end -LacZ w/o STOP ccw	this study	PCR primers	gcgccattcgccattcaggctgc
Sequence-based reagent	LacZ to vp2224 w/o ATG	this study	PCR primers	CCT GAA TGG CGA ATG GCG C GCT GAA GAG ACT TTT TTA TCT GTA CC
Sequence-based reagent	end vp2224 ccw	this study	PCR primers	CCCCC aagctt ttatcgtcgacgcccacgtgg
Sequence-based reagent	vp2224 ccw restore deletion	this study	PCR primers	GCCGAAGCTTCTTTTTATTCTCT TTATCGTCGACGCCCACGTG
Sequence-based reagent	M13	this study	PCR primers	TGTAAAACGACGGCCAGTCC
Sequence-based reagent	M13r	this study	PCR primers	CACACAGGAAACAGCTATGACC
Sequence-based reagent	flhF1-flhG1 fwd	this study	PCR primers	GCGCTGAGTGTGTTGATCCAAA
Sequence-based reagent	EcoRV FliM1 N-term fwd	this study	PCR primers	GCGAATTCGTGGATCCAGATGCTCATTGAAGATGCTCTCCTG
Sequence-based reagent	EcoRV FliM1 N-term rev	this study	PCR primers	GCCAAGCTTCTCTGCAGGATAATAAAACTGCGGCCCACTTCC
Sequence-based reagent	Check-GFP FliM1-fwd	this study	PCR primers	GCAGTTCAGATGAGTCATCCTC
Sequence-based reagent	Check-GFP FliM1 KO-rev	this study	PCR primers	GACATTTTGGCAGTTGATGCGAC
Sequence-based reagent	OL FliM1 GFP rev	this study	PCR primers	GAAAAGTTCTTCTCCTTTGCTGCTGCCTAATTCAGATATATCTCTAGCTTTGCCTTTGC
Sequence-based reagent	OL FliM1 GFP fwd	this study	PCR primers	GGATGAGCTCTACAAAGGATCCTAAGGTGAAGCAAGATGAGCACAGAAGATA
Sequence-based reagent	EcoRV FlhF C-term fwd	this study	PCR primers	GCGAATTCGTGGATCCAGATGCAAGAAATGGTTGGACAGCCT
Sequence-based reagent	EcoRV FlhF C-term rev	this study	PCR primers	GCCAAGCTTCTCTGCAGGATGCCACATCTAAAAATCGGTCGG
Sequence-based reagent	Check-FlhF-FLAG-fwd	this study	PCR primers	GCATCAGTCAATGCAAGCAACC
Sequence-based reagent	OL-FlhF-Venus rev	this study	PCR primers	CACGCTGCCCTCAAATGCACAGGCCATATTATCTG
Sequence-based reagent	OL_Venus fwd	this study	PCR primers	GCATTTGAGGGCAGCGTGAGCAAGGGCGAGGAGCTGTT
Sequence-based reagent	OL_Venus rev	this study	PCR primers	GTCATAACTTTACTTGTACAGCTCGTCCATGCC
Sequence-based reagent	OL-FlhF-Venus fwd	this study	PCR primers	TACAAGTAAAGTTATGACCCTGGATCAAGCAAG
Sequence-based reagent	FlhF-Ven Seq_Primer	this study	PCR primers	GCTGAGTTAGTACGAGCACTAC
Sequence-based reagent	FlhG-Ven Seq_Primer	this study	PCR primers	CGATATTATTGTCCGTGGGCCT
sequence-based reagent	FlhF-Ven Seq_Primer fwd	this study	PCR primers	GCTGTTGTAGTTGTACTCCAGC
sequence-based reagent	EcoRV FlhF C-term rev	this study	PCR primers	GCCAAGCTTCTCTGCAGGATGCCACATCTAAAAATCGGTCGG
sequence-based reagent	EcoRV-2550-GFP-fwd	this study	PCR primers	GCGAATTCGTGGATCCAGATGCCATCAATAACGGAAAAGGGG
sequence-based reagent	OL-2550-GFP-rev	this study	PCR primers	GAAAAGTTCTTCTCCTTTGCTCAGTTCCAGAATATCTTTACGATGTAACCGGATCAATAATTCAGC
sequence-based reagent	OL-2550-GFP-fwd	this study	PCR primers	GGATGAGCTCTACAAAGGATCCTAACGAAGTGTAGGGGCTAAGACG
sequence-based reagent	EcoRV-2550-GFP-rev	this study	PCR primers	GCCAAGCTTCTCTGCAGGATGCCTTTGTTTATATGCTCGACGG
sequence-based reagent	Check-2550-GFP-fwd	this study	PCR primers	CGATGAAGAATGGGCTGAACTC
sequence-based reagent	Check-2550-GFP-rev	this study	PCR primers	CGAAGGATGCGAGAATGACGAA
sequence-based reagent	OL-2069_FlhF-rev	this study	PCR primers	AATCTTCACTAGCATCCCCGTACATTGAACTC
sequence-based reagent	OL-FlhF-Ven-fwd	this study	PCR primers	GGGATGCTAGTGAAGATTAAACGATTTTTTGCCAAAGAC
sequence-based reagent	OL-FlhF-Ven-rev	this study	PCR primers	AACATTAGCTTACTTGTACAGCTCGTCCATGC
sequence-based reagent	OL-2068-fwd	this study	PCR primers	TACAAGTAAGCTAATGTTTTAGGGTCTTACGCG
sequence-based reagent	BACTH 2550 pkT25 fwd	this study	PCR primers	CAGGGTCGACTCTAGAGGGCGATGAATTTTTGATCGCGG
sequence-based reagent	BACTH 2550 pkT25 rev	this study	PCR primers	TTAGTTACTTAGGTACCCGGGGTTTACGATGTAACCGGATCAATAATTCAGC
sequence-based reagent	BACTH 2550 fwd	this study	PCR primers	CTGCAGGTCGACTCTAGAGGGCGATGAATTTTTGATCGCGG
Sequence-based reagent	BACTH 2550 rev	this study	PCR primers	GAGCTCGGTACCCGGGGTTTACGATGTAACCGGATCAATAATTCAGC
Sequence-based reagent	OL_FliM1 mCh rev	this study	PCR primers	TTTGTATAACTCATCCATACCA
Sequence-based reagent	FlhF-Ven Seq_Primer rev	this study	PCR primers	GCTGGAGTACAACTACAACAGC
Sequence-based reagent	OL-GFP-fwd	this study	PCR primers	AGCAAAGGAGAAGAACTTTTC
Sequence-based reagent	OL-GFP-rev	this study	PCR primers	GGATCCTTTGTAGAGCTCATCC
Sequence-based reagent	OL -mCherry fwd	this study	PCR primers	GTTTCCAAAGGGGAAGAGGACA
Sequence-based reagent	pKT25-for	this study	PCR primers	CACTGACGGCGGATATCGACATGTT
Sequence-based reagent	pKT25-rev	this study	PCR primers	CCGCCGGACATCAGCGCCATTC
Sequence-based reagent	pUT18-for	this study	PCR primers	CCAGGCTTTACACTTTATGCTTCC
Sequence-based reagent	pUT18-rev	this study	PCR primers	GACGCGCCTCGGTGCCCACTGC
Sequence-based reagent	pKNT25-for	this study	PCR primers	CCCAGGCTTTACACTTTATGCTTCC
Sequence-based reagent	pKNT25-rev	this study	PCR primers	GTTTTTTTCCTTCGCCACGGCCTTG
Sequence-based reagent	pUT18C-for	this study	PCR primers	CGGCGTGCCGAGCGGACGTTCG
Sequence-based reagent	pUT18C-rev	this study	PCR primers	TCAGCGGGTGTTGGCGGGTGTC
Sequence-based reagent	FlhF Seq_Primer fwd	this study	PCR primers	GCCCACTTTGGATCAACACACT
Sequence-based reagent	FlhF Seq_Primer rev	this study	PCR primers	CGTGCTCACAAAACTCGATGAA
Sequence-based reagent	EcoRV FliFG1 KO fwd	this study	PCR primers	GCGAATTCGTGGATCCAGATGCCGAAAACTTGTGGCTGAAAA
Sequence-based reagent	OL- FliFG1 KO rev	this study	PCR primers	ATCGCCACCCCCGACAATCATTTCTGTGCTC
Sequence-based reagent	OL- FliFG1 KO fwd	this study	PCR primers	ATTGTCGGGGGTGGCGATGAGTTCCTCTAAT
Sequence-based reagent	EcoRV FliFG1 KO rev	this study	PCR primers	GCCAAGCTTCTCTGCAGGATGCAACCTAATAGTCACTGCTTG
Sequence-based reagent	OL-fipA L118A rev	this study	PCR primers	AGCTTCAGCTTTGGGCGCTTCACA
Sequence-based reagent	OL-fipA L118A fwd	this study	PCR primers	ATAAAAGAGTGTGAAGCGCCCAAA
Sequence-based reagent	OL-fipA G106A rev	this study	PCR primers	TTCATCGACTCCCGCGGCAAGTCC
Sequence-based reagent	OL-fipA G106A fwd	this study	PCR primers	AAAATGGTCGGACTTGCCGCGGGA
Sequence-based reagent	OL-PPfipA G104A rev	this study	PCR primers	CATCGATACTCGCAGCCATCCC
Sequence-based reagent	OL-PPfipA G104A fwd	this study	PCR primers	GCTGGTGGGGATGGCTGCGAGT
Sequence-based reagent	OL-PPfipA L123A rev	this study	PCR primers	ACACCTTGCTCATCGCCTCCGC
Sequence-based reagent	OL-PPfipA L123A fwd	this study	PCR primers	GGCCGAGGCGGAGGCGATGAGC
Sequence-based reagent	EcoRV-flhF KO-fwd	this study	PCR primers	GCCAAGCTTCTCTGCAGGATGCATAGGCGTCGGTGATTGAGG
Sequence-based reagent	OL-flhF KO-rev	this study	PCR primers	TAAGTGAAGGCATTTGAGTAGAGTTATGACCCTGG
Sequence-based reagent	OL-flhF KO-fwd	this study	PCR primers	CTCAAATGCCTTCACTATGCGTCCTCTACTGG
Sequence-based reagent	EcoRV-flhF KO-rev	this study	PCR primers	GCGAATTCGTGGATCCAGATGCTAAGCATTCTCCTAAGCTTGTTG
Sequence-based reagent	OL-fipA L125A rev	this study	PCR primers	TAACCGGATCAAGGCTTCAGCTTC
Sequence-based reagent	OL-fipA L125A fwd	this study	PCR primers	GCTGAAGCTGAAGCCTTGATCCGG
Sequence-based reagent	EcoRV FlhF sub rev	this study	PCR primers	GCCAAGCTTCTCTGCAGGATGCTCGTCACATACAACGACTAG
Sequence-based reagent	BACTH 2550 L125A pkT25 rev	this study	PCR primers	TTAGTTACTTAGGTACCCGGGGTTTACGATGTAACCGGATCAAGGCTTCAGC
Sequence-based reagent	BACTH 2550 L125A rev	this study	PCR primers	GAGCTCGGTACCCGGGGTTTACGATGTAACCGGATCAAGGCTTCAGC
Sequence-based reagent	OL-FipA L125A-GFP-rev	this study	PCR primers	GAAAAGTTCTTCTCCTTTGCTCAGTTCCAGAATATCTTTACGATGTAACCGGATCAAGGCTTCAGC
Sequence-based reagent	BACTH FlhF pkT25 fwd	this study	PCR primers	CAGGGTCGACTCTAGAGAAGATTAAACGATTTTTTGCCAAAGACA
Sequence-based reagent	BACTH FlhF pkT25 rev	this study	PCR primers	TTAGTTACTTAGGTACCCGGGGCTCAAATGCACAGGCCATATTATCT
Sequence-based reagent	BACTH FlhF fwd	this study	PCR primers	CTGCAGGTCGACTCTAGAGAAGATTAAACGATTTTTTGCCAAAGACA
Sequence-based reagent	BACTH FlhF rev	this study	PCR primers	GAGCTCGGTACCCGGGGCTCAAATGCACAGGCCATATTATCT
Sequence-based reagent	BACTH FlhF GTG fwd	this study	PCR primers	CTGCAGGTCGACTCTAGAGGTGAAGATTAAACGATTTTTTGCCAAAG
Sequence-based reagent	EcoRV_FipA KO fwd	this study	PCR primers	GCGAATTCGTGGATCCAGATTTTTAGGTATCATTAACTTACGTGGTAATGT
Sequence-based reagent	OL-FipA KO rev	this study	PCR primers	ACACTTCGCTATTTACGATGATCGCCCATTAAAAATCCTTATGCA
Sequence-based reagent	OL-FipA KO fwd	this study	PCR primers	AAGGATTTTTAATGGGCGATCATCGTAAATAGCGAAGTGTAGGG
Sequence-based reagent	EcoRV-FipA KO rev	this study	PCR primers	GCCAAGCTTCTCTGCAGGATGAACTGATCGCCTTTGTTTATATGC
Sequence-based reagent	Check-FipA KO fwd	this study	PCR primers	AAGAAATGTCGCAGCCGTAGC
Sequence-based reagent	Check-FipA KO rev	this study	PCR primers	CCAGTTGCGACAATCTTCGGAG
Sequence-based reagent	OL-PPfipA L116A rev	this study	PCR primers	CATCAACTCCGCCTCGGCCTGGGTCGCGCCGCAGCTCTGGGT
Sequence-based reagent	OL-PPfipA L1164A fwd	this study	PCR primers	GAGTTGACCCAGAGCTGCGGCGCGACCCAGGCCGAGGCG
Sequence-based reagent	Check-PP_4331 (FipA) fwd	this study	PCR primers	GCTTACGAACAGAACGCAAGGC
Sequence-based reagent	Check-PP_4331 (FipA) rev	this study	PCR primers	GCAATACGTGATTTCGGTGCAG
Sequence-based reagent	EcoRV-PP_4331 KO-fwd	this study	PCR primers	GCGAATTCGTGGATCCAGATGCAGATGCACGCCAAACAGAAA
Sequence-based reagent	PP_4331 KO-OL-rev	this study	PCR primers	TCAAGGAGCTAGGATCAACTCAGATGTTCTCCAGC
Sequence-based reagent	PP_4331KO-OL-fwd	this study	PCR primers	TTGATCCTAGCTCCTTGACGGGGTACCCTCG
Sequence-based reagent	EcoRV-PP_4331 KO-rev	this study	PCR primers	GCCAAGCTTCTCTGCAGGATGCATGAATTGCCTGTACAACACCA
Sequence-based reagent	Check-PP_4331KO-fwd	this study	PCR primers	GCGAAACGATCGATCAGGTCGA
Sequence-based reagent	Check-PP_4331KO-rev	this study	PCR primers	GCACCGTAATCGAACACATGTG
Sequence-based reagent	EcoRV-PP_4331-GFP-fwd	this study	PCR primers	GCGAATTCGTGGATCCAGATGCAGATGCACGCCAAACAGAAA
Sequence-based reagent	PP_4331-GFP-OL-rev	this study	PCR primers	GAAAAGTTCTTCTCCTTTGCTCAGTTCCAGAATATCAGGAGCCCGGTACACCTTGCTC
Sequence-based reagent	PP_43310-GFP-OL-fwd	this study	PCR primers	GGATGAGCTCTACAAAGGATCCTGACGGGGTACCCTCGGCAGCA
Sequence-based reagent	EcoRV-PP_4331-GFP-rev	this study	PCR primers	GCCAAGCTTCTCTGCAGGATGCATGAATTGCCTGTACAACACCA
Sequence-based reagent	EcoRV-FlhF-mCh-fwd	this study	PCR primers	GCGAATTCGTGGATCCAGATGCATGGACAGCTTCCGTATCGG
Sequence-based reagent	FlhF-mCh-OL-rev	this study	PCR primers	CTCTTCCCCTTTGGAAACGCTGCCACCCGCTCGCCGTGGGTTGTGA
Sequence-based reagent	FlhF-mCh-OL-fwd	this study	PCR primers	ATGGATGAGTTATACAAATGACCATGAAGCGTGTGCAAAG
Sequence-based reagent	EcoRV-FlhF-mCh-rev	this study	PCR primers	GCCAAGCTTCTCTGCAGGATGCCAACACACGGAAACGGTTCA
Sequence-based reagent	Check-PP_4343 KO-fwd	this study	PCR primers	GCCTGAAATCGAGCCGATCGAA
Sequence-based reagent	Check-PP_4343 KO-rev	this study	PCR primers	GCGTCGGTAATCGAGGTAGGTT
Sequence-based reagent	BACTH PP FipA pkT25 fwd	this study	PCR primers	CAGGGTCGACTCTAGAGATCCTAGAGGTTGCTGTCATCT
Sequence-based reagent	BACTH PP FipA pkT25 rev	this study	PCR primers	TTAGTTACTTAGGTACCCGGGGAGGAGCCCGGTACACCTTGCTC
Sequence-based reagent	BACTH PP FipA fwd	this study	PCR primers	CTGCAGGTCGACTCTAGAGATCCTAGAGGTTGCTGTCATCT
Sequence-based reagent	BACTH PP FipA rev	this study	PCR primers	GAGCTCGGTACCCGGGGAGGAGCCCGGTACACCTTGCTC
Sequence-based reagent	BACTH PP FlhF pkT25 fwd	this study	PCR primers	CAGGGTCGACTCTAGAGCAAGTTAAGCGATTTTTCGCCGC
Sequence-based reagent	BACTH PP FlhF pkT25 rev	this study	PCR primers	TTAGTTACTTAGGTACCCGGGGACCCGCTCGCCGTGGGTTGTGA
Sequence-based reagent	BACTH PP FlhF fwd	this study	PCR primers	CTGCAGGTCGACTCTAGAGCAAGTTAAGCGATTTTTCGCCGC
Sequence-based reagent	BACTH PP FlhF rev	this study	PCR primers	GAGCTCGGTACCCGGGGACCCGCTCGCCGTGGGTTGTGA
Sequence-based reagent	EcoRV FlhF sub fwd	this study	PCR primers	GCGAATTCGTGGATCCAGATGCATCAGTCAATGCAAGCAACC
Sequence-based reagent	Check-FlhF KI/O-fwd	this study	PCR primers	GCCACTGGGTAGTGTCGTAAAA
Sequence-based reagent	OL-FlhF D328A rev	this study	PCR primers	CCCCATACCAGCGGTGGCTATCAATAC
Sequence-based reagent	OL-FlhF D328A fwd	this study	PCR primers	AAGCTAGTATTGATAGCCACCGCTGGT
Sequence-based reagent	EcoRV PPFlhF sub fwd	this study	PCR primers	GCGAATTCGTGGATCCAGATGCATGTTCTGGCGTATCAGGAA
Sequence-based reagent	OL-FlhF K235A rev	this study	PCR primers	GCGCGCGGCCAGCGCGGCCAGGGT
Sequence-based reagent	OL-FlhF K235A fwd	this study	PCR primers	GGCAAGACCACCACCCTGGCCGCGCTGGCCGCG
Sequence-based reagent	EcoRV PPFlhF sub rev	this study	PCR primers	GCCAAGCTTCTCTGCAGGATGCATGCTACCCATGTCTGTTCT
Sequence-based reagent	Check-PP_4343 KI-fwd	this study	PCR primers	GCTACCAGTGATTACCCTGGAG
Sequence-based reagent	EcoRV PPFlhF sub 1 rev	this study	PCR primers	GCCAAGCTTCTCTGCAGGATGCGTCGGTAATCGAGGTAGGTT
Sequence-based reagent	KT2440 FlhF Seq primer rev	this study	PCR primers	GCTGGTGAGCATGGACAGCTTC
Sequence-based reagent	EcoRV-FipA dTM fwd	this study	PCR primers	GCGAATTCGTGGATCCAGATGCCGTAGCTGCAAGTAAAGATG
Sequence-based reagent	OL-FipA dTM rev	this study	PCR primers	CTGCTTTTGTTCATCGCCCATTAAAAATCCTTATGC
Sequence-based reagent	OL-FipA dTM fwd	this study	PCR primers	GGCGATGAACAAAAGCAGTTGAGTAAATTACGTAATAAAGTTG
Sequence-based reagent	OL-PP_FipA dTM rev	this study	PCR primers	GCTGTAGTTCTCTAGGAT CAACTCAGATGTTCTCC
Sequence-based reagent	OL-PP_FipA dTM fwd	this study	PCR primers	ATCCTAGAGAACTACAGCAAGCGCCAGCGCG
Software, algorithm	cellProfiles (R package)	Cameron et al., 2014; Cameron, 2018	https://github.com/ta-cameron/Cell-Profiles	
Software, algorithm	MicrobeJ	Ducret et al., 2016		

## References

[bib1] Ainsaar K, Tamman H, Kasvandik S, Tenson T, Hõrak R (2019). The TonB_m_-PocAB system is required for maintenance of membrane integrity and polar position of flagella in *Pseudomonas putida*. Journal of Bacteriology.

[bib2] Arroyo-Pérez EE, Ringgaard S (2021). Interdependent polar localization of flhf and flhg and their importance for flagellum formation of vibrio parahaemolyticus. Frontiers in Microbiology.

[bib3] Banerjee P, Raghav S, Goswami HN, Jain D (2021). The antiactivator FleN uses an allosteric mechanism to regulate σ^54^-dependent expression of flagellar genes in *Pseudomonas aeruginosa*. Science Advances.

[bib4] Bange G, Petzold G, Wild K, Parlitz RO, Sinning I (2007). The crystal structure of the third signal-recognition particle GTPase FlhF reveals a homodimer with bound GTP. PNAS.

[bib5] Blagotinsek V, Schwan M, Steinchen W, Mrusek D, Hook JC, Rossmann F, Freibert SA, Kratzat H, Murat G, Kressler D, Beckmann R, Beeby M, Thormann KM, Bange G (2020). An ATP-dependent partner switch links flagellar C-ring assembly with gene expression. PNAS.

[bib6] Cameron TA, Anderson-Furgeson J, Zupan JR, Zik JJ, Zambryski PC (2014). Peptidoglycan synthesis machinery in Agrobacterium tumefaciens during unipolar growth and cell division. mBio.

[bib7] Cameron T (2018). Github.

[bib8] Campos-García J, Nájera R, Camarena L, Soberón-Chávez G (2000). The *Pseudomonas aeruginosa* motR gene involved in regulation of bacterial motility. FEMS Microbiology Letters.

[bib9] Chen M, Zhao Z, Yang J, Peng K, Baker MA, Bai F, Lo CJ (2017). Length-dependent flagellar growth of *Vibrio alginolyticus* revealed by real time fluorescent imaging. eLife.

[bib10] Chevance FFV, Hughes KT (2008). Coordinating assembly of a bacterial macromolecular machine. Nature Reviews. Microbiology.

[bib11] Coil DA, Anné J (2010). The role of fimV and the importance of its tandem repeat copy number in twitching motility, pigment production, and morphology in Legionella pneumophila. Archives of Microbiology.

[bib12] Correa NE, Peng F, Klose KE (2005). Roles of the regulatory proteins FlhF and FlhG in the *Vibrio cholerae* flagellar transcription hierarchy. Journal of Bacteriology.

[bib13] Dasgupta N, Ramphal R (2001). Interaction of the antiactivator FleN with the transcriptional activator FleQ regulates flagellar number in *Pseudomonas aeruginosa*. Journal of Bacteriology.

[bib14] Donnenberg MS, Kaper JB (1991). Construction of an eae deletion mutant of enteropathogenic *Escherichia coli* by using a positive-selection suicide vector. Infection and Immunity.

[bib15] Dornes A, Schmidt LM, Mais CN, Hook JC, Pané-Farré J, Kressler D, Thormann KM, Bange G (2024). Polar confinement of a macromolecular machine by an SRP-type GTPase. Nature Communications.

[bib16] Ducret A, Quardokus EM, Brun YV (2016). MicrobeJ, a tool for high throughput bacterial cell detection and quantitative analysis. Nature Microbiology.

[bib17] Fogel MA, Waldor MK (2006). A dynamic, mitotic-like mechanism for bacterial chromosome segregation. Genes & Development.

[bib18] Francis NR, Sosinsky GE, Thomas D, DeRosier DJ (1994). Isolation, characterization and structure of bacterial flagellar motors containing the switch complex. Journal of Molecular Biology.

[bib19] Fredrickson JK, Zachara JM, Kennedy DW, Dong H, Onstott TC, Hinman NW, Li S (1998). Biogenic iron mineralization accompanying the dissimilatory reduction of hydrous ferric oxide by a groundwater bacterium. Geochimica et Cosmochimica Acta.

[bib20] Gao T, Shi M, Ju L, Gao H (2015). Investigation into FlhFG reveals distinct features of FlhF in regulating flagellum polarity in Shewanella oneidensis. Molecular Microbiology.

[bib21] Gibson DG, Young L, Chuang RY, Venter JC, Hutchison CA, Smith HO (2009). Enzymatic assembly of DNA molecules up to several hundred kilobases. Nature Methods.

[bib22] Green JCD, Kahramanoglou C, Rahman A, Pender AMC, Charbonnel N, Fraser GM (2009). Recruitment of the earliest component of the bacterial flagellum to the old cell division pole by a membrane-associated signal recognition particle family GTP-binding protein. Journal of Molecular Biology.

[bib23] Heering J, Ringgaard S (2016). Differential localization of chemotactic signaling arrays during the lifecycle of *Vibrio parahaemolyticus*. Frontiers in Microbiology.

[bib24] Heering J, Alvarado A, Ringgaard S (2017). Induction of cellular differentiation and single cell imaging of vibrio parahaemolyticus swimmer and swarmer cells. Journal of Visualized Experiments.

[bib25] Hendrixson DR, DiRita VJ (2003). Transcription of sigma54-dependent but not sigma28-dependent flagellar genes in Campylobacter jejuni is associated with formation of the flagellar secretory apparatus. Molecular Microbiology.

[bib26] Hintsche M, Waljor V, Großmann R, Kühn MJ, Thormann KM, Peruani F, Beta C (2017). A polar bundle of flagella can drive bacterial swimming by pushing, pulling, or coiling around the cell body. Scientific Reports.

[bib27] Homma M, Aizawa S, Dean GE, Macnab RM (1987). Identification of the M-ring protein of the flagellar motor of *Salmonella typhimurium*. PNAS.

[bib28] Hook JC, Blagotinsek V, Pané-Farré J, Mrusek D, Altegoer F, Dornes A, Schwan M, Schier L, Thormann KM, Bange G (2020). A proline-rich element in the type III secretion protein flhb contributes to flagellar biogenesis in the beta- and gamma-proteobacteria. Frontiers in Microbiology.

[bib29] Iyer SC, Casas-Pastor D, Kraus D, Mann P, Schirner K, Glatter T, Fritz G, Ringgaard S (2020). Transcriptional regulation by σ factor phosphorylation in bacteria. Nature Microbiology.

[bib30] Karimova G, Pidoux J, Ullmann A, Ladant D (1998). A bacterial two-hybrid system based on A reconstituted signal transduction pathway. PNAS.

[bib31] Karimova G, Robichon C, Ladant D (2009). Characterization of YmgF, a 72-residue inner membrane protein that associates with the *Escherichia coli* cell division machinery. Journal of Bacteriology.

[bib32] Kazmierczak BI, Hendrixson DR (2013). Spatial and numerical regulation of flagellar biosynthesis in polarly flagellated bacteria. Molecular Microbiology.

[bib33] Kim YK, McCarter LL (2000). Analysis of the polar flagellar gene system of Vibrio parahaemolyticus. Journal of Bacteriology.

[bib34] Kojima S, Terashima H, Homma M (2020). Regulation of the single polar flagellar biogenesis. Biomolecules.

[bib35] Kondo S, Homma M, Kojima S (2017). Analysis of the GTPase motif of FlhF in the control of the number and location of polar flagella in *Vibrio alginolyticus*. Biophysics and Physicobiology.

[bib36] Kondo S, Imura Y, Mizuno A, Homma M, Kojima S (2018). Biochemical analysis of GTPase FlhF which controls the number and position of flagellar formation in marine Vibrio. Scientific Reports.

[bib37] Kühn MJ, Schmidt FK, Eckhardt B, Thormann KM (2017). Bacteria exploit a polymorphic instability of the flagellar filament to escape from traps. PNAS.

[bib38] Kusumoto A, Kamisaka K, Yakushi T, Terashima H, Shinohara A, Homma M (2006). Regulation of polar flagellar number by the flhF and flhG genes in Vibrio alginolyticus. Journal of Biochemistry.

[bib39] Kusumoto A, Nishioka N, Kojima S, Homma M (2009). Mutational analysis of the GTP-binding motif of FlhF which regulates the number and placement of the polar flagellum in Vibrio alginolyticus. Journal of Biochemistry.

[bib40] Lassak J, Henche AL, Binnenkade L, Thormann KM (2010). ArcS, the cognate sensor kinase in an atypical arc system of *Shewanella oneidensis* MR-1. Applied and Environmental Microbiology.

[bib41] Macnab RM (2003). How bacteria assemble flagella. Annual Review of Microbiology.

[bib42] Makino K, Oshima K, Kurokawa K, Yokoyama K, Uda T, Tagomori K, Iijima Y, Najima M, Nakano M, Yamashita A, Kubota Y, Kimura S, Yasunaga T, Honda T, Shinagawa H, Hattori M, Iida T (2003). Genome sequence of Vibrio parahaemolyticus: a pathogenic mechanism distinct from that of V cholerae. The Lancet.

[bib43] McCarter LL (1995). Genetic and molecular characterization of the polar flagellum of Vibrio parahaemolyticus. Journal of Bacteriology.

[bib44] Miller VL, Mekalanos JJ (1988). A novel suicide vector and its use in construction of insertion mutations: osmoregulation of outer membrane proteins and virulence determinants in *Vibrio cholerae* requires toxR. Journal of Bacteriology.

[bib45] Milton DL, O’Toole R, Horstedt P, Wolf-Watz H (1996). Flagellin A is essential for the virulence of Vibrio anguillarum. Journal of Bacteriology.

[bib46] Minamino T (2014). Protein export through the bacterial flagellar type III export pathway. Biochimica et Biophysica Acta.

[bib47] Moisi M, Jenul C, Butler SM, New A, Tutz S, Reidl J, Klose KE, Camilli A, Schild S (2009). A novel regulatory protein involved in motility of *Vibrio cholerae*. Journal of Bacteriology.

[bib48] Muraleedharan S, Freitas C, Mann P, Glatter T, Ringgaard S (2018). A cell length-dependent transition in MinD-dynamics promotes A switch in division-site placement and preservation of proliferating elongated Vibrio parahaemolyticus swarmer cells. Molecular Microbiology.

[bib49] Murray TS, Kazmierczak BI (2006). FlhF is required for swimming and swarming in *Pseudomonas aeruginosa*. Journal of Bacteriology.

[bib50] Navarrete B, Leal-Morales A, Serrano-Ron L, Sarrió M, Jiménez-Fernández A, Jiménez-Díaz L, López-Sánchez A, Govantes F (2019). Transcriptional organization, regulation and functional analysis of flhF and fleN in *Pseudomonas putida*. PLOS ONE.

[bib51] Nelson KE, Weinel C, Paulsen IT, Dodson RJ, Hilbert H, Martins dos Santos VAP, Fouts DE, Gill SR, Pop M, Holmes M, Brinkac L, Beanan M, DeBoy RT, Daugherty S, Kolonay J, Madupu R, Nelson W, White O, Peterson J, Khouri H, Hance I, Chris Lee P, Holtzapple E, Scanlan D, Tran K, Moazzez A, Utterback T, Rizzo M, Lee K, Kosack D, Moestl D, Wedler H, Lauber J, Stjepandic D, Hoheisel J, Straetz M, Heim S, Kiewitz C, Eisen JA, Timmis KN, Düsterhöft A, Tümmler B, Fraser CM (2002). Complete genome sequence and comparative analysis of the metabolically versatile Pseudomonas putida KT2440. Environmental Microbiology.

[bib52] Pandza S, Baetens M, Park CH, Au T, Keyhan M, Matin A (2000). The G-protein FlhF has a role in polar flagellar placement and general stress response induction in *Pseudomonas putida*. Molecular Microbiology.

[bib53] Parte AC, Sardà Carbasse J, Meier-Kolthoff JP, Reimer LC, Göker M (2020). List of Prokaryotic names with Standing in Nomenclature (LPSN) moves to the DSMZ. International Journal of Systematic and Evolutionary Microbiology.

[bib54] Petersen BD, Liu MS, Podicheti R, Yang AY-P, Simpson CA, Hemmerich C, Rusch DB, van Kessel JC (2021). The polar flagellar transcriptional regulatory network in *Vibrio campbellii* deviates from canonical *Vibrio* species. Journal of Bacteriology.

[bib55] Ringgaard S, Schirner K, Davis BM, Waldor MK (2011). A family of ParA-like ATPases promotes cell pole maturation by facilitating polar localization of chemotaxis proteins. Genes & Development.

[bib56] Ringgaard S, Zepeda-Rivera M, Wu X, Schirner K, Davis BM, Waldor MK (2014). ParP prevents dissociation of CheA from chemotactic signaling arrays and tethers them to a polar anchor. PNAS.

[bib57] Rossmann F, Brenzinger S, Knauer C, Dörrich AK, Bubendorfer S, Ruppert U, Bange G, Thormann KM (2015). The role of FlhF and HubP as polar landmark proteins in Shewanella putrefaciens CN-32. Molecular Microbiology.

[bib58] Rossmann FM, Rick T, Mrusek D, Sprankel L, Dörrich AK, Leonhard T, Bubendorfer S, Kaever V, Bange G, Thormann KM (2019). The GGDEF domain of the phosphodiesterase PdeB in Shewanella putrefaciens Mediates Recruitment by the Polar Landmark Protein HubP. Journal of Bacteriology.

[bib59] Schuhmacher JS, Rossmann F, Dempwolff F, Knauer C, Altegoer F, Steinchen W, Dörrich AK, Klingl A, Stephan M, Linne U, Thormann KM, Bange G (2015a). MinD-like ATPase FlhG effects location and number of bacterial flagella during C-ring assembly. PNAS.

[bib60] Schuhmacher JS, Thormann KM, Bange G (2015b). How bacteria maintain location and number of flagella?. FEMS Microbiology Reviews.

[bib61] Schwan M, Khaledi A, Willger S, Papenfort K, Glatter T, Häußler S, Thormann KM (2022). FlrA-independent production of flagellar proteins is required for proper flagellation in Shewanella putrefaciens. Molecular Microbiology.

[bib62] Simon R, Priefer U, Pühler A (1983). A Broad Host Range Mobilization System for In Vivo Genetic Engineering: Transposon Mutagenesis in Gram Negative Bacteria. Bio/Technology.

[bib63] Takekawa N, Kwon S, Nishioka N, Kojima S, Homma M (2016). HubP, a polar landmark protein, regulates flagellar number by assisting in the proper polar localization of FlhG in Vibrio alginolyticus. Journal of Bacteriology.

[bib64] Turriziani B, Garcia-Munoz A, Pilkington R, Raso C, Kolch W, von Kriegsheim A (2014). On-beads digestion in conjunction with data-dependent mass spectrometry: A shortcut to quantitative and dynamic interaction proteomics. Biology.

[bib65] Ueno T, Oosawa K, Aizawa S (1992). M ring, S ring and proximal rod of the flagellar basal body of *Salmonella typhimurium* are composed of subunits of a single protein, FliF. Journal of Molecular Biology.

[bib66] Wehbi H, Portillo E, Harvey H, Shimkoff AE, Scheurwater EM, Howell PL, Burrows LL (2011). The peptidoglycan-binding protein FimV promotes assembly of the *Pseudomonas aeruginosa* type IV pilus secretin. Journal of Bacteriology.

[bib67] Yamaichi Y, Bruckner R, Ringgaard S, Möll A, Cameron DE, Briegel A, Jensen GJ, Davis BM, Waldor MK (2012). A multidomain hub anchors the chromosome segregation and chemotactic machinery to the bacterial pole. Genes & Development.

[bib68] Zhang K, He J, Cantalano C, Guo Y, Liu J, Li C (2020). FlhF regulates the number and configuration of periplasmic flagella in Borrelia burgdorferi. Molecular Microbiology.

[bib69] Zheng L, Baumann U, Reymond JL (2004). An efficient one-step site-directed and site-saturation mutagenesis protocol. Nucleic Acids Research.

