# Supplementary figures and images for: A conserved cell-pole determinant organizes proper polar flagellum formation
Source: eLife. 2024 Dec 5;13:RP93004. doi: 10.7554/eLife.93004 (PMC11620751; doi:10.7554/eLife.93004)

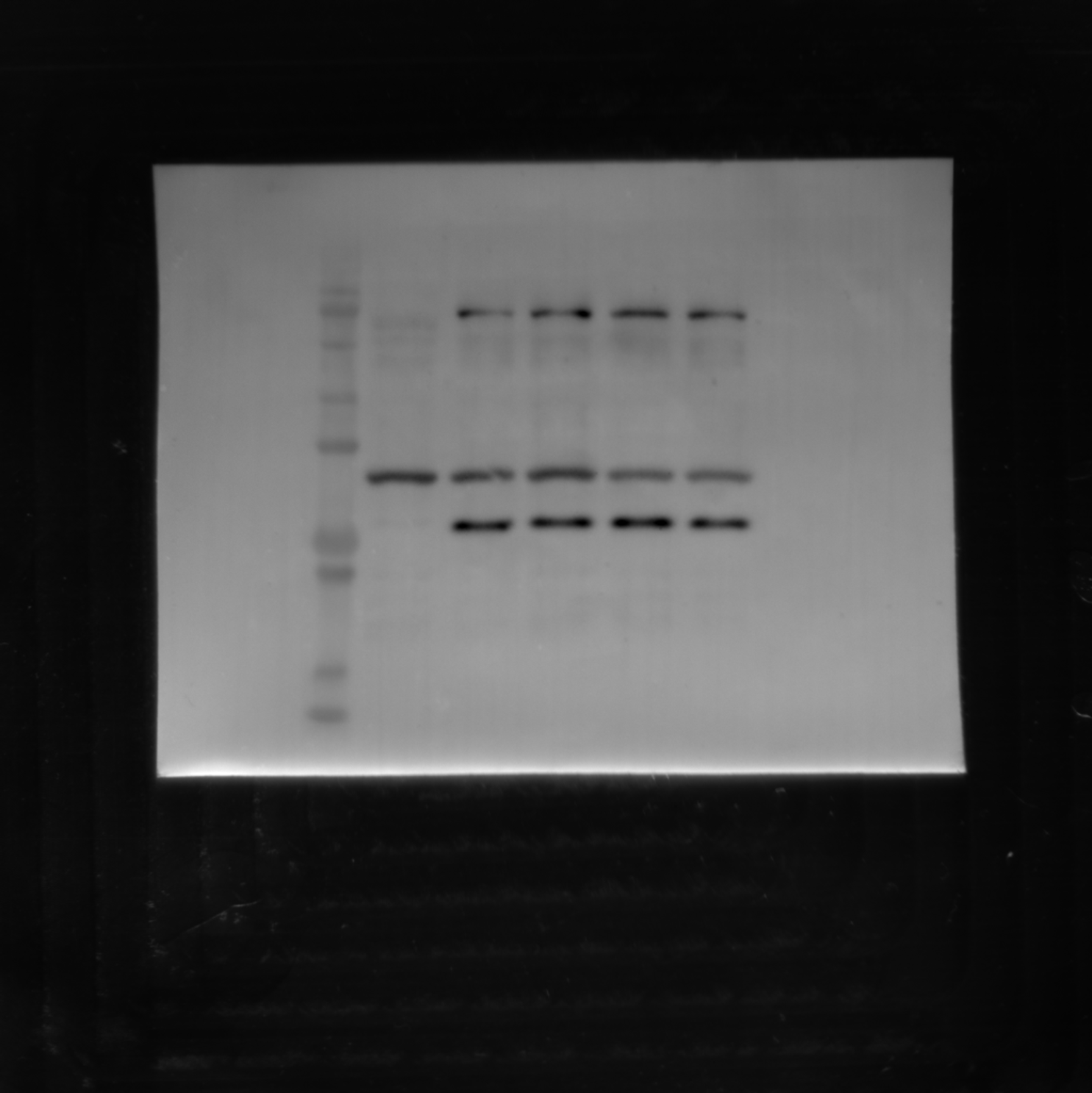

Supplement: Figure 3—figure supplement 1—source data 1. [file elife-93004-fig3-figsupp1-data1.zip › Figure 3-figure supplement 1B-source data 1.tiff]

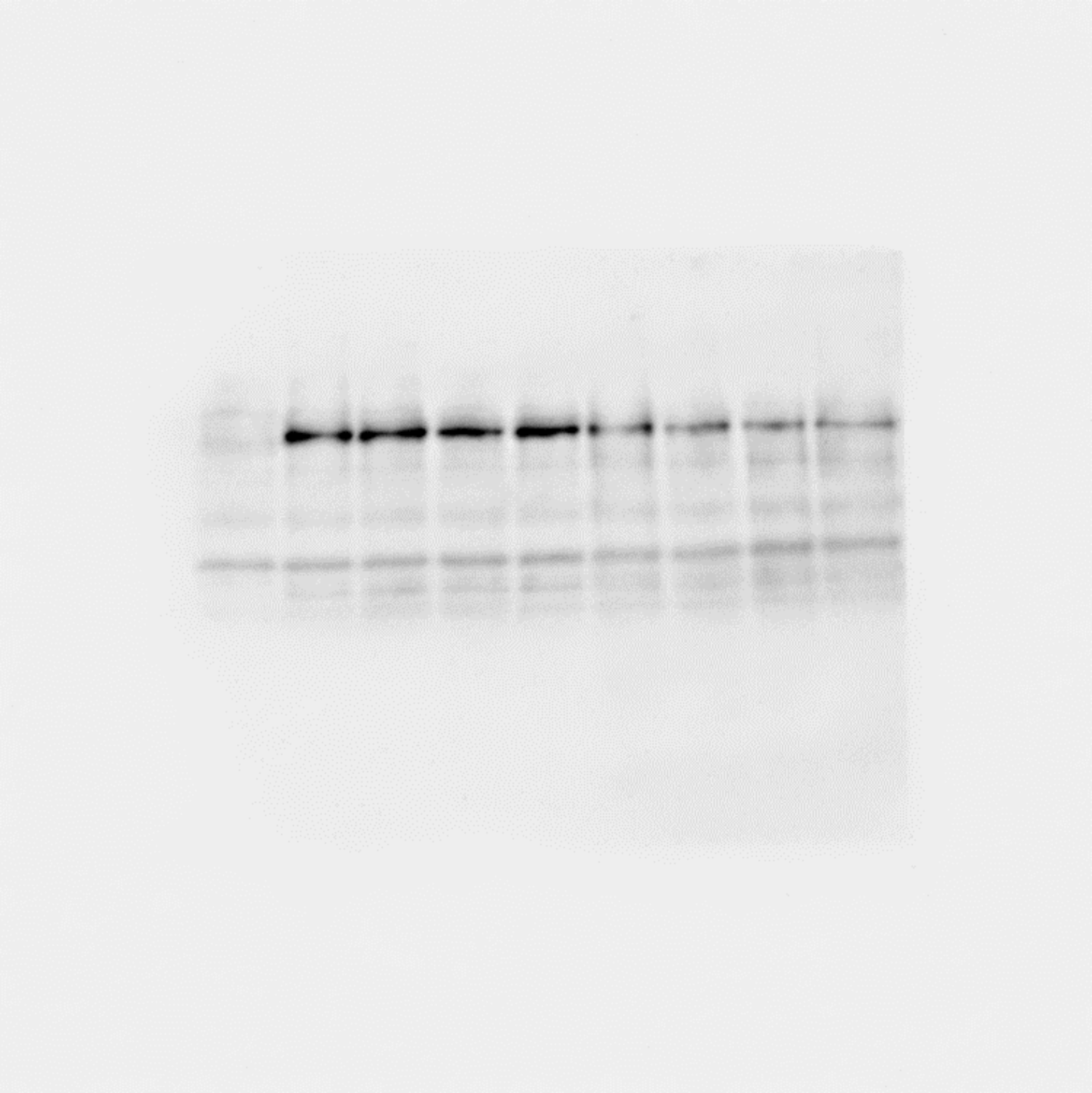

Supplement: Figure 3—figure supplement 1—source data 1. [file elife-93004-fig3-figsupp1-data1.zip › Figure 3-figure supplement 1C-source data 1.tiff]

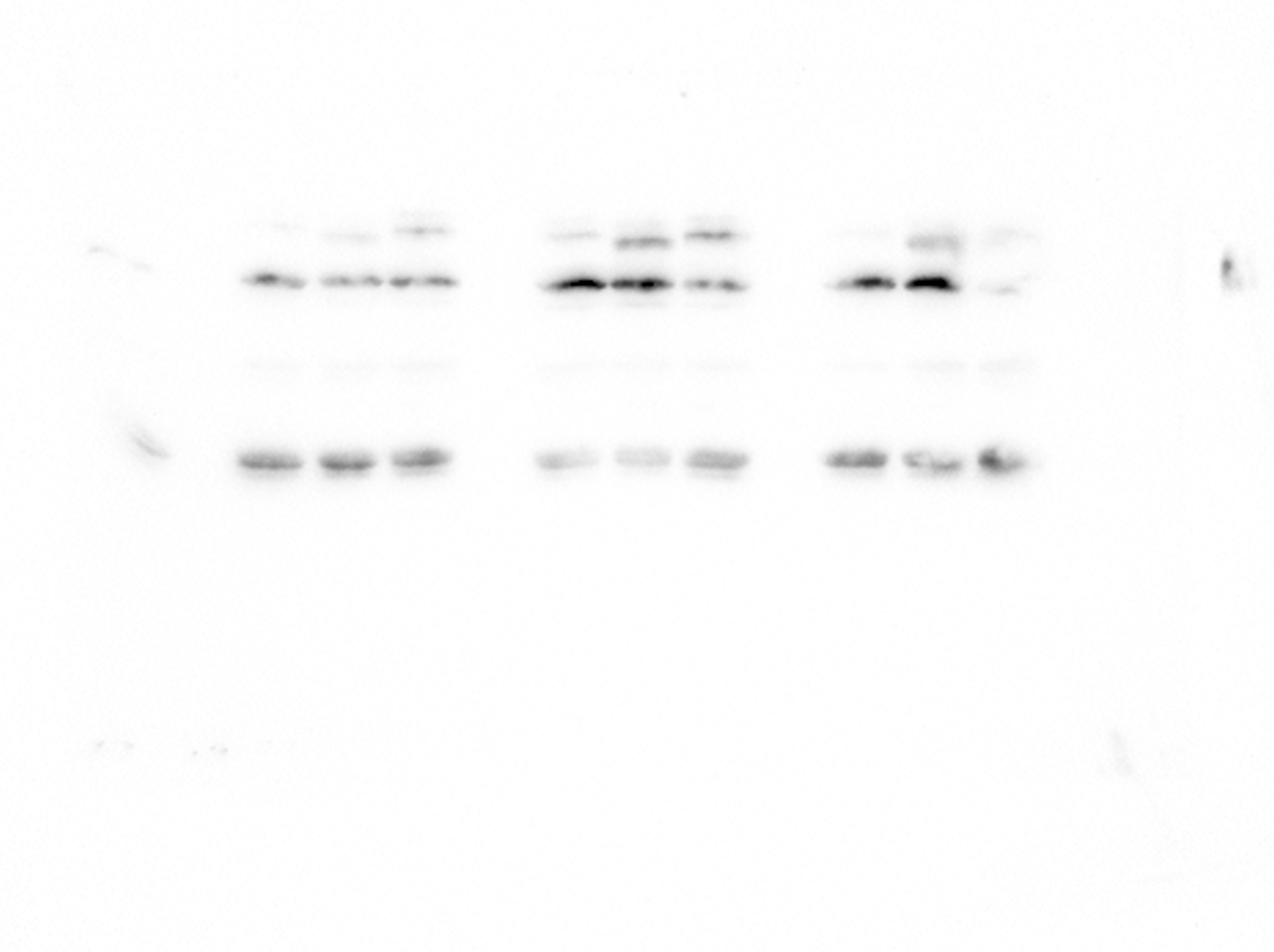

Supplement: Figure 3—figure supplement 1—source data 1. [file elife-93004-fig3-figsupp1-data1.zip › Figure 3-figure supplement 1D-source data 1.tif]

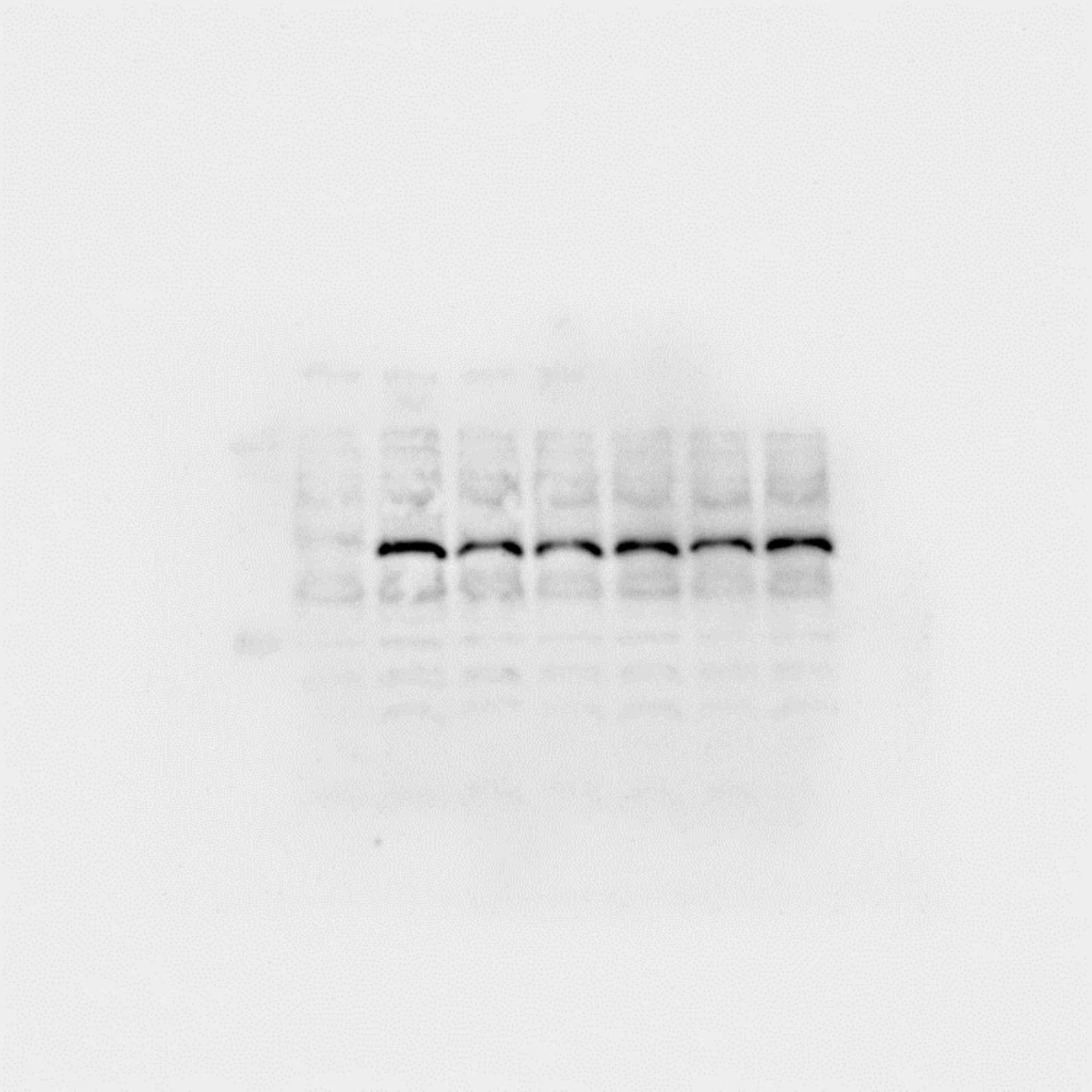

Supplement: Figure 3—figure supplement 1—source data 1. [file elife-93004-fig3-figsupp1-data1.zip › Figure 3-figure supplement 1E-source data 1.tiff]

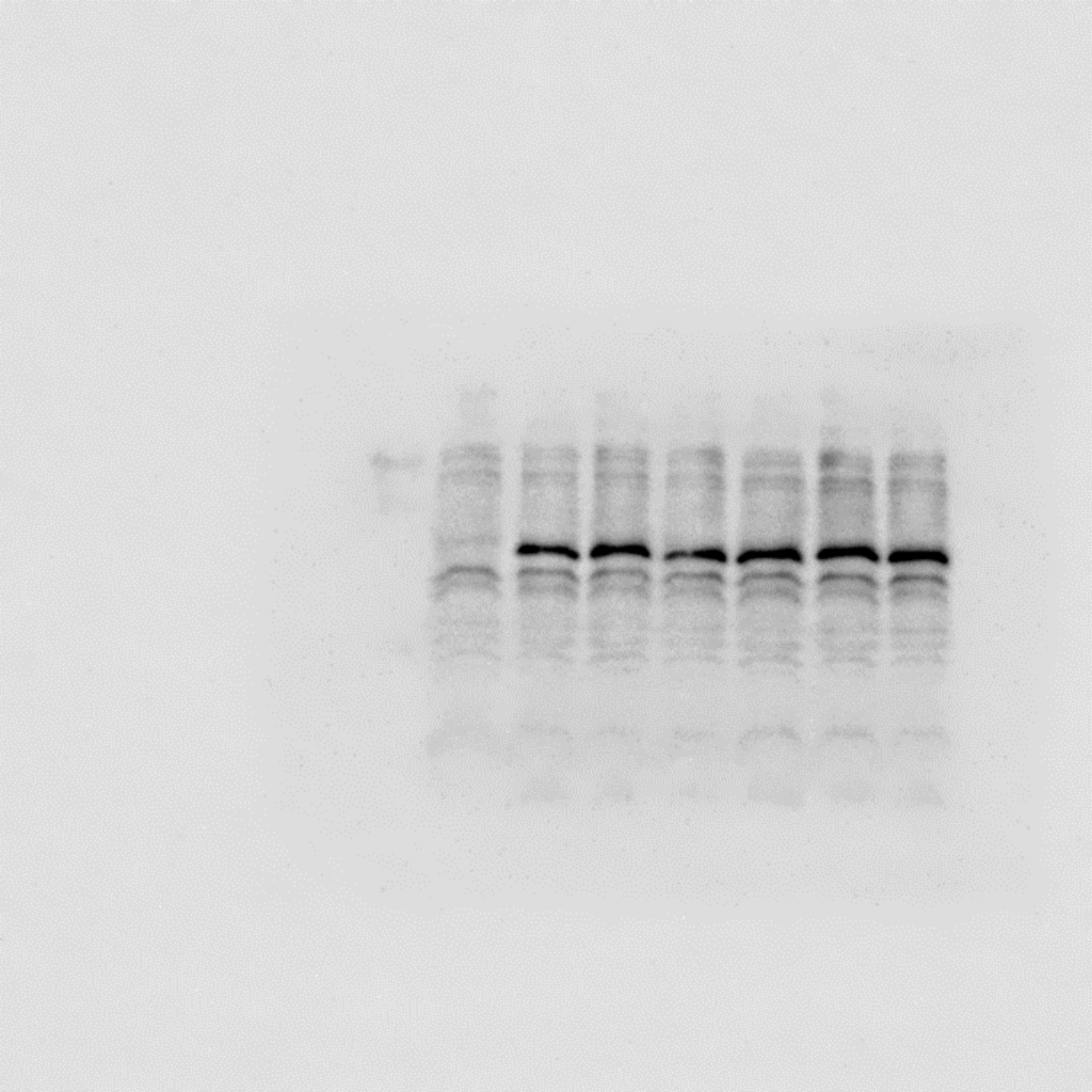

Supplement: Figure 3—figure supplement 1—source data 1. [file elife-93004-fig3-figsupp1-data1.zip › Figure 3-figure supplement 1F-source data 1.tiff]

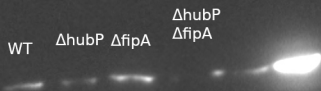

Supplement: Figure 3—figure supplement 1—source data 2. [file elife-93004-fig3-figsupp1-data2.zip › Figure 3-figure supplement 1A-source data 2.pdf]

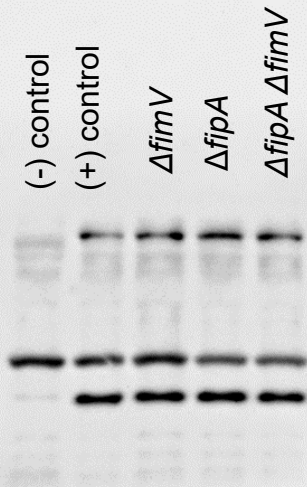

FlhF-mCherry  
~74 kDa

(-) control = *P. putida* wild type

(+) control = FlhF-mCherry

**Date: 16.12.21**

Supplement: Figure 3—figure supplement 1—source data 2. [file elife-93004-fig3-figsupp1-data2.zip › Figure 3-figure supplement 1B-source data 2.pdf]

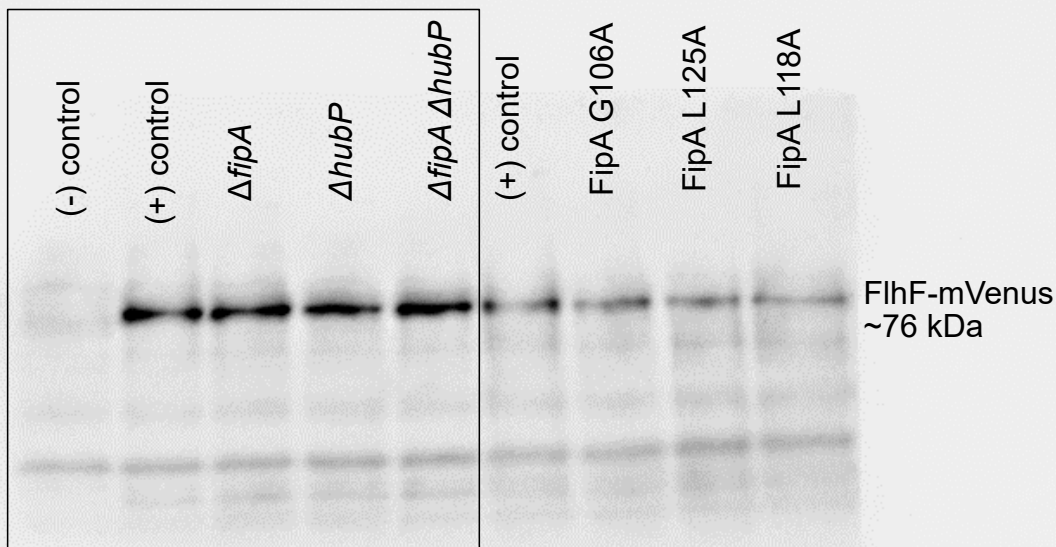

used for Fig.3-Fig Suppl. 1

(-) control = *S. putrefaciens* wild type

(+) control = FlhF-mVenus

**Date: 29.03.22**

Supplement: Figure 3—figure supplement 1—source data 2. [file elife-93004-fig3-figsupp1-data2.zip › Figure 3-figure supplement 1C-source data 2.pdf]

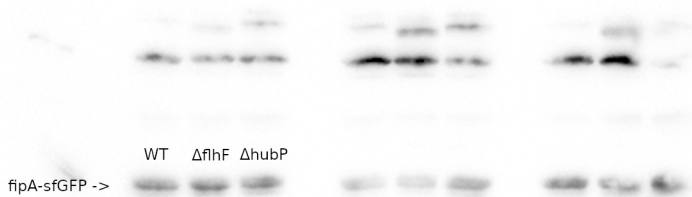

Supplement: Figure 3—figure supplement 1—source data 2. [file elife-93004-fig3-figsupp1-data2.zip › Figure 3-figure supplement 1D-source data 2.pdf]

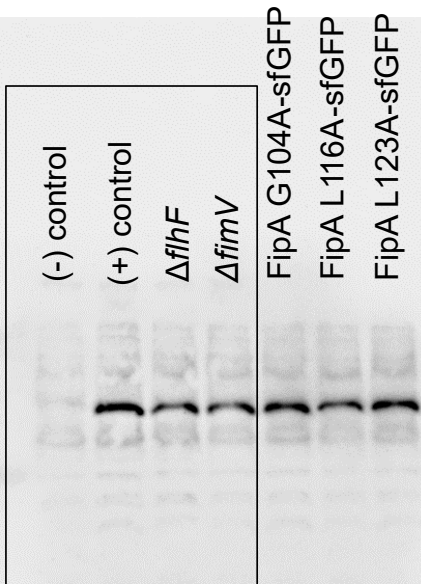

used for Figure 3-figure supplement 1E

(-) control = *P. putida* wild type

(+) control = FipA-sfGFP

**Date: 30.09.21**

Supplement: Figure 3—figure supplement 1—source data 2. [file elife-93004-fig3-figsupp1-data2.zip › Figure 3-figure supplement 1E-source data 2.pdf]

WT  $\Delta$ f1hF fipA fipA  
G110A L129A

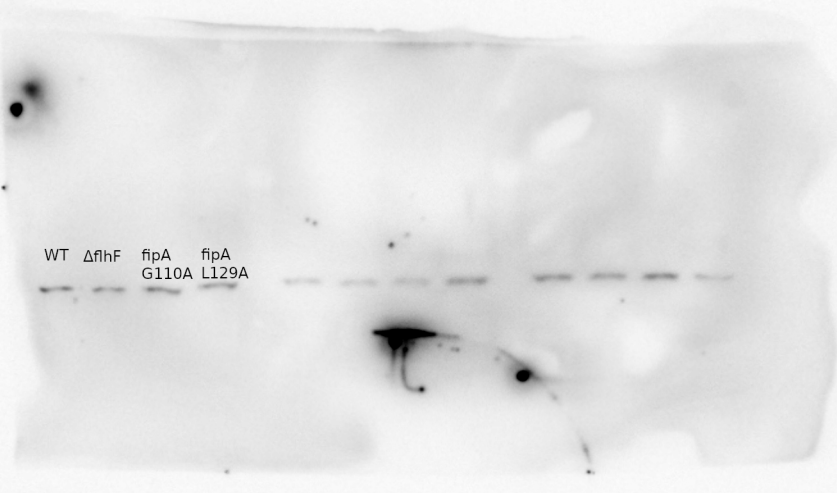

Supplement: Figure 6—figure supplement 1—source data 2. [file elife-93004-fig6-figsupp1-data2.zip › Figure 6-figure supplement 1A-source data 2.pdf]

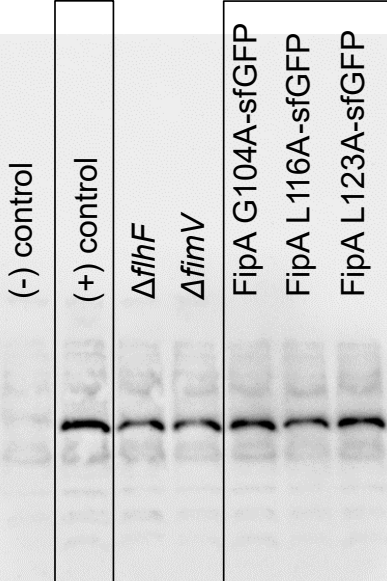

used for Figure 6-figure supplement 1B

(-) control = *P. putida* wild type

(+) control = FipA-sfGFP

**Date: 30.09.21**

Supplement: Figure 6—figure supplement 1—source data 2. [file elife-93004-fig6-figsupp1-data2.zip › Figure 6-figure supplement 1B-source data 2.pdf]

used for Figure 6  
supplement 1C

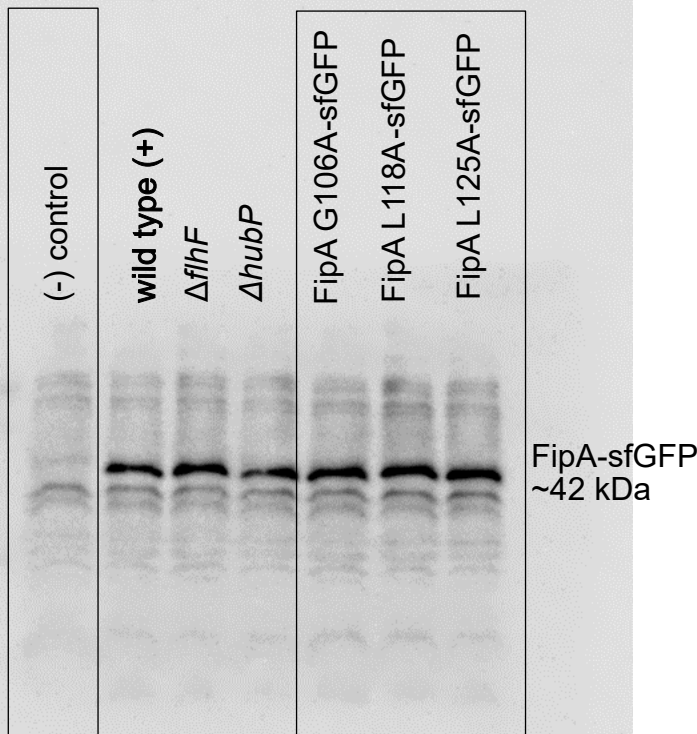

(-) control = *S. putrefaciens* wild type

(+) wild-type background & FipA-sfGFP

Supplement: Figure 6—figure supplement 1—source data 2. [file elife-93004-fig6-figsupp1-data2.zip › Figure 6-figure supplement 1C-source data 2.pdf]
