# Supplementary material for: A conserved cell-pole determinant organizes proper polar flagellum formation
Source: eLife. 2024 Dec 5;13:RP93004. doi: 10.7554/eLife.93004 (PMC11620751; doi:10.7554/eLife.93004)
Supplement: Figure 3—figure supplement 1—source data 2. [file elife-93004-fig3-figsupp1-data2.zip › Figure 3-figure supplement 1F-source data 2.pdf]

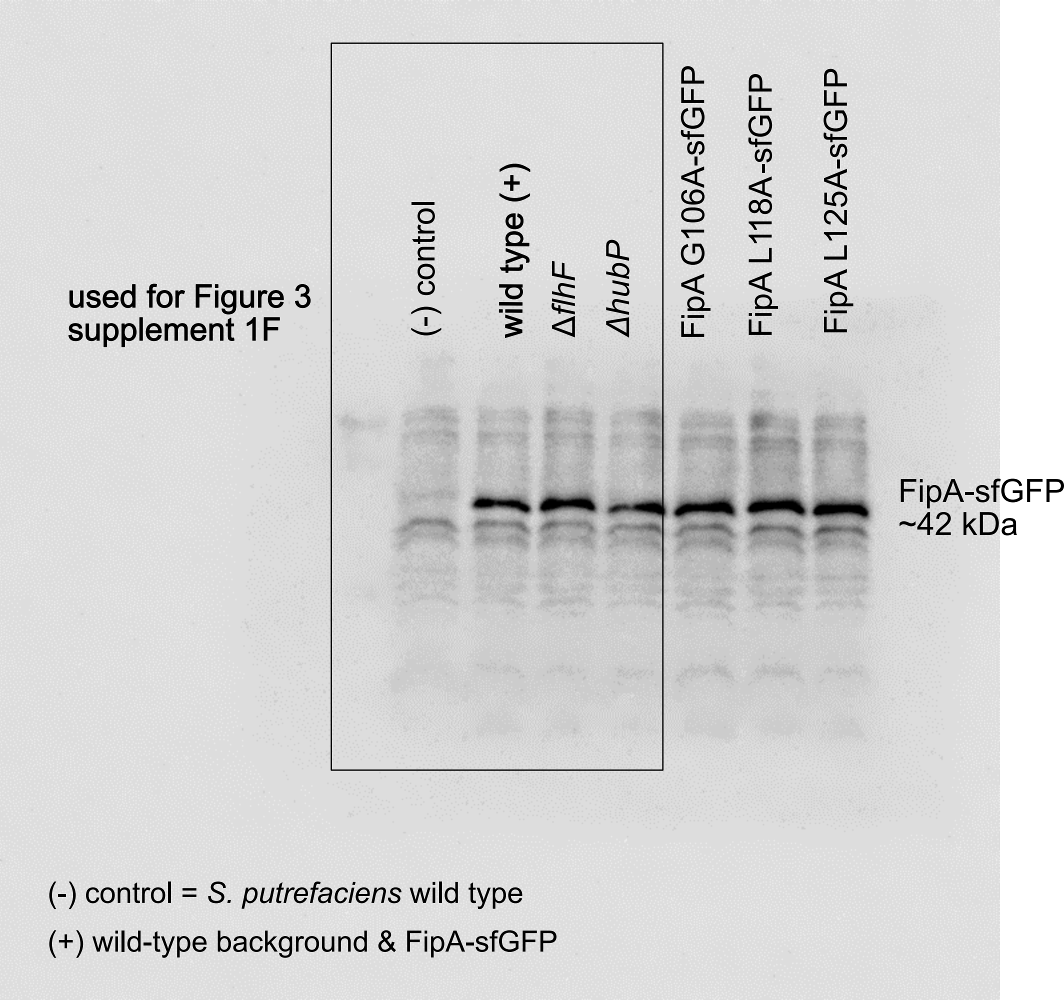
used for Figure 3 supplement 1F
(-) control
wild type (+)
ΔflhF
ΔhubP
FipA G106A-sfGFP
FipA L118A-sfGFP
FipA L125A-sfGFP
FipA-sfGFP
~42 kDa
(-) control = S. putrefaciens wild type
(+) wild-type background & FipA-sfGFP

Date: 01.09.21
